# Supplementary material for: Elevation and latitude drives structure and tree species composition in Andean forests: Results from a large-scale plot network
Source: PLoS One. 2020 Apr 20;15(4):e0231553. doi: 10.1371/journal.pone.0231553 (PMC7170706; doi:10.1371/journal.pone.0231553)
Supplement: S1 Table — aCountry codes are AR, Argentina; BO, Bolivia; PE, Peru; EC, Ecuador; CO, Colombia; VE, Venezuela. cPlot shape refer to R, rectangular; Q, quadrate, and I, irregular; and dimensions refer to length (L) and width (W); dMean annual rainfall and temperature derived from Chelsa, eTime since last disturbance at plot establishment. Number of stems and basal area were extrapolated to 1-ha. *Number of stems, basal area and species richness were estimated for individual’s ≥ 10 cm DBH for comparison. (DOCX) [file pone.0231553.s001.docx]

**Table S1. Andean Forest Network extended metadata**. *^a^*Country codes are AR, Argentina; BO, Bolivia; PE, Peru; EC, Ecuador; CO, Colombia; VE, Venezuela. *^c^*Plot shape refer to R, rectangular; Q, quadrate, and I, irregular; and dimensions refer to length (L) and width (W); *^d^* Total annual rainfall and mean annual temperature derived from Chelsa, *^e^*Time since last disturbance at plot establishment. Number of stems and basal area were extrapolated to 1-ha. *Number of stems, basal area and species richness were estimated for individual’s ≥ 10 cm DBH for comparison.

| **Country^a^** | **Name** | **State^b^** | **Site** | **Location** | | **Elevation** | **Area** | **Plot^c^** | |  | **Rainfall^d^** | **Temperature^d^** | **Censuses** | | | **Min DBH** | **Stems*** | **Basal area*** | **Spp richness*** | **Last disturbance^e^** | **Principal researcher** | **Institution** |
| --- | --- | --- | --- | --- | --- | --- | --- | --- | --- | --- | --- | --- | --- | --- | --- | --- | --- | --- | --- | --- | --- | --- |
|  |  |  |  | S | W | m asl | ha | Shape | dimensions (m) | Permanent Plot | mm | °C | # | yrs | interval yr | cm | (ha-1) | (m^2^ ha-1) | # | yrs |  |  |
| AR | ac.01 | SAL | Acambuco | -22.02 | -63.90 | 1181 | 1 | R | 500 x 20 | P | 1268 | 18.4 | 1 | 2004 | -- | 10 | 565 | 28.26 | 41 | -- | Blundo | FPY |
| AR | ac.02 | SAL | Acambuco | -22.04 | -63.92 | 1014 | 1 | R | 500 x 20 | P | 1172 | 19.4 | 1 | 2004 | -- | 10 | 514 | 19.87 | 43 | -- | Blundo | FPY |
| AR | ag | SAL | Orán | -23.12 | -64.47 | 465 | 1 | R | 500 x 20 | P | 1209 | 21.2 | 2 | 2003 2008 | 5 | 10 | 531 | 27.30 | 49 | 18 | Blundo | FPY |
| AR | ap | SAL | San Andrés | -23.06 | -64.85 | 1984 | 1 | R | 500 x 20 | P | 988 | 14.6 | 2 | 2003 2012 | 9 | 10 | 832 | 38.83 | 20 | -- | Blundo | FPY |
| AR | ba | SAL | PN Baritú | -22.46 | -64.74 | 1684 | 1 | R | 500 x 20 | P | 1024 | 15.8 | 2 | 2003 2012 | 9 | 10 | 693 | 30.43 | 31 | -- | Blundo | FPY |
| AR | ba.a | SAL | PN Baritú | -22.46 | -64.74 | 1973 | 1 | R | 500 x 20 | P | 1097 | 14.5 | 2 | 2003 2012 | 9 | 10 | 670 | 33.32 | 20 | -- | Blundo | FPY |
| AR | bla | SAL | San Andrés | -23.09 | -64.85 | 2134 | 1 | R | 500 x 20 | P | 1267 | 12.8 | 2 | 2003 2012 | 9 | 10 | 845 | 36.71 | 4 | -- | Blundo | FPY |
| AR | bmI | SAL | Bajo Macueta | -22.13 | -63.90 | 829 | 1 | R | 500 x 20 | P | 1173 | 20.1 | 2 | 2005 2009 | 4 | 10 | 507 | 22.75 | 43 | 12 | Blundo | FPY |
| AR | bmII | SAL | Bajo Macueta | -22.10 | -63.90 | 778 | 1 | R | 500 x 20 | P | 1044 | 20.5 | 2 | 2005 2009 | 4 | 10 | 522 | 23.10 | 38 | 12 | Blundo | FPY |
| AR | cb | SAL | Los Naranjos | -23.09 | -64.75 | 1136 | 1 | R | 500 x 20 | P | 1103 | 18.6 | 2 | 2003 2012 | 9 | 10 | 443 | 28.15 | 36 | -- | Blundo | FPY |
| AR | cc | SAL | Aguas Blancas | -22.63 | -64.45 | 572 | 1 | R | 500 x 20 | P | 932 | 21.2 | 2 | 2003 2008 | 5 | 10 | 526 | 22.61 | 33 | 15 | Blundo | FPY |
| AR | cn | SAL | Campichuelo | -22.92 | -64.14 | 619 | 1 | R | 500 x 20 | P | 1244 | 20.8 | 1 | 2008 | -- | 10 | 473 | 29.41 | 42 | 15 | Blundo | FPY |
| AR | km25 | SAL | Piquirenda | -22.27 | -63.84 | 845 | 1 | R | 500 x 20 | P | 915 | 19.8 | 2 | 2005 2009 | 4 | 10 | 451 | 22.68 | 46 | 25 | Blundo | FPY |
| AR | km34 | SAL | Chorrillo | -22.22 | -63.88 | 827 | 1 | R | 500 x 20 | P | 1167 | 19.6 | 2 | 2005 2009 | 4 | 10 | 517 | 19.74 | 42 | 12 | Blundo | FPY |
| AR | km55 | SAL | Acambuco | -22.13 | -63.95 | 990 | 1 | R | 500 x 20 | P | 1119 | 19.6 | 1 | 2011 | -- | 10 | 508 | 19.53 | 45 | -- | Blundo | FPY |
| AR | lc | SAL | San Andrés | -23.08 | -64.80 | 1486 | 1 | R | 500 x 20 | P | 1095 | 17.3 | 2 | 2003 2012 | 9 | 10 | 711 | 34.92 | 34 | -- | Blundo | FPY |
| AR | li | SAL | PN Baritú | -22.44 | -64.73 | 1175 | 1 | R | 500 x 20 | P | 724 | 18.2 | 2 | 2003 2012 | 9 | 10 | 566 | 21.75 | 52 | -- | Blundo | FPY |
| AR | ma | SAL | Los Naranjos | -23.09 | -64.74 | 982 | 1 | R | 500 x 20 | P | 1102 | 18.7 | 2 | 2003 2012 | 9 | 10 | 495 | 28.49 | 38 | -- | Blundo | FPY |
| AR | ms | SAL | Chorrillo | -22.21 | -63.94 | 996 | 1 | R | 500 x 20 | P | 1241 | 19.2 | 2 | 2004 2009 | 5 | 10 | 471 | 32.16 | 39 | 15 | Blundo | FPY |
| AR | no | SAL | RP El Nogalar | -22.28 | -64.72 | 1650 | 1 | R | 500 x 20 | P | 946 | 15.7 | 2 | 2002 2012 | 10 | 10 | 743 | 27.34 | 19 | -- | Blundo | FPY |
| AR | no.a | SAL | RP El Nogalar | -22.27 | -64.75 | 2200 | 1 | R | 500 x 20 | P | 903 | 13.7 | 1 | 2002 | -- | 10 | 376 | 43.93 | 12 | -- | Blundo | FPY |
| AR | rsI | SAL | Río Seco | -22.45 | -63.97 | 596 | 1 | R | 500 x 20 | P | 776 | 21.2 | 2 | 2004 2009 | 5 | 10 | 388 | 23.00 | 33 | 5 | Blundo | FPY |
| AR | rsII | SAL | Río Seco | -22.55 | -63.93 | 711 | 1 | R | 500 x 20 | P | 890 | 20.6 | 2 | 2004 2009 | 5 | 10 | 414 | 18.67 | 43 | 30 | Blundo | FPY |
| AR | sa | SAL | Río Seco | -22.57 | -64.04 | 609 | 1 | R | 500 x 20 | P | 1094 | 21.2 | 2 | 2005 2009 | 4 | 10 | 453 | 23.98 | 36 | 5 | Blundo | FPY |
| AR | te | SAL | Orán | -22.93 | -64.45 | 524 | 1 | R | 500 x 20 | P | 1210 | 21.7 | 2 | 2003 2008 | 5 | 10 | 605 | 31.10 | 43 | 20 | Blundo | FPY |
| AR | to | SAL | San Andrés | -23.07 | -64.79 | 1616 | 1 | R | 500 x 20 | P | 1221 | 15.5 | 2 | 2003 2012 | 9 | 10 | 480 | 44.06 | 32 | -- | Blundo | FPY |
| AR | vm | SAL | Urundel | -23.44 | -64.52 | 677 | 1 | R | 500 x 20 | P | 1198 | 20.6 | 2 | 2003 2008 | 5 | 10 | 678 | 30.80 | 50 | 20 | Blundo | FPY |
| AR | vm.a | SAL | Urundel | -23.44 | -64.67 | 934 | 1 | R | 500 x 20 | P | 1225 | 19.1 | 1 | 2003 | -- | 10 | 479 | 19.56 | 38 | -- | Blundo | FPY |
| AR | ab | JUJ | El Piquete | -24.13 | -64.57 | 654 | 1 | R | 500 x 20 | P | 608 | 19.5 | 1 | 2008 | -- | 10 | 436 | 13.40 | 28 | 7 | Blundo | FPY |
| AR | ai | JUJ | Fraile Pintado | -24.01 | -64.94 | 735 | 1 | R | 500 x 20 | P | 807 | 20.4 | 2 | 2003 2008 | 5 | 10 | 425 | 21.11 | 39 | 15 | Blundo | FPY |
| AR | cr | JUJ | RP Yala | -24.11 | -65.49 | 2140 | 1 | R | 500 x 20 | P | 901 | 13 | 1 | 2010 | -- | 10 | 121 | 5.44 | 4 | -- | Blundo | FPY |
| AR | es | JUJ | Escaleras | -24.12 | -65.10 | 1548 | 1 | R | 500 x 20 | P | 936 | 14.7 | 2 | 2003 2012 | 9 | 10 | 524 | 29.64 | 28 | -- | Blundo | FPY |
| AR | fo | JUJ | Fraile Pintado | -23.99 | -65.06 | 1084 | 1 | R | 500 x 20 | P | 991 | 17.7 | 1 | 2003 | -- | 10 | 497 | 23.56 | 37 | -- | Blundo | FPY |
| AR | ho | JUJ | Palpalá | -24.19 | -65.14 | 1159 | 1 | R | 500 x 20 | P | 610 | 16.9 | 1 | 2003 | -- | 10 | 410 | 29.03 | 31 | -- | Blundo | FPY |
| AR | lq | JUJ | Gral. San Martín | -23.77 | -64.46 | 473 | 1 | R | 500 x 20 | P | 1042 | 21 | 1 | 2009 | -- | 10 | 463 | 19.28 | 34 | 5 | Blundo | FPY |
| AR | me | JUJ | PN Calilegua | -23.70 | -64.87 | 1082 | 1 | R | 500 x 20 | P | 1056 | 17.2 | 2 | 2003 2012 | 9 | 10 | 493 | 34.67 | 37 | -- | Blundo | FPY |
| AR | mo | JUJ | PN Calilegua | -23.68 | -64.90 | 1747 | 1 | R | 500 x 20 | P | 1121 | 15.4 | 2 | 2003 2012 | 9 | 10 | 584 | 36.47 | 27 | -- | Blundo | FPY |
| AR | oc | JUJ | Ocloyas | -23.97 | -65.28 | 1961 | 1 | R | 500 x 20 | P | 998 | 14 | 1 | 2003 | -- | 10 | 654 | 33.12 | 16 | -- | Blundo | FPY |
| AR | ph | JUJ | San Fransisco | -23.63 | -64.93 | 2304 | 1 | R | 500 x 20 | P | 860 | 11.8 | 2 | 2003 2012 | 9 | 10 | 221 | 42.40 | 13 | -- | Blundo | FPY |
| AR | ro | JUJ | RP Yala | -24.10 | -65.48 | 2166 | 1 | R | 500 x 20 | P | 952 | 12.5 | 1 | 2010 | -- | 10 | 192 | 10.61 | 5 | -- | Blundo | FPY |
| AR | sau | JUJ | Gral. San Martín | -23.65 | -64.54 | 396 | 1 | R | 500 x 20 | P | 1094 | 21.2 | 1 | 2007 | -- | 10 | 290 | 14.03 | 24 | 3 | Blundo | FPY |
| AR | sm | JUJ | Gral. San Martín | -23.77 | -64.80 | 595 | 1 | R | 500 x 20 | P | 1007 | 21.5 | 3 | 2002 2008 2013 | 5.5 | 10 | 487 | 22.60 | 28 | 25 | Blundo | FPY |
| AR | ta | JUJ | El Talar | -23.22 | -64.46 | 521 | 1 | R | 500 x 20 | P | 1190 | 21.3 | 2 | 2003 2008 | 5 | 10 | 455 | 26.67 | 42 | 25 | Blundo | FPY |
| AR | yu | JUJ | Gral. San Martín | -23.94 | -64.91 | 500 | 1 | R | 500 x 20 | P | 958 | 20 | 3 | 2002 2008 2013 | 5.5 | 10 | 449 | 23.60 | 30 | -- | Blundo | FPY |
| AR | za | JUJ | R Zapla | -24.24 | -65.08 | 1610 | 1 | R | 500 x 20 | P | 852 | 15.1 | 2 | 2002 2012 | 10 | 10 | 584 | 30.91 | 20 | -- | Blundo | FPY |
| AR | za.a | JUJ | R Zapla | -24.23 | -65.06 | 2059 | 1 | R | 500 x 20 | P | 1143 | 12.6 | 2 | 2003 2012 | 9 | 10 | 537 | 22.27 | 14 | -- | Blundo | FPY |
| AR | ctj | TUC | Taficillo | -26.72 | -65.36 | 1640 | 0.24 | R | 60 x 40 | P | 1346 | 13.7 | 6 | 1991 1996 2001 2007 2011 2016 | 5 | 10 | 517 | 20.64 | 4 | 45 | Malizia | IER |
| AR | Mi1 | TUC | Taficillo | -26.70 | -65.34 | 1750 | 0.48 | R | 80 x 60 | P | 1591 | 13.2 | 6 | 1991 1996 2001 2007 2011 2016 | 5 | 10 | 504 | 36.07 | 9 | -- | Malizia | IER |
| AR | Mi2 | TUC | Taficillo | -26.71 | -65.34 | 1630 | 0.4 | I | -- | P | 1621 | 13.6 | 6 | 1991 1996 2001 2007 2011 2016 | 5 | 10 | 530 | 38.62 | 14 | -- | Malizia | IER |
| AR | Ppv2 | TUC | Taficillo | -26.72 | -65.35 | 1590 | 0.24 | R | 60 x 40 | P | 1553 | 13.3 | 6 | 1991 1996 2001 2007 2011 2016 | 5 | 10 | 329 | 30.55 | 7 | 425 | Malizia | IER |
| AR | Ppv1 | TUC | Taficillo | -26.71 | -65.35 | 1600 | 0.24 | R | 60 x 40 | P | 1527 | 13.3 | 6 | 1991 1996 2001 2007 2011 2016 | 5 | 10 | 725 | 47.15 | 7 | 420 | Malizia | IER |
| AR | Aaj | TUC | Taficillo | -26.70 | -65.34 | 1860 | 0.32 | I | -- | P | 1591 | 13.2 | 6 | 1991 1996 2001 2007 2011 2016 | 5 | 10 | 654 | 16.51 | 4 | 30 | Malizia | IER |
| AR | Ppj | TUC | Taficillo | -26.70 | -65.34 | 1830 | 0.16 | Q | 40 x 40 | P | 1591 | 13.2 | 6 | 1991 1996 2001 2007 2011 2016 | 5 | 10 | 681 | 32.40 | 3 | 210 | Malizia | IER |
| AR | Aav2 | TUC | Taficillo | -26.70 | -65.34 | 1780 | 0.24 | R | 60 x 40 | P | 1441 | 13.8 | 6 | 1991 1996 2001 2007 2011 2016 | 5 | 10 | 375 | 30.19 | 7 | 70 | Malizia | IER |
| AR | Ctv | TUC | Taficillo | -26.73 | -65.36 | 1610 | 0.24 | R | 60 x 40 | P | 1382 | 13.9 | 6 | 1991 1996 2001 2007 2011 2016 | 5 | 10 | 325 | 21.97 | 4 | 180 | Malizia | IER |
| AR | Ppi | TUC | Taficillo | -26.70 | -65.33 | 1700 | 0.24 | R | 60 x 40 | P | 1693 | 12.5 | 6 | 1991 1996 2001 2007 2011 2016 | 5 | 10 | 533 | 58.99 | 7 | 320 | Malizia | IER |
| AR | ceb | TUC | Cronosecuencia | -26.77 | -65.33 | 805 | 1 | I | -- | P | 1520 | 17.8 | 6 | 1991 1996 2001 2006 2011 2016 | 5 | 10 | 295 | 14.16 | 22 | 45-50 | Malizia | IER |
| AR | gua | TUC | Cronosecuencia | -26.77 | -65.32 | 741 | 0.96 | R | 160 x 60 | P | 1467 | 18.2 | 6 | 1991 1996 2001 2006 2011 2016 | 5 | 10 | 679 | 11.92 | 15 | 11-12 | Malizia | IER |
| AR | nor | TUC | Cronosecuencia | -26.76 | -65.33 | 910 | 0.96 | I | -- | P | 1529 | 17.2 | 6 | 1991 1996 2001 2006 2011 2016 | 5 | 10 | 328 | 26.61 | 21 | -- | Malizia | IER |
| AR | sur | TUC | Cronosecuencia | -26.76 | -65.33 | 886 | 1 | I | -- | P | 1529 | 17.2 | 6 | 1991 1996 2001 2006 2011 2016 | 5 | 10 | 447 | 35.55 | 23 | -- | Malizia | IER |
| AR | mor | TUC | Cronosecuencia | -26.77 | -65.32 | 740 | 1 | Q | 100 x 100 | P | 1467 | 18.2 | 6 | 1991 1996 2001 2006 2011 2016 | 5 | 10 | 466 | 16.45 | 20 | 25-30 | Malizia | IER |
| AR | nog | TUC | Cronosecuencia | -26.77 | -65.33 | 751 | 1 | I | -- | P | 1520 | 17.8 | 6 | 1991 1996 2001 2006 2011 2016 | 5 | 10 | 592 | 28.43 | 19 | 45-50 | Malizia | IER |
| AR | sup | TUC | Superplot | -26.76 | -65.33 | 1000 | 6 | R | 300 x 200 | P | 1529 | 17.2 | 6 | 1992 1997 2002 2007 2012 2017 | 5 | 10 \| 5* | 306 | 31.57 | 26 | -- | Malizia | IER |
| AR | Nativo | TUC | Horco Molle | -26.78 | -65.33 | 701 | 0.4 | I | -- | NP | 1506 | 18.0 | 1 | 2015 | -- | 10 | 588 | 34.68 | 17 | 30 | Ceballos | IER |
| AR | Cebilar | TUC | Horco Molle | -26.79 | -65.32 | 649 | 0.4 | I | -- | NP | 1392 | 19.1 | 1 | 2016 | -- | 10 | 605 | 35.92 | 18 | 30 | Ceballos | IER |
| AR | Sismógrafo | TUC | Horco Molle | -26.79 | -65.34 | 728 | 0.4 | I | -- | NP | 1567 | 17.2 | 1 | 2015 | -- | 10 | 585 | 27.6 | 21 | 60 | Ceballos | IER |
| AR | Frontino | TUC | Horco Molle | -26.80 | -65.34 | 669 | 0.4 | I | -- | NP | 1562 | 17.4 | 1 | 2016 | -- | 10 | 458 | 34.25 | 14 | 60 | Ceballos | IER |
| AR | Cuesta Vieja | TUC | Horco Molle | -26.78 | -65.33 | 731 | 0.4 | I | -- | NP | 1506 | 18.0 | 1 | 2016 | -- | 10 | 563 | 32.1 | 16 | -- | Ceballos | IER |
| BO | si | TAR | RN Tariquía | -22.25 | -64.56 | 1051 | 1 | R | 500 x 20 | P | 702 | 19.7 | 1 | 2003 | -- | 10 | 690 | 36.03 | 45 | -- | Blundo | FPY |
| BO | Chaqui.31 | LPZ | Madidi | -14.68 | -69.02 | 2872 | 1 | Q | 100x100 | P | 995 | 13.1 | 2 | 2008 2014 | 6.2 | 10 | 916 | 20.72 | 38 | NA | Tello | MBG |
| BO | Chaqui.32 | LPZ | Madidi | -14.69 | -69.03 | 3116 | 1 | Q | 100x100 | P | 1201 | 10.6 | 2 | 2008 2014 | 6.2 | 10 | 870 | 22.48 | 35 | NA | Tello | MBG |
| BO | Chiriu.2 | LPZ | Madidi | -14.50 | -68.25 | 1890 | 1 | Q | 100x100 | P | 1792 | 16.1 | 2 | 2002 2012 | 10.1 | 10 | 691 | 22.57 | 81 | NA | Tello | MBG |
| BO | Jucuma.35 | LPZ | Madidi | -14.77 | -68.99 | 2766 | 1 | Q | 100x100 | P | 770 | 14.4 | 2 | 2008 2013 | 5.1 | 10 | 623 | 17.14 | 35 | NA | Tello | MBG |
| BO | KaÃ±upa.44 | LPZ | ANMI Apolobamba | -14.89 | -68.86 | 3324 | 1 | Q | 100x100 | P | 1354 | 9 | 2 | 2010 2016 | 6.0 | 10 | 466 | 22.73 | 16 | NA | Tello | MBG |
| BO | Pintat.5 | LPZ | Madidi | -14.47 | -68.54 | 880 | 1 | Q | 100x100 | P | 1496 | 21 | 2 | 2003 2013 | 10.1 | 10 | 697 | 19.96 | 50 | NA | Tello | MBG |
| BO | Resina.12 | LPZ | Madidi | -14.33 | -68.57 | 662 | 1 | Q | 100x100 | P | 1104 | 23 | 2 | 2005 2014 | 8.8 | 10 | 734 | 20.69 | 50 | NA | Tello | MBG |
| BO | Resina.13 | LPZ | Madidi | -14.33 | -68.57 | 840 | 1 | Q | 100x100 | P | 1277 | 22.2 | 2 | 2005 2014 | 8.9 | 10 | 737 | 17.68 | 43 | NA | Tello | MBG |
| BO | Resina.14 | LPZ | Madidi | -14.33 | -68.57 | 1020 | 1 | Q | 100x100 | P | 1277 | 22.2 | 2 | 2005 2014 | 8.8 | 10 | 853 | 20.22 | 60 | NA | Tello | MBG |
| BO | Sanmar.21 | LPZ | Madidi | -14.16 | -68.72 | 1100 | 1 | Q | 100x100 | P | 1337 | 19.9 | 2 | 2006 2013 | 7.0 | 10 | 643 | 37.14 | 89 | NA | Tello | MBG |
| BO | Sanmar.22 | LPZ | Madidi | -14.15 | -68.75 | 1250 | 1 | Q | 100x100 | P | 1532 | 19.5 | 2 | 2006 2013 | 7.1 | 10 | 506 | 28.04 | 90 | NA | Tello | MBG |
| BO | Sumpul.33 | LPZ | Madidi | -14.58 | -68.78 | 1005 | 1 | Q | 100x100 | P | 773 | 21.1 | 2 | 2008 2014 | 6.2 | 10 | 617 | 29.32 | 71 | NA | Tello | MBG |
| BO | Sumpul.34 | LPZ | Madidi | -14.57 | -68.78 | 1223 | 1 | Q | 100x100 | P | 762 | 19.7 | 2 | 2008 2014 | 6.1 | 10 | 758 | 29.35 | 94 | NA | Tello | MBG |
| BO | Tapuri.45 | LPZ | ANMI Apolobamba | -15.21 | -68.76 | 2697 | 1 | Q | 100x100 | P | 675 | 15.1 | 2 | 2010 2015 | 5.0 | 10 | 708 | 31.77 | 42 | NA | Tello | MBG |
| BO | Tapuri.46 | LPZ | ANMI Apolobamba | -15.20 | -68.76 | 2369 | 1 | Q | 100x100 | P | 735 | 15.6 | 2 | 2010 2015 | 5.0 | 10 | 1099 | 20.21 | 42 | NA | Tello | MBG |
| BO | Terraz.41 | LPZ | ANMI Apolobamba | -15.11 | -68.92 | 2889 | 1 | Q | 100x100 | P | 644 | 11.7 | 2 | 2010 2015 | 6.2 | 10 | 825 | 20.52 | 32 | NA | Tello | MBG |
| BO | Tintay.24 | LPZ | Madidi | -14.78 | -68.59 | 1400 | 1 | Q | 100x100 | P | 904 | 18.6 | 2 | 2006 2012 | 5.9 | 10 | 728 | 21.28 | 113 | NA | Tello | MBG |
| BO | Tintay.25 | LPZ | Madidi | -14.79 | -68.58 | 1468 | 1 | Q | 100x100 | P | 869 | 19 | 2 | 2006 2012 | 5.9 | 10 | 685 | 27.21 | 91 | NA | Tello | MBG |
| BO | Titiri.42 | LPZ | Madidi | -15.13 | -68.88 | 2859 | 1 | Q | 100x100 | P | 660 | 11.3 | 2 | 2010 2015 | 5.2 | 10 | 652 | 24.42 | 34 | NA | Tello | MBG |
| BO | Tocoaq.28 | LPZ | Madidi | -14.61 | -68.95 | 2200 | 1 | Q | 100x100 | P | 1319 | 15.5 | 2 | 2007 2016 | 8.4 | 10 | 716 | 22.55 | 79 | NA | Tello | MBG |
| BO | Tocoaq.29 | LPZ | Madidi | -14.62 | -68.95 | 2400 | 1 | Q | 100x100 | P | 1358 | 14.3 | 2 | 2007 2016 | 8.5 | 10 | 767 | 21.49 | 81 | NA | Tello | MBG |
| BO | Tocoaq.30 | LPZ | Madidi | -14.63 | -68.96 | 2510 | 1 | Q | 100x100 | P | 1373 | 14.7 | 2 | 2007 2016 | 8.4 | 10 | 752 | 22.23 | 60 | NA | Tello | MBG |
| BO | Waturu.43 | LPZ | ANMI Apolobamba | -14.89 | -68.85 | 3035 | 1 | Q | 100x100 | P | 1464 | 9.9 | 2 | 2010 2016 | 6.0 | 10 | 627 | 25.37 | 23 | NA | Tello | MBG |
| BO | Yarimi.10 | LPZ | Madidi | -14.54 | -68.69 | 1000 | 1 | Q | 100x100 | P | 940 | 21.1 | 2 | 2005 2012 | 7.1 | 10 | 614 | 23.07 | 48 | NA | Tello | MBG |
| BO | Yarimi.11 | LPZ | Madidi | -14.55 | -68.69 | 1180 | 1 | Q | 100x100 | P | 953 | 21.1 | 2 | 2005 2012 | 7.1 | 10 | 604 | 29.82 | 56 | NA | Tello | MBG |
| BO | Yarimi.9 | LPZ | Madidi | -14.55 | -68.69 | 850 | 1 | Q | 100x100 | P | 862 | 21.9 | 2 | 2005 2012 | 7.2 | 10 | 430 | 28.81 | 41 | NA | Tello | MBG |
| PE | Chochos | SMA | PN Río Abiseo | -7.64 | -77.48 | 3450 | 1 | Q | 100 x 100 | P | 1209 | 10.2 | 1 | 1998 | -- | 10 | 669 | 36.59 | 28 | 50 | Young | UT. Austin |
| PE | PEPU.BORA. 01 | PIU | Bosque de Ramos | -4.71 | -79.46 | 2909 | 0.36 | Q | 60 x 60 | P | 952 | 10.6 | 2 | 2015 2017 | 2 | 10 | 1064 | 35.75 | 21 | 15 | Cuesta | ECOANDES |
| PE | PEPU.BORA. 03 | PIU | Bosque de Ramos | -4.71 | -79.46 | 2955 | 0.36 | Q | 60 x 60 | P | 952 | 10.6 | 2 | 2015 2017 | 2 | 10 | 1036 | 45.56 | 24 | 15 | Cuesta | ECOANDES |
| PE | PEPU.BORA. 04 | PIU | Bosque de Ramos | -4.70 | -79.46 | 2817 | 0.36 | Q | 60 x 60 | P | 903 | 12.5 | 2 | 2015 2017 | 2 | 10 | 1186 | 46.85 | 21 | 15 | Cuesta | ECOANDES |
| PE | PEPU.BORA. 02 | PIU | Bosque de Ramos | -4.70 | -79.46 | 2840 | 0.36 | Q | 60 x 60 | P | 903 | 12.5 | 2 | 2015 2017 | 2 | 10 | 875 | 37.14 | 23 | 15 | Cuesta | ECOANDES |
| PE | PEPU.BORA. 05 | PIU | Bosque de Ramos | -4.71 | -79.46 | 3049 | 0.36 | Q | 60 x 60 | P | 952 | 10.6 | 2 | 2015 2017 | 2 | 10 | 1114 | 65.91 | 18 | 15 | Cuesta | ECOANDES |
| PE | PEPU.BORA. 06 | PIU | Bosque de Ramos | -4.71 | -79.46 | 3096 | 0.36 | Q | 60 x 60 | P | 952 | 10.6 | 2 | 2015 2017 | 2 | 10 | 1236 | 51.94 | 21 | 15 | Cuesta | ECOANDES |
| PE | TRU-01 | CUS | Trocha Unión | -13.11 | -71.61 | 3450 | 1 | Q | 100 X 100 | P | 1500 | 10.1 | 1 | 2007 | -- | 10 | 637 | 30.21 | 32 | NA | Silman | ABERG |
| PE | TRU-02 | CUS | Trocha Unión | -13.11 | -71.61 | 3250 | 1 | Q | 100 X 100 | P | 1500 | 10.1 | 1 | 2007 | -- | 10 | 758 | 33.09 | 42 | NA | Silman | ABERG |
| PE | TRU-03 | CUS | Trocha Unión | -13.11 | -71.60 | 3000 | 1 | Q | 100 X 100 | P | 1441 | 11.7 | 1 | 2007 | -- | 10 | 567 | 19.90 | 33 | NA | Silman | ABERG |
| PE | TRU-04 | CUS | Trocha Unión | -13.11 | -71.59 | 2750 | 1 | Q | 100 X 100 | P | 1364 | 13.1 | 1 | 2007 | -- | 10 | 873 | 31.58 | 53 | NA | Silman | ABERG |
| PE | TRU-05 | CUS | Trocha Unión | -13.09 | -71.57 | 2500 | 1 | Q | 100 X 100 | P | 1465 | 14.4 | 1 | 2007 | -- | 10 | 1063 | 45.85 | 55 | NA | Silman | ABERG |
| PE | TRU-06 | CUS | Trocha Unión | -13.08 | -71.57 | 2250 | 1 | Q | 100 X 100 | P | 1389 | 16.5 | 1 | 2007 | -- | 10 | 1041 | 39.14 | 75 | NA | Silman | ABERG |
| PE | TRU-07 | CUS | Trocha Unión | -13.07 | -71.56 | 2000 | 1 | Q | 100 X 100 | P | 1231 | 17.7 | 1 | 2007 | -- | 10 | 996 | 23.68 | 73 | NA | Silman | ABERG |
| PE | TRU-08 | CUS | Trocha Unión | -13.07 | -71.56 | 1800 | 1 | Q | 100 X 100 | P | 1314 | 18 | 1 | 2007 | -- | 10 | 1262 | 31.94 | 107 | NA | Silman | ABERG |
| PE | WAY-01 | CUS | Trocha Unión | -13.19 | -71.59 | 3000 | 1 | Q | 100 X 100 | P | 1234 | 11.3 | 1 | 2007 | -- | 10 | 1107 | 31.40 | 50 | NA | Silman | ABERG |
| PE | Abiseo.A1 | SMA | PN Río Abiseo | -7.43 | -76.90 | 898 | 0.1 | R | 50 x 20 | NP | 1802 | 22.7 | 1 | 2017 | -- | 2.5 | 640 | 35.44 | 34 | -- | Cayuela | URJC |
| PE | Abiseo.A2 | SMA | PN Río Abiseo | -7.43 | -76.90 | 819 | 0.1 | R | 50 x 20 | NP | 1618 | 23.7 | 1 | 2017 | -- | 2.5 | 460 | 53.58 | 25 | -- | Cayuela | URJC |
| PE | Abiseo.A3 | SMA | PN Río Abiseo | -7.42 | -76.90 | 941 | 0.1 | R | 50 x 20 | NP | 1867 | 23.5 | 1 | 2017 | -- | 2.5 | 730 | 36.15 | 41 | -- | Cayuela | URJC |
| PE | Abiseo.A4 | SMA | PN Río Abiseo | -7.42 | -76.90 | 963 | 0.1 | R | 50 x 20 | NP | 1867 | 23.5 | 1 | 2017 | -- | 2.5 | 780 | 33.47 | 41 | -- | Cayuela | URJC |
| PE | Abiseo.A5 | SMA | PN Río Abiseo | -7.42 | -76.90 | 1122 | 0.1 | R | 50 x 20 | NP | 2007 | 22.1 | 1 | 2017 | -- | 2.5 | 870 | 59.25 | 49 | -- | Cayuela | URJC |
| PE | Abiseo.A6 | SMA | PN Río Abiseo | -7.43 | -76.90 | 780 | 0.1 | R | 50 x 20 | NP | 1618 | 23.7 | 1 | 2017 | -- | 2.5 | 810 | 36.06 | 53 | -- | Cayuela | URJC |
| PE | Abiseo.A7 | SMA | PN Río Abiseo | -7.43 | -76.90 | 912 | 0.1 | R | 50 x 20 | NP | 1802 | 22.7 | 1 | 2017 | -- | 2.5 | 560 | 38.80 | 36 | -- | Cayuela | URJC |
| PE | Abiseo.A8 | SMA | PN Río Abiseo | -7.43 | -76.90 | 793 | 0.1 | R | 50 x 20 | NP | 1802 | 22.7 | 1 | 2017 | -- | 2.5 | 650 | 34.13 | 40 | -- | Cayuela | URJC |
| PE | Abiseo.A9 | SMA | PN Río Abiseo | -7.43 | -76.90 | 745 | 0.1 | R | 50 x 20 | NP | 1618 | 23.7 | 1 | 2017 | -- | 2.5 | 610 | 62.15 | 37 | -- | Cayuela | URJC |
| PE | Abiseo.A10 | SMA | PN Río Abiseo | -7.45 | -76.93 | 841 | 0.1 | R | 50 x 20 | NP | 1325 | 25.1 | 1 | 2017 | -- | 2.5 | 760 | 40.49 | 45 | -- | Cayuela | URJC |
| PE | Abiseo.B1 | SMA | PN Río Abiseo | -7.64 | -77.38 | 2117 | 0.1 | R | 50 x 20 | NP | 1058 | 16.1 | 1 | 2016 | -- | 2.5 | 740 | 54.32 | 26 | -- | Cayuela | URJC |
| PE | Abiseo.B2 | SMA | PN Río Abiseo | -7.64 | -77.38 | 2188 | 0.1 | R | 50 x 20 | NP | 1058 | 16.1 | 1 | 2016 | -- | 2.5 | 1270 | 40.84 | 33 | -- | Cayuela | URJC |
| PE | Abiseo.B3 | SMA | PN Río Abiseo | -7.64 | -77.38 | 2152 | 0.1 | R | 50 x 20 | NP | 1058 | 16.1 | 1 | 2016 | -- | 2.5 | 1080 | 45.11 | 28 | -- | Cayuela | URJC |
| PE | Abiseo.B4 | SMA | PN Río Abiseo | -7.63 | -77.38 | 2093 | 0.1 | R | 50 x 20 | NP | 1108 | 15.2 | 1 | 2016 | -- | 2.5 | 880 | 63.30 | 31 | -- | Cayuela | URJC |
| PE | Abiseo.B5 | SMA | PN Río Abiseo | -7.63 | -77.37 | 2144 | 0.1 | R | 50 x 20 | NP | 1079 | 16.6 | 1 | 2016 | -- | 2.5 | 470 | 38.43 | 18 | -- | Cayuela | URJC |
| PE | Abiseo.B6 | SMA | PN Río Abiseo | -7.64 | -77.38 | 2143 | 0.1 | R | 50 x 20 | NP | 1104 | 14.7 | 1 | 2016 | -- | 2.5 | 820 | 30.18 | 26 | -- | Cayuela | URJC |
| PE | Abiseo.B7 | SMA | PN Río Abiseo | -7.64 | -77.39 | 2189 | 0.1 | R | 50 x 20 | NP | 1104 | 14.7 | 1 | 2016 | -- | 2.5 | 690 | 38.76 | 24 | -- | Cayuela | URJC |
| PE | Abiseo.B8 | SMA | PN Río Abiseo | -7.64 | -77.39 | 2233 | 0.1 | R | 50 x 20 | NP | 1104 | 14.7 | 1 | 2016 | -- | 2.5 | 520 | 28.27 | 16 | -- | Cayuela | URJC |
| PE | Abiseo.B9 | SMA | PN Río Abiseo | -7.64 | -77.39 | 2222 | 0.1 | R | 50 x 20 | NP | 1104 | 14.7 | 1 | 2016 | -- | 2.5 | 660 | 51.07 | 25 | -- | Cayuela | URJC |
| PE | Abiseo.B10 | SMA | PN Río Abiseo | -7.64 | -77.39 | 2101 | 0.1 | R | 50 x 20 | NP | 1149 | 13.1 | 1 | 2016 | -- | 2.5 | 640 | 31.21 | 20 | -- | Cayuela | URJC |
| PE | Abiseo.C1 | SMA | PN Río Abiseo | -7.66 | -77.43 | 2721 | 0.1 | R | 50 x 20 | NP | 1372 | 12.8 | 1 | 2016 | -- | 2.5 | 680 | 23.09 | 19 | -- | Cayuela | URJC |
| PE | Abiseo.C2 | SMA | PN Río Abiseo | -7.66 | -77.43 | 2767 | 0.1 | R | 50 x 20 | NP | 1375 | 12.8 | 1 | 2016 | -- | 2.5 | 610 | 37.45 | 14 | -- | Cayuela | URJC |
| PE | Abiseo.C3 | SMA | PN Río Abiseo | -7.66 | -77.42 | 2791 | 0.1 | R | 50 x 20 | NP | 1393 | 12.9 | 1 | 2016 | -- | 2.5 | 1240 | 45.96 | 19 | -- | Cayuela | URJC |
| PE | Abiseo.C4 | SMA | PN Río Abiseo | -7.66 | -77.44 | 2759 | 0.1 | R | 50 x 20 | NP | 1372 | 12.8 | 1 | 2016 | -- | 2.5 | 520 | 29.67 | 20 | -- | Cayuela | URJC |
| PE | Abiseo.C5 | SMA | PN Río Abiseo | -7.66 | -77.44 | 2778 | 0.1 | R | 50 x 20 | NP | 1340 | 12.4 | 1 | 2016 | -- | 2.5 | 330 | 31.26 | 13 | -- | Cayuela | URJC |
| PE | Abiseo.C6 | SMA | PN Río Abiseo | -7.66 | -77.45 | 2866 | 0.1 | R | 50 x 20 | NP | 1340 | 12.4 | 1 | 2016 | -- | 2.5 | 980 | 38.55 | 20 | -- | Cayuela | URJC |
| PE | Abiseo.C7 | SMA | PN Río Abiseo | -7.67 | -77.45 | 2980 | 0.1 | R | 50 x 20 | NP | 1418 | 11.6 | 1 | 2016 | -- | 2.5 | 1180 | 57.68 | 20 | -- | Cayuela | URJC |
| PE | Abiseo.C8 | SMA | PN Río Abiseo | -7.66 | -77.43 | 2775 | 0.1 | R | 50 x 20 | NP | 1375 | 12.8 | 1 | 2016 | -- | 2.5 | 1000 | 49.80 | 26 | -- | Cayuela | URJC |
| PE | Abiseo.C9 | SMA | PN Río Abiseo | -7.66 | -77.43 | 2821 | 0.1 | R | 50 x 20 | NP | 1375 | 12.8 | 1 | 2016 | -- | 2.5 | 1020 | 30.61 | 20 | -- | Cayuela | URJC |
| PE | Abiseo.C10 | SMA | PN Río Abiseo | -7.66 | -77.43 | 2810 | 0.1 | R | 50 x 20 | NP | 1375 | 12.8 | 1 | 2016 | -- | 2.5 | 810 | 28.49 | 24 | -- | Cayuela | URJC |
| EC | ECCA.VINE.01 | CAR | Virgen Negra | 0.66 | -77.64 | 3352 | 0.36 | Q | 60 x 60 | P | 1237 | 9.3 | 2 | 2015 2017 | 2 | 5 | 594 | 30.16 | 19 | 20 | Cuesta | ECOANDES |
| EC | ECCA.VINE.02 | CAR | Virgen Negra | 0.66 | -77.64 | 3428 | 0.36 | Q | 60 x 60 | P | 1237 | 9.3 | 2 | 2015 2017 | 2 | 5 | 736 | 31.11 | 14 | 20 | Cuesta | ECOANDES |
| EC | ECCA.GUAN.01 | CAR | Guandera | 0.59 | -77.70 | 3511 | 0.36 | Q | 60 x 60 | P | 840 | 9 | 2 | 2015 2017 | 2 | 5 | 525 | 29.91 | 14 | 20 | Cuesta | ECOANDES |
| EC | ECCA.GUAN.02 | CAR | Guandera | 0.59 | -77.70 | 3410 | 0.36 | Q | 60 x 60 | P | 840 | 9 | 2 | 2015 2017 | 2 | 5 | 925 | 52.98 | 10 | 20 | Cuesta | ECOANDES |
| EC | ECCA.PAGR.01 | CAR | Palmar Grande | 0.36 | -77.84 | 3037 | 0.36 | Q | 60 x 60 | P | 839 | 9.1 | 2 | 2015 2017 | 2 | 5 | 1006 | 25.50 | 25 | 15 | Cuesta | ECOANDES |
| EC | ECPI.MAPI.01 | PIC | Mashpishungo | 0.19 | -78.91 | 653 | 0.36 | Q | 60 x 60 | P | 2111 | 22.9 | 2 | 2015 2017 | 2 | 5 | 508 | 19.90 | 38 | 25 | Cuesta | ECOANDES-PBA |
| EC | ECPI.MAPI.02 | PIC | Mashpishungo | 0.19 | -78.91 | 632 | 0.36 | Q | 60 x 60 | P | 2111 | 22.9 | 2 | 2015 2017 | 2 | 5 | 517 | 18.99 | 54 | 25 | Cuesta | ECOANDES-PBA |
| EC | ECPI.MALO.02 | PIC | MashpiLodge | 0.16 | -78.88 | 827 | 0.36 | Q | 60 x 60 | P | 2448 | 21.4 | 2 | 2015 2017 | 2 | 5 | 506 | 28.75 | 63 | 25 | Cuesta | ECOANDES-PBA |
| EC | ECPI.MALO.01 | PIC | MashpiLodge | 0.17 | -78.88 | 1018 | 0.36 | Q | 60 x 60 | P | 2431 | 21.5 | 2 | 2015 2017 | 2 | 5 | 608 | 21.95 | 65 | 25 | Cuesta | ECOANDES-PBA |
| EC | ECPI.CEDR.01 | PIC | El Cedral | 0.16 | -78.88 | 2492 | 0.36 | Q | 60 x 60 | P | 2431 | 21.5 | 2 | 2015 2017 | 2 | 5 | 647 | 27.31 | 38 | 25 | Cuesta | ECOANDES-PBA |
| EC | ECPI.CEDR.03 | PIC | El Cedral | 0.11 | -78.57 | 2212 | 0.36 | Q | 60 x 60 | P | 1644 | 14.7 | 2 | 2015 2017 | 2 | 5 | 511 | 28.33 | 40 | 25 | Cuesta | ECOANDES-PBA |
| EC | ECPI.INTI.01 | PIC | Intyllacta | 0.05 | -78.72 | 1879 | 0.36 | Q | 60 x 60 | P | 1939 | 16.6 | 2 | 2015 2017 | 2 | 5 | 500 | 25.03 | 40 | 25 | Cuesta | ECOANDES-PBA |
| EC | ECPI.INTI.02 | PIC | Intyllacta | 0.05 | -78.72 | 1829 | 0.36 | Q | 60 x 60 | P | 1939 | 16.6 | 2 | 2015 2017 | 2 | 5 | 489 | 19.73 | 42 | 25 | Cuesta | ECOANDES-PBA |
| EC | ECPI.BECL.02 | PIC | Bellavista | -0.01 | -78.69 | 2282 | 0.36 | Q | 60 x 60 | P | 1993 | 14.7 | 2 | 2015 2017 | 2 | 5 | 303 | 17.00 | 34 | 25 | Cuesta | ECOANDES-PBA |
| EC | ECPI.BECL.03 | PIC | Bellavista | -0.01 | -78.69 | 2203 | 0.36 | Q | 60 x 60 | P | 1993 | 14.7 | 2 | 2015 2017 | 2 | 5 | 411 | 16.66 | 24 | 25 | Cuesta | ECOANDES-PBA |
| EC | ECPI.MIND.01 | PIC | Urcullacta | -0.03 | -78.81 | 1277 | 0.36 | Q | 60 x 60 | P | 1769 | 20 | 2 | 2015 2017 | 2 | 5 | 519 | 22.95 | 46 | 25 | Cuesta | ECOANDES-PBA |
| EC | ECPI.BECL.01 | PIC | Bellavista | -0.02 | -78.69 | 2313 | 0.36 | Q | 60 x 60 | P | 2033 | 14.3 | 2 | 2015 2017 | 2 | 5 | 658 | 18.61 | 30 | 25 | Cuesta | ECOANDES-PBA |
| EC | ECPI.RIBR.01 | PIC | Río Bravo | -0.08 | -78.74 | 1640 | 0.36 | Q | 60 x 60 | P | 1920 | 18.4 | 2 | 2015 2017 | 2 | 5 | 831 | 19.70 | 56 | 25 | Cuesta | ECOANDES-PBA |
| EC | ECPI.VERD.02 | PIC | Verdecocha | -0.10 | -78.60 | 2932 | 0.36 | Q | 60 x 60 | P | 1684 | 11.5 | 2 | 2015 2017 | 2 | 5 | 617 | 18.30 | 25 | 25 | Cuesta | ECOANDES-PBA |
| EC | ECPI.VERD.03 | PIC | Verdecocha | -0.10 | -78.60 | 3109 | 0.36 | Q | 60 x 60 | P | 1684 | 11.5 | 2 | 2015 2017 | 2 | 5 | 589 | 21.42 | 32 | 25 | Cuesta | ECOANDES-PBA |
| EC | ECPI.VERD.01 | PIC | Verdecocha | -0.12 | -78.60 | 3421 | 0.36 | Q | 60 x 60 | P | 1605 | 9.3 | 2 | 2015 2017 | 2 | 5 | 914 | 25.42 | 30 | 25 | Cuesta | ECOANDES-PBA |
| EC | ECPI.YANA.01 | PIC | Yanacocha | -0.13 | -78.59 | 3507 | 0.36 | Q | 60 x 60 | P | 1580 | 7.3 | 2 | 2015 2017 | 2 | 5 | 403 | 24.25 | 15 | 25 | Cuesta | ECOANDES-PBA |
| EC | Ahuano.01 | NAP | Ahuano | -1.07 | -77.49 | 439 | 0.36 | Q | 60 x 60 | P | 4415 | 24.1 | 1 | 2012 | -- | 10 | 150 | 16.79 | 31 | -- | Cuesta | CONDESAN |
| EC | Ahuano.02 | NAP | Ahuano | -1.07 | -77.50 | 473 | 0.36 | Q | 60 x 60 | P | 4415 | 24.1 | 1 | 2012 | -- | 10 | 261 | 25.37 | 42 | -- | Cuesta | CONDESAN |
| EC | Ahuano.03 | NAP | Ahuano | -1.06 | -77.50 | 407 | 0.36 | Q | 60 x 60 | P | 4157 | 24.3 | 1 | 2012 | -- | 10 | 189 | 19.97 | 37 | -- | Cuesta | CONDESAN |
| EC | Mushullacta.01 | NAP | Mushullacta | -0.82 | -77.59 | 983 | 0.36 | Q | 60 x 60 | P | 3620 | 21.1 | 1 | 2012 | -- | 10 | 219 | 20.77 | 36 | -- | Cuesta | CONDESAN |
| EC | Mushullacta.02 | NAP | Mushullacta | -0.81 | -77.59 | 975 | 0.36 | R | 60 x 60 | P | 3652 | 21.5 | 1 | 2012 | -- | 10 | 275 | 21.35 | 53 | -- | Cuesta | CONDESAN |
| EC | Mushullacta.03 | NAP | Mushullacta | -0.82 | -77.58 | 1000 | 0.36 | R | 60 x 60 | P | 3764 | 20.6 | 1 | 2012 | -- | 10 | 247 | 22.52 | 49 | -- | Cuesta | CONDESAN |
| EC | SV.Guayusa. Yacu.01 | NAP | San Vicente | -0.90 | -77.24 | 360 | 0.36 | Q | 60 x 60 | P | 4362 | 24.4 | 1 | 2013 | -- | 10 | 203 | 17.52 | 32 | -- | Cuesta | CONDESAN |
| EC | SV.Guayusa. Yacu.02 | NAP | San Vicente | -0.90 | -77.24 | 376 | 0.36 | Q | 60 x 60 | P | 4362 | 24.4 | 1 | 2013 | -- | 10 | 283 | 24.67 | 39 | -- | Cuesta | CONDESAN |
| EC | SV.Guayusa. Yacu.03 | NAP | San Vicente | -0.90 | -77.24 | 381 | 0.36 | Q | 60 x 60 | P | 4362 | 24.4 | 1 | 2013 | -- | 10 | 239 | 16.74 | 39 | -- | Cuesta | CONDESAN |
| EC | Venecia.01 | NAP | Venecia | -1.06 | -77.68 | 498 | 0.36 | Q | 60 x 60 | P | 4804 | 24.2 | 1 | 2013 | -- | 10 | 233 | 22.38 | 45 | -- | Cuesta | CONDESAN |
| EC | Venecia.02 | NAP | Venecia | -1.06 | -77.68 | 488 | 0.36 | Q | 60 x 60 | P | 4804 | 24.2 | 1 | 2013 | -- | 10 | 133 | 9.95 | 25 | -- | Cuesta | CONDESAN |
| EC | Venecia.03 | NAP | Venecia | -1.05 | -77.68 | 458 | 0.36 | Q | 60 x 60 | P | 4302 | 24.3 | 1 | 2013 | -- | 10 | 183 | 15.27 | 41 | -- | Cuesta | CONDESAN |
| EC | Wachi.Yacu. Chico.01 | NAP | Wachi | -0.92 | -77.27 | 441 | 0.36 | Q | 60 x 60 | P | 4464 | 24.2 | 1 | 2012 | -- | 10 | 194 | 4.75 | 34 | -- | Cuesta | CONDESAN |
| EC | Wachi.Yacu. Chico.02 | NAP | Wachi | -0.92 | -77.28 | 414 | 0.36 | Q | 60 x 60 | P | 4292 | 24.3 | 1 | 2012 | -- | 10 | 214 | 13.52 | 31 | -- | Cuesta | CONDESAN |
| EC | Wachi.Yacu. Chico.03 | NAP | Wachi | -0.92 | -77.28 | 396 | 0.36 | Q | 60 x 60 | P | 4292 | 24.3 | 1 | 2012 | -- | 10 | 272 | 22.55 | 37 | -- | Cuesta | CONDESAN |
| EC | Wamani.01 | NAP | Wamani | -0.73 | -77.63 | 1144 | 0.08 | R | 20 x 40 | P | 3693 | 20.5 | 1 | 2013 | -- | 10 | 488 | 22.04 | 11 | -- | Cuesta | CONDESAN |
| EC | Wamani.02 | NAP | Wamani | -0.70 | -77.61 | 1249 | 0.08 | R | 20 x 40 | P | 3635 | 20.2 | 1 | 2013 | -- | 10 | 563 | 37.77 | 29 | -- | Cuesta | CONDESAN |
| EC | Wamani.03 | NAP | Wamani | -0.75 | -77.66 | 1123 | 0.08 | R | 20 x 40 | P | 3747 | 20.6 | 1 | 2013 | -- | 10 | 425 | 28.03 | 16 | -- | Cuesta | CONDESAN |
| EC | Wamani.04 | NAP | Wamani | -0.75 | -77.65 | 1057 | 0.08 | R | 20 x 40 | P | 3765 | 20.8 | 1 | 2013 | -- | 10 | 475 | 35.80 | 19 | -- | Cuesta | CONDESAN |
| EC | Wamani.05 | NAP | Wamani | -0.75 | -77.65 | 1054 | 0.08 | R | 20 x 40 | P | 3765 | 20.8 | 1 | 2013 | -- | 10 | 450 | 31.11 | 16 | -- | Cuesta | CONDESAN |
| EC | Wamani.06 | NAP | Wamani | -0.75 | -77.65 | 1071 | 0.08 | R | 20 x 40 | P | 3765 | 20.8 | 1 | 2013 | -- | 10 | 513 | 42.45 | 20 | -- | Cuesta | CONDESAN |
| EC | Wamani.07 | NAP | Wamani | -0.75 | -77.64 | 1089 | 0.08 | R | 20 x 40 | P | 3694 | 20.9 | 1 | 2013 | -- | 10 | 500 | 38.75 | 10 | -- | Cuesta | CONDESAN |
| EC | Wamani.08 | NAP | Wamani | -0.73 | -77.63 | 1135 | 0.08 | R | 20 x 40 | P | 3693 | 20.5 | 1 | 2013 | -- | 10 | 488 | 15.83 | 10 | -- | Cuesta | CONDESAN |
| EC | Wamani.09 | NAP | Wamani | -0.73 | -77.63 | 1158 | 0.08 | R | 20 x 40 | P | 3693 | 20.5 | 1 | 2013 | -- | 10 | 638 | 26.82 | 23 | -- | Cuesta | CONDESAN |
| EC | Wamani.10 | NAP | Wamani | -0.73 | -77.64 | 1149 | 0.08 | R | 20 x 40 | P | 3668 | 20.4 | 1 | 2013 | -- | 10 | 813 | 23.70 | 23 | -- | Cuesta | CONDESAN |
| EC | Wamani.11 | NAP | Wamani | -0.70 | -77.63 | 1400 | 0.08 | R | 20 x 40 | P | 3744 | 19.5 | 1 | 2013 | -- | 10 | 475 | 44.70 | 22 | -- | Cuesta | CONDESAN |
| EC | Wamani.12 | NAP | Wamani | -0.69 | -77.63 | 1314 | 0.08 | R | 20 x 40 | P | 3567 | 19.8 | 1 | 2013 | -- | 10 | 375 | 44.76 | 14 | -- | Cuesta | CONDESAN |
| EC | Wamani.13 | NAP | Wamani | -0.69 | -77.62 | 1301 | 0.08 | R | 20 x 40 | P | 3567 | 19.8 | 1 | 2013 | -- | 10 | 575 | 34.74 | 23 | -- | Cuesta | CONDESAN |
| EC | Wamani.14 | NAP | Wamani | -0.74 | -77.64 | 1127 | 0.08 | R | 20 x 40 | P | 3674 | 20.7 | 1 | 2013 | -- | 10 | 513 | 32.70 | 30 | -- | Cuesta | CONDESAN |
| EC | Wamani.15 | NAP | Wamani | -0.69 | -77.62 | 1316 | 0.08 | R | 20 x 40 | P | 3567 | 19.8 | 1 | 2013 | -- | 1 | 488 | 35.04 | 27 | -- | Cuesta | CONDESAN |
| EC | Wamani.16 | NAP | Wamani | -0.73 | -77.61 | 1130 | 0.08 | R | 20 x 40 | P | 4106 | 20.5 | 1 | 2013 | -- | 10 | 350 | 29.32 | 21 | -- | Cuesta | CONDESAN |
| EC | Wamani.17 | NAP | Wamani | -0.74 | -77.59 | 1074 | 0.08 | R | 20 x 40 | P | 4162 | 21 | 1 | 2013 | -- | 10 | 638 | 31.49 | 31 | -- | Cuesta | CONDESAN |
| EC | Wamani.18 | NAP | Wamani | -0.73 | -77.61 | 1166 | 0.08 | R | 20 x 40 | P | 4174 | 20.5 | 1 | 2013 | -- | 10 | 625 | 70.18 | 28 | -- | Cuesta | CONDESAN |
| EC | Wamani.19 | NAP | Wamani | -0.71 | -77.63 | 1200 | 0.08 | R | 20 x 40 | P | 3653 | 20.2 | 1 | 2013 | -- | 1 | 475 | 21.68 | 11 | -- | Cuesta | CONDESAN |
| EC | Wamani.20 | NAP | Wamani | -0.70 | -77.63 | 1232 | 0.08 | R | 20 x 40 | P | 3506 | 20 | 1 | 2013 | -- | 10 | 400 | 19.33 | 16 | -- | Cuesta | CONDESAN |
| EC | Yanacocha.01 | PIC | Yanacocha | -0.13 | -78.59 | 3790 | 0.01 | Q | 10 x 10 | P | 1580 | 7.3 | 1 | 2012 | -- | 10 | 800 | 10.35 | 2 | 22 | Cuesta | CONDESAN |
| EC | Yanacocha.02 | PIC | Yanacocha | -0.13 | -78.59 | 3857 | 0.01 | Q | 10 x 10 | P | 1580 | 7.3 | 1 | 2012 | -- | 10 | 100 | 1.81 | 1 | 22 | Cuesta | CONDESAN |
| EC | Yanacocha.03 | PIC | Yanacocha | -0.13 | -78.58 | 3891 | 0.01 | Q | 10 x 10 | P | 1580 | 7.3 | 1 | 2012 | -- | 10 | 1900 | 43.95 | 3 | 22 | Cuesta | CONDESAN |
| EC | Yanacocha.04 | PIC | Yanacocha | -0.13 | -78.58 | 3894 | 0.01 | Q | 10 x 10 | P | 1580 | 7.3 | 1 | 2012 | -- | 10 | 1200 | 28.17 | 6 | 22 | Cuesta | CONDESAN |
| EC | Yanacocha.05 | PIC | Yanacocha | -0.13 | -78.59 | 3798 | 0.01 | Q | 10 x 10 | P | 1580 | 7.3 | 1 | 2012 | -- | 10 | 1700 | 43.75 | 2 | 22 | Cuesta | CONDESAN |
| EC | Yanacocha.06 | PIC | Yanacocha | -0.13 | -78.59 | 3836 | 0.01 | Q | 10 x 10 | P | 1580 | 7.3 | 1 | 2012 | -- | 10 | 1000 | 31.74 | 3 | 22 | Cuesta | CONDESAN |
| EC | Yanacocha.07 | PIC | Yanacocha | -0.13 | -78.59 | 3821 | 0.01 | Q | 10 x 10 | P | 1580 | 7.3 | 1 | 2012 | -- | 10 | 2200 | 47.05 | 4 | 22 | Cuesta | CONDESAN |
| EC | Yanacocha.08 | PIC | Yanacocha | -0.13 | -78.59 | 3820 | 0.01 | Q | 10 x 10 | P | 1580 | 7.3 | 1 | 2012 | -- | 10 | 1100 | 13.45 | 2 | 22 | Cuesta | CONDESAN |
| EC | Yanacocha.09 | PIC | Yanacocha | -0.13 | -78.58 | 3833 | 0.01 | Q | 10 x 10 | P | 1580 | 7.3 | 1 | 2012 | -- | 10 | 500 | 7.82 | 3 | 22 | Cuesta | CONDESAN |
| EC | Yanacocha.10 | PIC | Yanacocha | -0.13 | -78.58 | 3843 | 0.01 | Q | 10 x 10 | P | 1580 | 7.3 | 1 | 2012 | -- | 10 | 400 | 6.12 | 1 | 22 | Cuesta | CONDESAN |
| EC | Salva. Amazónica.01 | NAP | Selva Amazónica | -0.98 | -77.31 | 453 | 0.36 | Q | 60 x 60 | P | 3987 | 24.5 | 1 | 2012 | -- | 10 | 228 | 24.49 | 47 | -- | Cuesta | CONDESAN |
| EC | Salva. Amazónica.02 | NAP | Selva Amazónica | -0.98 | -77.31 | 395 | 0.36 | Q | 60 x 60 | P | 3987 | 24.5 | 1 | 2012 | -- | 10 | 200 | 20.56 | 34 | -- | Cuesta | CONDESAN |
| EC | Salva. Amazónica.03 | NAP | Selva Amazónica | -0.98 | -77.31 | 392 | 0.36 | Q | 60 x 60 | P | 3987 | 24.5 | 1 | 2012 | -- | 10 | 281 | 29.13 | 53 | -- | Cuesta | CONDESAN |
| EC | Napo1 | NAP | -- | -0.63 | -77.84 | 2092 | 0.04 | Q | 20 x 20 | NP | 3241 | 15.8 | 1 | 2011 | -- | 10 | 950 | 67.52 | 23 | -- | Baez | UPN |
| EC | Napo10 | NAP | -- | -0.65 | -77.80 | 1802 | 0.04 | Q | 20 x 20 | NP | 3619 | 17.3 | 1 | 2011 | -- | 10 | 1025 | 47.73 | 29 | -- | Baez | UPN |
| EC | Napo11 | NAP | -- | -0.64 | -77.78 | 1476 | 0.04 | Q | 20 x 20 | NP | 3024 | 18.6 | 1 | 2011 | -- | 10 | 275 | 17.96 | 9 | -- | Baez | UPN |
| EC | Napo12 | NAP | -- | -0.64 | -77.78 | 1602 | 0.04 | Q | 20 x 20 | NP | 3170 | 18.3 | 1 | 2011 | -- | 10 | 775 | 41.75 | 21 | -- | Baez | UPN |
| EC | Napo13 | NAP | -- | -0.65 | -77.79 | 1721 | 0.04 | Q | 20 x 20 | NP | 3462 | 17.9 | 1 | 2011 | -- | 10 | 675 | 40.09 | 20 | -- | Baez | UPN |
| EC | Napo14 | NAP | -- | -0.65 | -77.79 | 1769 | 0.04 | Q | 20 x 20 | NP | 3619 | 17.3 | 1 | 2011 | -- | 10 | 1200 | 52.63 | 24 | -- | Baez | UPN |
| EC | Napo15 | NAP | -- | -0.65 | -77.79 | 1778 | 0.04 | Q | 20 x 20 | NP | 3462 | 17.9 | 1 | 2011 | -- | 10 | 625 | 28.02 | 17 | -- | Baez | UPN |
| EC | Napo16 | NAP | -- | -0.62 | -77.82 | 2364 | 0.04 | Q | 20 x 20 | NP | 3390 | 15.5 | 1 | 2011 | -- | 10 | 850 | 49.11 | 15 | -- | Baez | UPN |
| EC | Napo17 | NAP | -- | -0.62 | -77.82 | 2383 | 0.04 | Q | 20 x 20 | NP | 3390 | 15.5 | 1 | 2011 | -- | 10 | 975 | 54.84 | 17 | -- | Baez | UPN |
| EC | Napo18 | NAP | -- | -0.62 | -77.82 | 2300 | 0.04 | Q | 20 x 20 | NP | 3390 | 15.5 | 1 | 2011 | -- | 10 | 900 | 53.07 | 13 | -- | Baez | UPN |
| EC | Napo19 | NAP | -- | -0.62 | -77.82 | 2273 | 0.04 | Q | 20 x 20 | NP | 3463 | 15.7 | 1 | 2011 | -- | 10 | 925 | 41.79 | 17 | -- | Baez | UPN |
| EC | Napo2 | NAP | -- | -0.63 | -77.84 | 2162 | 0.04 | Q | 20 x 20 | NP | 3241 | 15.8 | 1 | 2011 | -- | 10 | 875 | 131.20 | 17 | -- | Baez | UPN |
| EC | Napo20 | NAP | -- | -0.62 | -77.83 | 2336 | 0.04 | Q | 20 x 20 | NP | 3252 | 14.9 | 1 | 2011 | -- | 10 | 1200 | 43.80 | 21 | -- | Baez | UPN |
| EC | Napo21 | NAP | -- | -0.62 | -77.83 | 2416 | 0.04 | Q | 20 x 20 | NP | 3252 | 14.9 | 1 | 2011 | -- | 10 | 650 | 30.15 | 16 | -- | Baez | UPN |
| EC | Napo3 | NAP | -- | -0.63 | -77.84 | 2068 | 0.04 | Q | 20 x 20 | NP | 3241 | 15.8 | 1 | 2011 | -- | 10 | 650 | 20.06 | 12 | -- | Baez | UPN |
| EC | Napo4 | NAP | -- | -0.64 | -77.84 | 2079 | 0.04 | Q | 20 x 20 | NP | 3182 | 16.6 | 1 | 2011 | -- | 10 | 1075 | 40.42 | 14 | -- | Baez | UPN |
| EC | Napo5 | NAP | -- | -0.64 | -77.84 | 2006 | 0.04 | Q | 20 x 20 | NP | 3182 | 16.6 | 1 | 2011 | -- | 10 | 875 | 39.05 | 13 | -- | Baez | UPN |
| EC | Napo6 | NAP | -- | -0.65 | -77.80 | 1881 | 0.04 | Q | 20 x 20 | NP | 3594 | 16.6 | 1 | 2011 | -- | 10 | 675 | 33.64 | 17 | -- | Baez | UPN |
| EC | Napo7 | NAP | -- | -0.65 | -77.80 | 1918 | 0.04 | Q | 20 x 20 | NP | 3594 | 16.6 | 1 | 2011 | -- | 10 | 800 | 56.80 | 21 | -- | Baez | UPN |
| EC | Napo8 | NAP | -- | -0.65 | -77.80 | 1911 | 0.04 | Q | 20 x 20 | NP | 3594 | 16.6 | 1 | 2011 | -- | 10 | 825 | 35.00 | 15 | -- | Baez | UPN |
| EC | Napo9 | NAP | -- | -0.65 | -77.80 | 1873 | 0.04 | Q | 20 x 20 | NP | 3594 | 16.6 | 1 | 2011 | -- | 10 | 775 | 28.47 | 23 | -- | Baez | UPN |
| EC | Suc1 | SUC | -- | 0.62 | -77.50 | 2884 | 0.04 | Q | 20 x 20 | NP | 1405 | 11.1 | 1 | 2011 | -- | 10 | 1025 | 26.31 | 15 | -- | Baez | UPN |
| EC | Suc10 | SUC | -- | 0.63 | -77.52 | 2939 | 0.04 | Q | 20 x 20 | NP | 1329 | 10.6 | 1 | 2011 | -- | 10 | 900 | 43.70 | 17 | -- | Baez | UPN |
| EC | Suc2 | SUC | -- | 0.62 | -77.50 | 2884 | 0.04 | Q | 20 x 20 | NP | 1405 | 11.1 | 1 | 2011 | -- | 10 | 625 | 18.47 | 8 | -- | Baez | UPN |
| EC | Suc3 | SUC | -- | 0.61 | -77.51 | 2898 | 0.04 | Q | 20 x 20 | NP | 1362 | 11.7 | 1 | 2011 | -- | 10 | 775 | 25.29 | 15 | -- | Baez | UPN |
| EC | Suc4 | SUC | -- | 0.61 | -77.50 | 2887 | 0.04 | Q | 20 x 20 | NP | 1362 | 11.7 | 1 | 2011 | -- | 10 | 975 | 49.54 | 9 | -- | Baez | UPN |
| EC | Suc5 | SUC | -- | 0.62 | -77.50 | 2846 | 0.04 | Q | 20 x 20 | NP | 1405 | 11.1 | 1 | 2011 | -- | 10 | 925 | 25.83 | 11 | -- | Baez | UPN |
| EC | Suc6 | SUC | -- | 0.62 | -77.55 | 2979 | 0.04 | Q | 20 x 20 | NP | 1059 | 11.2 | 1 | 2011 | -- | 10 | 1525 | 55.66 | 19 | -- | Baez | UPN |
| EC | Suc7 | SUC | -- | 0.62 | -77.55 | 2992 | 0.04 | Q | 20 x 20 | NP | 1059 | 11.2 | 1 | 2011 | -- | 10 | 1350 | 52.99 | 18 | -- | Baez | UPN |
| EC | Suc8 | SUC | -- | 0.63 | -77.52 | 2962 | 0.04 | Q | 20 x 20 | NP | 1329 | 10.6 | 1 | 2011 | -- | 10 | 825 | 50.52 | 14 | -- | Baez | UPN |
| EC | Suc9 | SUC | -- | 0.63 | -77.52 | 2935 | 0.04 | Q | 20 x 20 | NP | 1329 | 10.6 | 1 | 2011 | -- | 10 | 1075 | 63.88 | 16 | -- | Baez | UPN |
| EC | MS1.1 | MSA | -- | -2.23 | -78.44 | 2884 | 0.04 | Q | 20 x 20 | NP | 1154 | 11.7 | 1 | 2011 | -- | 10 | 425 | 39.73 | 7 | -- | Baez | UPN |
| EC | MS1.10 | MSA | -- | -2.19 | -78.36 | 2530 | 0.04 | Q | 20 x 20 | NP | 1372 | 15.1 | 1 | 2011 | -- | 10 | 825 | 39.25 | 10 | -- | Baez | UPN |
| EC | MS1.11 | MSA | -- | -2.20 | -78.48 | 3266 | 0.04 | Q | 20 x 20 | NP | 1189 | 10.3 | 1 | 2011 | -- | 10 | 1400 | 57.46 | 6 | -- | Baez | UPN |
| EC | MS1.12 | MSA | -- | -2.20 | -78.48 | 3261 | 0.04 | Q | 20 x 20 | NP | 1189 | 10.3 | 1 | 2011 | -- | 10 | 550 | 57.61 | 9 | -- | Baez | UPN |
| EC | MS1.13 | MSA | -- | -2.20 | -78.48 | 3268 | 0.04 | Q | 20 x 20 | NP | 1189 | 10.3 | 1 | 2011 | -- | 10 | 500 | 25.79 | 6 | -- | Baez | UPN |
| EC | MS1.14 | MSA | -- | -2.20 | -78.48 | 3257 | 0.04 | Q | 20 x 20 | NP | 1189 | 10.3 | 1 | 2011 | -- | 10 | 700 | 51.92 | 11 | -- | Baez | UPN |
| EC | MS1.15 | MSA | -- | -2.20 | -78.48 | 3270 | 0.04 | Q | 20 x 20 | NP | 1243 | 8.1 | 1 | 2011 | -- | 10 | 550 | 43.18 | 7 | -- | Baez | UPN |
| EC | MS1.2 | MSA | -- | -2.23 | -78.44 | 2873 | 0.04 | Q | 20 x 20 | NP | 1154 | 11.7 | 1 | 2011 | -- | 10 | 1050 | 62.67 | 12 | -- | Baez | UPN |
| EC | MS1.3 | MSA | -- | -2.22 | -78.44 | 2866 | 0.04 | Q | 20 x 20 | NP | 1240 | 11.4 | 1 | 2011 | -- | 10 | 700 | 51.18 | 9 | -- | Baez | UPN |
| EC | MS1.4 | MSA | -- | -2.20 | -78.46 | 2859 | 0.04 | Q | 20 x 20 | NP | 1338 | 10 | 1 | 2011 | -- | 10 | 750 | 63.36 | 17 | -- | Baez | UPN |
| EC | MS1.5 | MSA | -- | -2.20 | -78.45 | 2878 | 0.04 | Q | 20 x 20 | NP | 1338 | 10 | 1 | 2011 | -- | 10 | 650 | 31.32 | 14 | -- | Baez | UPN |
| EC | MS1.6 | MSA | -- | -2.20 | -78.36 | 2296 | 0.04 | Q | 20 x 20 | NP | 1286 | 14.7 | 1 | 2011 | -- | 10 | 775 | 34.82 | 17 | -- | Baez | UPN |
| EC | MS1.7 | MSA | -- | -2.20 | -78.37 | 2399 | 0.04 | Q | 20 x 20 | NP | 1263 | 15.2 | 1 | 2011 | -- | 10 | 750 | 43.55 | 18 | -- | Baez | UPN |
| EC | MS1.8 | MSA | -- | -2.20 | -78.37 | 2361 | 0.04 | Q | 20 x 20 | NP | 1286 | 14.7 | 1 | 2011 | -- | 10 | 950 | 27.08 | 18 | -- | Baez | UPN |
| EC | MS1.9 | MSA | -- | -2.20 | -78.37 | 2385 | 0.04 | Q | 20 x 20 | NP | 1263 | 15.2 | 1 | 2011 | -- | 10 | 825 | 53.43 | 16 | -- | Baez | UPN |
| EC | ZC1 | ZCH | -- | -4.61 | -78.92 | 1853 | 0.04 | Q | 20 x 20 | NP | 1350 | 13.6 | 1 | 2011 | -- | 10 | 875 | 47.40 | 19 | -- | Baez | UPN |
| EC | ZC10 | ZCH | -- | -4.61 | -78.91 | 2107 | 0.04 | Q | 20 x 20 | NP | 1434 | 16.2 | 1 | 2011 | -- | 10 | 1000 | 25.03 | 27 | -- | Baez | UPN |
| EC | ZC11 | ZCH | -- | -4.62 | -78.94 | 1692 | 0.04 | Q | 20 x 20 | NP | 888 | 18.6 | 1 | 2011 | -- | 10 | 1025 | 49.04 | 21 | -- | Baez | UPN |
| EC | ZC12 | ZCH | -- | -4.62 | -78.96 | 1659 | 0.04 | Q | 20 x 20 | NP | 1062 | 17.7 | 1 | 2011 | -- | 10 | 875 | 44.24 | 18 | -- | Baez | UPN |
| EC | ZC13 | ZCH | -- | -4.62 | -78.96 | 1649 | 0.04 | Q | 20 x 20 | NP | 1062 | 17.7 | 1 | 2011 | -- | 10 | 775 | 32.74 | 17 | -- | Baez | UPN |
| EC | ZC14 | ZCH | -- | -4.62 | -78.95 | 1697 | 0.04 | Q | 20 x 20 | NP | 888 | 18.6 | 1 | 2011 | -- | 10 | 825 | 45.55 | 16 | -- | Baez | UPN |
| EC | ZC15 | ZCH | -- | -4.62 | -78.96 | 1644 | 0.04 | Q | 20 x 20 | NP | 1062 | 17.7 | 1 | 2011 | -- | 10 | 1275 | 41.62 | 22 | -- | Baez | UPN |
| EC | ZC16 | ZCH | -- | -4.61 | -78.90 | 2060 | 0.04 | Q | 20 x 20 | NP | 1434 | 16.2 | 1 | 2011 | -- | 10 | 675 | 15.42 | 12 | -- | Baez | UPN |
| EC | ZC17 | ZCH | -- | -4.61 | -78.90 | 2067 | 0.04 | Q | 20 x 20 | NP | 1434 | 16.2 | 1 | 2011 | -- | 10 | 625 | 18.73 | 16 | -- | Baez | UPN |
| EC | ZC18 | ZCH | -- | -4.61 | -78.90 | 2016 | 0.04 | Q | 20 x 20 | NP | 1434 | 16.2 | 1 | 2011 | -- | 10 | 825 | 26.65 | 12 | -- | Baez | UPN |
| EC | ZC19 | ZCH | -- | -4.61 | -78.90 | 2047 | 0.04 | Q | 20 x 20 | NP | 1434 | 16.2 | 1 | 2011 | -- | 10 | 375 | 12.37 | 11 | -- | Baez | UPN |
| EC | ZC2 | ZCH | -- | -4.62 | -78.92 | 1835 | 0.04 | Q | 20 x 20 | NP | 1176 | 14.9 | 1 | 2011 | -- | 10 | 975 | 39.20 | 17 | -- | Baez | UPN |
| EC | ZC20 | ZCH | -- | -4.61 | -78.91 | 2083 | 0.04 | Q | 20 x 20 | NP | 1434 | 16.2 | 1 | 2011 | -- | 10 | 400 | 10.50 | 10 | -- | Baez | UPN |
| EC | ZC3 | ZCH | -- | -4.62 | -78.92 | 1829 | 0.04 | Q | 20 x 20 | NP | 1880 | 21.4 | 1 | 2011 | -- | 10 | 1000 | 48.04 | 18 | -- | Baez | UPN |
| EC | ZC4 | ZCH | -- | -4.61 | -78.92 | 1912 | 0.04 | Q | 20 x 20 | NP | 2046 | 21.2 | 1 | 2011 | -- | 10 | 1125 | 51.64 | 20 | -- | Baez | UPN |
| EC | ZC5 | ZCH | -- | -4.61 | -78.92 | 1918 | 0.04 | Q | 20 x 20 | NP | 1210 | 17.2 | 1 | 2011 | -- | 10 | 1250 | 43.64 | 21 | -- | Baez | UPN |
| EC | ZC6 | ZCH | -- | -4.61 | -78.91 | 2094 | 0.04 | Q | 20 x 20 | NP | 1210 | 17.2 | 1 | 2011 | -- | 10 | 550 | 9.72 | 13 | -- | Baez | UPN |
| EC | ZC7 | ZCH | -- | -4.61 | -78.91 | 2035 | 0.04 | Q | 20 x 20 | NP | 1434 | 16.2 | 1 | 2011 | -- | 10 | 1025 | 31.15 | 16 | -- | Baez | UPN |
| EC | ZC8 | ZCH | -- | -4.60 | -78.91 | 2157 | 0.04 | Q | 20 x 20 | NP | 1434 | 16.2 | 1 | 2011 | -- | 10 | 1250 | 72.85 | 25 | -- | Baez | UPN |
| EC | ZC9 | ZCH | -- | -4.61 | -78.91 | 2108 | 0.04 | Q | 20 x 20 | NP | 1621 | 15.3 | 1 | 2011 | -- | 10 | 850 | 26.22 | 13 | -- | Baez | UPN |
| EC | ZCh1 | ZCH | -- | -4.49 | -79.12 | 2419 | 1 | Q | 100 x 100 | NP | 1434 | 16.2 | 1 | 2009 | -- | 10 | 400 | 24.92 | 76 | -- | Baez | UPN |
| EC | ZCh2 | ZCH | -- | -4.41 | -79.06 | 2109 | 1 | Q | 100 x 100 | NP | 1350 | 13.6 | 1 | 2009 | -- | 10 | 620 | 44.65 | 118 | -- | Baez | UPN |
| EC | ZCh3 | ZCH | -- | -3.73 | -78.62 | 928 | 1 | Q | 100 x 100 | NP | 1176 | 14.9 | 1 | 2009 | -- | 10 | 660 | 27.89 | 79 | -- | Baez | UPN |
| EC | ZCh4 | ZCH | -- | -3.73 | -78.62 | 1055 | 1 | Q | 100 x 100 | NP | 1880 | 21.4 | 1 | 2009 | -- | 10 | 619 | 29.28 | 81 | -- | Baez | UPN |
| EC | Gañadel.10 | AZU | Azuay | -3.17 | -79.02 | 3372 | 0.05 | R | 25 x 20 | P | 2046 | 21.2 | 1 | 2015 | -- | 5 | 1180 | 44.43 | 11 | 52 | Jadán | UCuenca |
| EC | Gañadel.6 | AZU | Azuay | -3.16 | -79.04 | 3400 | 0.05 | R | 25 x 20 | P | 925 | 9.2 | 1 | 2015 | -- | 5 | 1480 | 36.48 | 11 | 48 | Jadán | UCuenca |
| EC | Gañadel.7 | AZU | Azuay | -3.15 | -79.04 | 3411 | 0.05 | R | 25 x 20 | P | 994 | 9.2 | 1 | 2015 | -- | 5 | 1220 | 42.44 | 14 | 52 | Jadán | UCuenca |
| EC | Gañadel.8 | AZU | Azuay | -3.16 | -79.04 | 3168 | 0.05 | R | 25 x 20 | P | 980 | 8.9 | 1 | 2015 | -- | 5 | 1460 | 41.41 | 12 | 50 | Jadán | UCuenca |
| EC | Gañadel.9 | AZU | Azuay | -3.17 | -79.03 | 3411 | 0.05 | R | 25 x 20 | P | 1001 | 8.7 | 1 | 2015 | -- | 5 | 1300 | 50.52 | 10 | 60 | Jadán | UCuenca |
| EC | Irquis.11 | AZU | Azuay | -3.06 | -79.17 | 3372 | 0.05 | R | 25 x 20 | P | 790 | 8.8 | 1 | 2015 | -- | 5 | 960 | 25.35 | 12 | 28 | Jadán | UCuenca |
| EC | Irquis.12 | AZU | Azuay | -3.06 | -79.16 | 3385 | 0.05 | R | 25 x 20 | P | 822 | 8.8 | 1 | 2015 | -- | 5 | 1100 | 28.49 | 15 | 37 | Jadán | UCuenca |
| EC | Irquis.13 | AZU | Azuay | -3.07 | -79.16 | 3290 | 0.05 | R | 25 x 20 | P | 841 | 9.9 | 1 | 2015 | -- | 5 | 1380 | 32.69 | 23 | 28 | Jadán | UCuenca |
| EC | Irquis.14 | AZU | Azuay | -3.07 | -79.15 | 3218 | 0.05 | R | 25 x 20 | P | 841 | 9.9 | 1 | 2015 | -- | 5 | 440 | 7.30 | 9 | 33 | Jadán | UCuenca |
| EC | Irquis.15 | AZU | Azuay | -3.08 | -79.11 | 3011 | 0.05 | R | 25 x 20 | P | 888 | 10.8 | 1 | 2015 | -- | 5 | 560 | 7.23 | 11 | 35 | Jadán | UCuenca |
| EC | Irquis.16 | AZU | Azuay | -3.08 | -79.12 | 3100 | 0.05 | R | 25 x 20 | P | 897 | 10.5 | 1 | 2015 | -- | 5 | 800 | 11.29 | 9 | 27 | Jadán | UCuenca |
| EC | Irquis.17 | AZU | Azuay | -3.08 | -79.11 | 3050 | 0.05 | R | 25 x 20 | P | 888 | 10.8 | 1 | 2015 | -- | 5 | 960 | 12.75 | 12 | 38 | Jadán | UCuenca |
| EC | Pillachiquir.1 | AZU | Azuay | -3.14 | -78.98 | 3210 | 0.05 | R | 25 x 20 | P | 1013 | 10.8 | 1 | 2015 | -- | 5 | 180 | 2.40 | 7 | 26 | Jadán | UCuenca |
| EC | Pillachiquir.2 | AZU | Azuay | -3.14 | -78.98 | 3100 | 0.05 | R | 25 x 20 | P | 1043 | 10.4 | 1 | 2015 | -- | 5 | 720 | 17.01 | 14 | 46 | Jadán | UCuenca |
| EC | Pillachiquir.3 | AZU | Azuay | -3.13 | -78.99 | 3151 | 0.05 | R | 25 x 20 | P | 1006 | 10.5 | 1 | 2015 | -- | 5 | 860 | 12.56 | 10 | 36 | Jadán | UCuenca |
| EC | Pillachiquir.4 | AZU | Azuay | -3.12 | -78.99 | 3120 | 0.05 | R | 25 x 20 | P | 968 | 10.6 | 1 | 2015 | -- | 5 | 660 | 8.88 | 11 | 41 | Jadán | UCuenca |
| EC | Pillachiquir.5 | AZU | Azuay | -3.12 | -78.99 | 3083 | 0.05 | R | 25 x 20 | P | 968 | 10.6 | 1 | 2015 | -- | 5 | 700 | 8.33 | 8 | 35 | Jadán | UCuenca |
| EC | SantaAna.18 | AZU | Azuay | -2.99 | -78.86 | 3232 | 0.05 | R | 25 x 20 | P | 1114 | 10.3 | 1 | 2015 | -- | 5 | 160 | 2.03 | 5 | 52 | Jadán | UCuenca |
| EC | SantaAna.19 | AZU | Azuay | -2.99 | -78.87 | 3196 | 0.05 | R | 25 x 20 | P | 1046 | 10.8 | 1 | 2015 | -- | 5 | 1280 | 20.08 | 5 | 40 | Jadán | UCuenca |
| EC | SantaAna.20 | AZU | Azuay | -3.00 | -78.87 | 3210 | 0.05 | R | 25 x 20 | P | 1075 | 10.2 | 1 | 2015 | -- | 5 | 760 | 10.13 | 6 | 54 | Jadán | UCuenca |
| EC | Colorado | LOJ | Loja | -3.99 | -79.97 | 1500 | 1 | Q | 100 x 100 | P | 1265 | 17.8 | 2 | 2005 2012 | 7 | 10 | 514 | 23.12 | 46 | -- | Aguirre | UNL |
| EC | El Limo | LOJ | Loja | -3.98 | -80.14 | 1380 | 1 | Q | 100 x 100 | P | 1031 | 20 | 1 | 2015 | 6 | 10 | 478 | 18.91 | 36 | -- | Aguirre | UNL |
| EC | Bombuscaro.A1 | ZCH | PN Podocarpus | -4.12 | -78.97 | 1084 | 0.1 | R | 50 x 20 | NP | 1046 | 20.9 | 1 | 2015 | -- | 2.5 | 870 | 34.50 | 36 | -- | Cayuela | URJC |
| EC | Bombuscaro.A2 | ZCH | PN Podocarpus | -4.11 | -78.69 | 1053 | 0.1 | R | 50 x 20 | NP | 1116 | 20.1 | 1 | 2015 | -- | 2.5 | 670 | 25.87 | 24 | -- | Cayuela | URJC |
| EC | Bombuscaro.A3 | ZCH | PN Podocarpus | -4.12 | -78.97 | 1057 | 0.1 | R | 50 x 20 | NP | 2022 | 19.9 | 1 | 2015 | -- | 2.5 | 910 | 34.76 | 42 | -- | Cayuela | URJC |
| EC | Bombuscaro.A4 | ZCH | PN Podocarpus | -4.12 | -78.96 | 1104 | 0.1 | R | 50 x 20 | NP | 1046 | 20.9 | 1 | 2015 | -- | 2.5 | 960 | 31.84 | 45 | -- | Cayuela | URJC |
| EC | Bombuscaro.A5 | ZCH | PN Podocarpus | -4.11 | -78.96 | 1033 | 0.1 | R | 50 x 20 | NP | 1122 | 19.4 | 1 | 2015 | -- | 2.5 | 920 | 28.19 | 55 | -- | Cayuela | URJC |
| EC | Bombuscaro.A6 | ZCH | PN Podocarpus | -4.12 | -78.98 | 1084 | 0.1 | R | 50 x 20 | NP | 1046 | 21.1 | 1 | 2015 | -- | 2.5 | 430 | 23.17 | 28 | -- | Cayuela | URJC |
| EC | Bombuscaro.A7 | ZCH | PN Podocarpus | -4.12 | -78.98 | 1129 | 0.1 | R | 50 x 20 | NP | 1111 | 19.9 | 1 | 2015 | -- | 2.5 | 550 | 28.39 | 24 | -- | Cayuela | URJC |
| EC | Bombuscaro.A8 | ZCH | PN Podocarpus | -4.13 | -78.98 | 1216 | 0.1 | R | 50 x 20 | NP | 1111 | 19.9 | 1 | 2015 | -- | 2.5 | 720 | 67.50 | 28 | -- | Cayuela | URJC |
| EC | Bombuscaro.A9 | ZCH | PN Podocarpus | -4.13 | -78.98 | 1249 | 0.1 | R | 50 x 20 | NP | 957 | 20.7 | 1 | 2015 | -- | 2.5 | 660 | 59.89 | 35 | -- | Cayuela | URJC |
| EC | Bombuscaro.A10 | ZCH | PN Podocarpus | -4.11 | -78.97 | 1130 | 0.1 | R | 50 x 20 | NP | 1116 | 20.1 | 1 | 2015 | -- | 2.5 | 930 | 24.69 | 41 | -- | Cayuela | URJC |
| EC | Bombuscaro.B1 | ZCH | PN Podocarpus | -4.13 | -79.01 | 1806 | 0.1 | R | 50 x 20 | NP | 1390 | 16.5 | 1 | 2015 | -- | 2.5 | 910 | 29.60 | 42 | -- | Cayuela | URJC |
| EC | Bombuscaro.B2 | ZCH | PN Podocarpus | -4.13 | -79.01 | 1837 | 0.1 | R | 50 x 20 | NP | 1390 | 16.5 | 1 | 2015 | -- | 2.5 | 610 | 26.43 | 27 | -- | Cayuela | URJC |
| EC | Bombuscaro.B3 | ZCH | PN Podocarpus | -4.13 | -79.01 | 1952 | 0.1 | R | 50 x 20 | NP | 1390 | 16.5 | 1 | 2015 | -- | 2.5 | 820 | 32.29 | 46 | -- | Cayuela | URJC |
| EC | Bombuscaro.B4 | ZCH | PN Podocarpus | -4.13 | -79.02 | 1851 | 0.1 | R | 50 x 20 | NP | 1390 | 16.5 | 1 | 2015 | -- | 2.5 | 800 | 46.12 | 41 | -- | Cayuela | URJC |
| EC | Bombuscaro.B5 | ZCH | PN Podocarpus | -4.13 | -79.02 | 1860 | 0.1 | R | 50 x 20 | NP | 1391 | 16.4 | 1 | 2015 | -- | 2.5 | 980 | 66.06 | 43 | -- | Cayuela | URJC |
| EC | Bombuscaro.B6 | ZCH | PN Podocarpus | -4.13 | -79.02 | 1901 | 0.1 | R | 50 x 20 | NP | 1391 | 16.4 | 1 | 2015 | -- | 2.5 | 1010 | 27.15 | 34 | -- | Cayuela | URJC |
| EC | Bombuscaro.B7 | ZCH | PN Podocarpus | -4.13 | -79.01 | 2217 | 0.1 | R | 50 x 20 | NP | 1390 | 16.5 | 1 | 2015 | -- | 2.5 | 730 | 37.75 | 33 | -- | Cayuela | URJC |
| EC | Bombuscaro.B8 | ZCH | PN Podocarpus | -4.13 | -79.01 | 2060 | 0.1 | R | 50 x 20 | NP | 1390 | 16.5 | 1 | 2015 | -- | 2.5 | 1010 | 31.79 | 38 | -- | Cayuela | URJC |
| EC | Bombuscaro.B9 | ZCH | PN Podocarpus | -4.13 | -79.02 | 2102 | 0.1 | R | 50 x 20 | NP | 1391 | 16.4 | 1 | 2015 | -- | 2.5 | 740 | 27.43 | 46 | -- | Cayuela | URJC |
| EC | Bombuscaro.B10 | ZCH | PN Podocarpus | -4.13 | -79.03 | 2190 | 0.1 | R | 50 x 20 | NP | 1339 | 16.3 | 1 | 2015 | -- | 2.5 | 560 | 16.20 | 27 | -- | Cayuela | URJC |
| EC | Bombuscaro.C1 | ZCH | PN Podocarpus | -4.11 | -79.02 | 2817 | 0.1 | R | 50 x 20 | NP | 1544 | 13.1 | 1 | 2015 | -- | 2.5 | 350 | 5.56 | 17 | -- | Cayuela | URJC |
| EC | Bombuscaro.C2 | ZCH | PN Podocarpus | -4.11 | -79.02 | 2796 | 0.1 | R | 50 x 20 | NP | 1544 | 13.1 | 1 | 2015 | -- | 2.5 | 750 | 16.93 | 24 | -- | Cayuela | URJC |
| EC | Bombuscaro.C3 | ZCH | PN Podocarpus | -4.10 | -79.02 | 2851 | 0.1 | R | 50 x 20 | NP | 1628 | 12.3 | 1 | 2015 | -- | 2.5 | 560 | 6.78 | 16 | -- | Cayuela | URJC |
| EC | Bombuscaro.C4 | ZCH | PN Podocarpus | -4.10 | -79.02 | 2906 | 0.1 | R | 50 x 20 | NP | 1628 | 12.3 | 1 | 2015 | -- | 2.5 | 530 | 10.59 | 19 | -- | Cayuela | URJC |
| EC | Bombuscaro.C5 | ZCH | PN Podocarpus | -4.11 | -79.03 | 2900 | 0.1 | R | 50 x 20 | NP | 1544 | 13.1 | 1 | 2015 | -- | 2.5 | 560 | 9.80 | 23 | -- | Cayuela | URJC |
| EC | Bombuscaro.C6 | ZCH | PN Podocarpus | -4.11 | -79.02 | 2729 | 0.1 | R | 50 x 20 | NP | 1544 | 13.1 | 1 | 2015 | -- | 2.5 | 490 | 11.97 | 14 | -- | Cayuela | URJC |
| EC | Bombuscaro.C7 | ZCH | PN Podocarpus | -4.11 | -79.02 | 2703 | 0.1 | R | 50 x 20 | NP | 1544 | 13.1 | 1 | 2015 | -- | 2.5 | 660 | 10.29 | 18 | -- | Cayuela | URJC |
| EC | Bombuscaro.C8 | ZCH | PN Podocarpus | -4.10 | -79.02 | 2738 | 0.1 | R | 50 x 20 | NP | 1655 | 13 | 1 | 2015 | -- | 2.5 | 870 | 17.79 | 19 | -- | Cayuela | URJC |
| EC | Bombuscaro.C9 | ZCH | PN Podocarpus | -4.10 | -79.02 | 2765 | 0.1 | R | 50 x 20 | NP | 1628 | 12.3 | 1 | 2015 | -- | 2.5 | 600 | 8.80 | 17 | -- | Cayuela | URJC |
| EC | Bombuscaro.C10 | ZCH | PN Podocarpus | -4.12 | -79.02 | 2674 | 0.1 | R | 50 x 20 | NP | 1467 | 14.1 | 1 | 2015 | -- | 2.5 | 860 | 14.04 | 16 | -- | Cayuela | URJC |
| EC | CAJ_LS1 | LOJ | Cajanuma | -4.112 | -79.179 | 2818 | 0.04 | Q | 20 x 20 | P | 888 | 12.1 | 3 | 2008 2009 2016 | 1, 7 | 5 | 500 | 49.76 | 19 | -- | Homeier | Univ. Goettingen |
| EC | CAJ_LS2 | LOJ | Cajanuma | -4.112 | -79.179 | 2805 | 0.04 | Q | 20 x 20 | P | 888 | 12.1 | 3 | 2008 2009 2016 | 1, 7 | 5 | 400 | 38.65 | 11 | -- | Homeier | Univ. Goettingen |
| EC | CAJ_LS3 | LOJ | Cajanuma | -4.112 | -79.18 | 2798 | 0.04 | Q | 20 x 20 | P | 888 | 12.1 | 3 | 2008 2009 2016 | 1, 7 | 5 | 500 | 39.08 | 12 | -- | Homeier | Univ. Goettingen |
| EC | CAJ_LS4 | LOJ | Cajanuma | -4.112 | -79.183 | 2865 | 0.04 | Q | 20 x 20 | P | 888 | 12.1 | 3 | 2008 2009 2016 | 1, 7 | 5 | 1100 | 64.52 | 19 | -- | Homeier | Univ. Goettingen |
| EC | CAJ_LS5 | LOJ | Cajanuma | -4.112 | -79.183 | 2878 | 0.04 | Q | 20 x 20 | P | 888 | 12.1 | 3 | 2008 2009 2016 | 1, 7 | 5 | 1275 | 49.80 | 21 | -- | Homeier | Univ. Goettingen |
| EC | CAJ_LS6 | LOJ | Cajanuma | -4.112 | -79.183 | 2880 | 0.04 | Q | 20 x 20 | P | 888 | 12.1 | 3 | 2008 2009 2016 | 1, 7 | 5 | 1075 | 40.78 | 27 | -- | Homeier | Univ. Goettingen |
| EC | CAJ_MS1 | LOJ | Cajanuma | -4.113 | -79.18 | 2859 | 0.04 | Q | 20 x 20 | P | 888 | 12.1 | 3 | 2008 2009 2016 | 1, 7 | 5 | 1375 | 42.44 | 20 | -- | Homeier | Univ. Goettingen |
| EC | CAJ_MS2 | LOJ | Cajanuma | -4.112 | -79.181 | 2872 | 0.04 | Q | 20 x 20 | P | 888 | 12.1 | 3 | 2008 2009 2016 | 1, 7 | 5 | 1175 | 38.82 | 17 | -- | Homeier | Univ. Goettingen |
| EC | CAJ_MS3 | LOJ | Cajanuma | -4.112 | -79.18 | 2844 | 0.04 | Q | 20 x 20 | P | 888 | 12.1 | 3 | 2008 2009 2016 | 1, 7 | 5 | 1450 | 44.18 | 20 | -- | Homeier | Univ. Goettingen |
| EC | CAJ_MS4 | LOJ | Cajanuma | -4.117 | -79.177 | 2900 | 0.04 | Q | 20 x 20 | P | 845 | 13.8 | 3 | 2008 2009 2016 | 1, 7 | 5 | 525 | 21.10 | 14 | -- | Homeier | Univ. Goettingen |
| EC | CAJ_MS5 | LOJ | Cajanuma | -4.118 | -79.177 | 2885 | 0.04 | Q | 20 x 20 | P | 845 | 13.8 | 3 | 2008 2009 2016 | 1, 7 | 5 | 725 | 50.14 | 21 | -- | Homeier | Univ. Goettingen |
| EC | CAJ_MS6 | LOJ | Cajanuma | -4.118 | -79.178 | 2875 | 0.04 | Q | 20 x 20 | P | 845 | 13.8 | 3 | 2008 2009 2016 | 1, 7 | 5 | 950 | 29.18 | 13 | -- | Homeier | Univ. Goettingen |
| EC | CAJ_US1 | LOJ | Cajanuma | -4.112 | -79.181 | 2891 | 0.04 | Q | 20 x 20 | P | 888 | 12.1 | 3 | 2008 2009 2016 | 1, 7 | 5 | 1025 | 47.30 | 25 | -- | Homeier | Univ. Goettingen |
| EC | CAJ_US2 | LOJ | Cajanuma | -4.112 | -79.181 | 2885 | 0.04 | Q | 20 x 20 | P | 888 | 12.1 | 3 | 2008 2009 2016 | 1, 7 | 5 | 1025 | 32.65 | 18 | -- | Homeier | Univ. Goettingen |
| EC | CAJ_US3 | LOJ | Cajanuma | -4.112 | -79.181 | 2869 | 0.04 | Q | 20 x 20 | P | 888 | 12.1 | 3 | 2008 2009 2016 | 1, 7 | 5 | 1475 | 39.80 | 13 | -- | Homeier | Univ. Goettingen |
| EC | CAJ_US4 | LOJ | Cajanuma | -4.112 | -79.183 | 2886 | 0.04 | Q | 20 x 20 | P | 888 | 12.1 | 3 | 2008 2009 2016 | 1, 7 | 5 | 1075 | 30.27 | 17 | -- | Homeier | Univ. Goettingen |
| EC | CAJ_US5 | LOJ | Cajanuma | -4.112 | -79.183 | 2890 | 0.04 | Q | 20 x 20 | P | 888 | 12.1 | 3 | 2008 2009 2016 | 1, 7 | 5 | 1325 | 44.44 | 18 | -- | Homeier | Univ. Goettingen |
| EC | CAJ_US6 | LOJ | Cajanuma | -4.112 | -79.183 | 2893 | 0.04 | Q | 20 x 20 | P | 888 | 12.1 | 3 | 2008 2009 2016 | 1, 7 | 5 | 1025 | 31.35 | 15 | -- | Homeier | Univ. Goettingen |
| EC | ALC_91 | NAP | Alcantarillas | -0.371 | -78.121 | 3100 | 0.04 | Q | 20 x 20 | P | 925 | 10.2 | 2 | 2011 2012 | 1 | 5 | 1525 | 36.85 | 61 | -- | Homeier | Univ. Goettingen |
| EC | ALC_92 | NAP | Alcantarillas | -0.371 | -78.121 | 3100 | 0.04 | Q | 20 x 20 | P | 925 | 10.2 | 2 | 2012 2012 | 1 | 5 | 1150 | 33.86 | 12 | -- | Homeier | Univ. Goettingen |
| EC | COC_76 | NAP | Cocodrilos | -0.64 | -77.785 | 1550 | 0.04 | Q | 20 x 20 | P | 3170 | 18.3 | 1 | 2007 | -- | 5 | 900 | 57.5 | 23 | -- | Homeier | Univ. Goettingen |
| EC | COC_77 | NAP | Cocodrilos | -0.639 | -77.782 | 1490 | 0.04 | Q | 20 x 20 | P | 3024 | 18.6 | 2 | 2007 2008 | 1 | 5 | 850 | 56.25 | 19 | -- | Homeier | Univ. Goettingen |
| EC | COC_78 | NAP | Cocodrilos | -0.638 | -77.782 | 1490 | 0.04 | Q | 20 x 20 | P | 3024 | 18.6 | 1 | 2007 | -- | 5 | 850 | 49.75 | 22 | -- | Homeier | Univ. Goettingen |
| EC | COC_79 | NAP | Cocodrilos | -0.638 | -77.782 | 1490 | 0.04 | Q | 20 x 20 | P | 3024 | 18.6 | 2 | 2007 2008 | 1 | 5 | 875 | 54.75 | 21 | -- | Homeier | Univ. Goettingen |
| EC | COC_80 | NAP | Cocodrilos | -0.638 | -77.786 | 1570 | 0.04 | Q | 20 x 20 | P | 3170 | 18.3 | 2 | 2007 2008 | 1 | 5 | 425 | 59.5 | 13 | -- | Homeier | Univ. Goettingen |
| EC | GAL_20 | NAP | Galeras | -0.829 | -77.567 | 1090 | 0.04 | Q | 20 x 20 | P | 3770 | 20.5 | 3 | 2005 2008 2011 | 3, 3 | 5 | 725 | 71.5 | 19 | -- | Homeier | Univ. Goettingen |
| EC | GAL_21 | NAP | Galeras | -0.83 | -77.568 | 1080 | 0.04 | Q | 20 x 20 | P | 3770 | 20.5 | 1 | 2005 | -- | 5 | 475 | 53.5 | 11 | -- | Homeier | Univ. Goettingen |
| EC | GAL_22 | NAP | Galeras | -0.814 | -77.578 | 1060 | 0.04 | Q | 20 x 20 | P | 3745 | 21.2 | 3 | 2005 2008 2011 | 3, 3 | 5 | 825 | 53.5 | 24 | -- | Homeier | Univ. Goettingen |
| EC | GAL_23 | NAP | Galeras | -0.815 | -77.577 | 1090 | 0.04 | Q | 20 x 20 | P | 3745 | 21.2 | 1 | 2005 | -- | 5 | 675 | 67.5 | 23 | -- | Homeier | Univ. Goettingen |
| EC | GAL_24 | NAP | Galeras | -0.838 | -77.56 | 1110 | 0.04 | Q | 20 x 20 | P | 3727 | 20.6 | 3 | 2005 2008 2011 | 3, 3 | 5 | 825 | 37.25 | 24 | -- | Homeier | Univ. Goettingen |
| EC | GAL_25 | NAP | Galeras | -0.828 | -77.543 | 1450 | 0.04 | Q | 20 x 20 | P | 4414 | 19.4 | 3 | 2005 2008 2011 | 3, 3 | 5 | 950 | 58.5 | 27 | -- | Homeier | Univ. Goettingen |
| EC | GAL_26 | NAP | Galeras | -0.828 | -77.539 | 1560 | 0.04 | Q | 20 x 20 | P | 5059 | 18.5 | 3 | 2005 2008 2011 | 3, 3 | 5 | 800 | 42.25 | 24 | -- | Homeier | Univ. Goettingen |
| EC | GAL_28 | NAP | Galeras | -0.829 | -77.538 | 1597 | 0.04 | Q | 20 x 20 | P | 5059 | 18.5 | 1 | 2006 | -- | 5 | 1125 | 35.5 | 26 | -- | Homeier | Univ. Goettingen |
| EC | GAL_29 | NAP | Galeras | -0.813 | -77.578 | 1050 | 0.04 | Q | 20 x 20 | P | 3745 | 21.2 | 1 | 2006 | -- | 5 | 900 | 47.25 | 25 | -- | Homeier | Univ. Goettingen |
| EC | GAL_47 | NAP | Galeras | -0.829 | -77.568 | 1000 | 0.04 | Q | 20 x 20 | P | 3770 | 20.5 | 1 | 2006 | -- | 5 | 600 | 33.5 | 18 | -- | Homeier | Univ. Goettingen |
| EC | GAL_48 | NAP | Galeras | -0.838 | -77.56 | 1130 | 0.04 | Q | 20 x 20 | P | 3727 | 20.6 | 1 | 2006 | -- | 5 | 725 | 41 | 23 | -- | Homeier | Univ. Goettingen |
| EC | GAL_49 | NAP | Galeras | -0.815 | -77.577 | 1080 | 0.04 | Q | 20 x 20 | P | 3745 | 21.2 | 1 | 2006 | -- | 5 | 850 | 46.25 | 23 | -- | Homeier | Univ. Goettingen |
| EC | GAL_50 | NAP | Galeras | -0.829 | -77.537 | 1557 | 0.04 | Q | 20 x 20 | P | 5059 | 18.5 | 1 | 2006 | -- | 5 | 1200 | 35 | 30 | -- | Homeier | Univ. Goettingen |
| EC | GAL_60 | NAP | Galeras | -0.829 | -77.536 | 1570 | 0.04 | Q | 20 x 20 | P | 5059 | 18.5 | 3 | 2006 2008 2011 | 2, 3 | 5 | 925 | 40.25 | 26 | -- | Homeier | Univ. Goettingen |
| EC | GAL_61 | NAP | Galeras | -0.83 | -77.536 | 1560 | 0.04 | Q | 20 x 20 | P | 5059 | 18.5 | 1 | 2006 | -- | 5 | 825 | 43.5 | 22 | -- | Homeier | Univ. Goettingen |
| EC | GAL_62 | NAP | Galeras | -0.829 | -77.538 | 1590 | 0.04 | Q | 20 x 20 | P | 5059 | 18.5 | 1 | 2006 | -- | 5 | 1200 | 37 | 23 | -- | Homeier | Univ. Goettingen |
| EC | GAN_90 | NAP | Guango | -0.373 | -78.075 | 2890 | 0.04 | Q | 20 x 20 | P | 791 | 11 | 2 | 2011 2012 | 1 | 5 | 1025 | 31.86 | 15 | -- | Homeier | Univ. Goettingen |
| EC | GUA_06 | NAP | Guacamayos | -0.638 | -77.84 | 2000 | 0.04 | Q | 20 x 20 | P | 3182 | 16.6 | 3 | 2005 2008 2011 | 3, 3 | 5 | 700 | 25 | 18 | -- | Homeier | Univ. Goettingen |
| EC | GUA_07 | NAP | Guacamayos | -0.643 | -77.837 | 1940 | 0.04 | Q | 20 x 20 | P | 3142 | 17.2 | 3 | 2005 2008 2011 | 3, 3 | 5 | 675 | 48.2 | 19 | -- | Homeier | Univ. Goettingen |
| EC | GUA_08 | NAP | Guacamayos | -0.637 | -77.84 | 1995 | 0.04 | Q | 20 x 20 | P | 3182 | 16.6 | 3 | 2005 2008 2011 | 3, 3 | 5 | 575 | 25.25 | 14 | -- | Homeier | Univ. Goettingen |
| EC | GUA_27 | NAP | Guacamayos | -0.64 | -77.839 | 1990 | 0.04 | Q | 20 x 20 | P | 3182 | 16.6 | 1 | 2006 | -- | 5 | 775 | 34.5 | 22 | -- | Homeier | Univ. Goettingen |
| EC | GUA_52 | NAP | Guacamayos | -0.639 | -77.84 | 1980 | 0.04 | Q | 20 x 20 | P | 3182 | 16.6 | 1 | 2006 | -- | 5 | 925 | 29.25 | 14 | -- | Homeier | Univ. Goettingen |
| EC | GUA_53 | NAP | Guacamayos | -0.64 | -77.839 | 1980 | 0.04 | Q | 20 x 20 | P | 3182 | 16.6 | 1 | 2006 | -- | 5 | 1025 | 35 | 26 | -- | Homeier | Univ. Goettingen |
| EC | GUA_54 | NAP | Guacamayos | -0.637 | -77.84 | 2000 | 0.04 | Q | 20 x 20 | P | 3182 | 16.6 | 1 | 2006 | -- | 5 | 800 | 49.75 | 25 | -- | Homeier | Univ. Goettingen |
| EC | GUA_55 | NAP | Guacamayos | -0.637 | -77.84 | 2000 | 0.04 | Q | 20 x 20 | P | 3182 | 16.6 | 1 | 2006 | -- | 5 | 825 | 46.75 | 21 | -- | Homeier | Univ. Goettingen |
| EC | HAM_42 | NAP | Hakuna Matata | -0.898 | -77.845 | 970 | 0.04 | Q | 20 x 20 | P | 4456 | 21.5 | 3 | 2006 2008 2011 | 2, 3 | 5 | 1050 | 57 | 17 | -- | Homeier | Univ. Goettingen |
| EC | HAM_43 | NAP | Hakuna Matata | -0.898 | -77.845 | 960 | 0.04 | Q | 20 x 20 | P | 4456 | 21.5 | 3 | 2006 2008 2011 | 2, 3 | 5 | 950 | 48.75 | 27 | -- | Homeier | Univ. Goettingen |
| EC | HAM_44 | NAP | Hakuna Matata | -0.897 | -77.845 | 1000 | 0.04 | Q | 20 x 20 | P | 4456 | 21.5 | 1 | 2006 | -- | 5 | 850 | 39.25 | 21 | -- | Homeier | Univ. Goettingen |
| EC | HAM_45 | NAP | Hakuna Matata | -0.897 | -77.846 | 1050 | 0.04 | Q | 20 x 20 | P | 4456 | 21.5 | 1 | 2006 | -- | 5 | 900 | 35.25 | 27 | -- | Homeier | Univ. Goettingen |
| EC | HAM_46 | NAP | Hakuna Matata | -0.897 | -77.845 | 1020 | 0.04 | Q | 20 x 20 | P | 4456 | 21.5 | 1 | 2006 | -- | 5 | 1000 | 44.75 | 27 | -- | Homeier | Univ. Goettingen |
| EC | HAM_51 | NAP | Hakuna Matata | -0.897 | -77.846 | 1080 | 0.04 | Q | 20 x 20 | P | 4456 | 21.5 | 1 | 2006 | -- | 5 | 850 | 51 | 28 | -- | Homeier | Univ. Goettingen |
| EC | OYA_81 | NAP | Oyacachi | -0.301 | -78.134 | 3980 | 0.04 | Q | 20 x 20 | P | 950 | 5.9 | 2 | 2009 2011 | 2 | 5 | 1000 | 33.07 | 2 | -- | Homeier | Univ. Goettingen |
| EC | OYA_82 | NAP | Oyacachi | -0.258 | -78.093 | 3930 | 0.04 | Q | 20 x 20 | P | 974 | 6.4 | 2 | 2009 2011 | 2 | 5 | 1125 | 37.30 | 3 | -- | Homeier | Univ. Goettingen |
| EC | OYA_83 | NAP | Oyacachi | -0.249 | -78.098 | 3920 | 0.04 | Q | 20 x 20 | P | 1017 | 6.3 | 2 | 2009 2011 | 2 | 5 | 550 | 18.35 | 2 | -- | Homeier | Univ. Goettingen |
| EC | OYA_84 | NAP | Oyacachi | -0.187 | -78.136 | 3660 | 0.04 | Q | 20 x 20 | P | 848 | 7.4 | 2 | 2009 2011 | 2 | 5 | 900 | 27.03 | 4 | -- | Homeier | Univ. Goettingen |
| EC | OYA_85 | NAP | Oyacachi | -0.195 | -78.129 | 3630 | 0.04 | Q | 20 x 20 | P | 862 | 7.5 | 2 | 2009 2011 | 2 | 5 | 875 | 30.77 | 2 | -- | Homeier | Univ. Goettingen |
| EC | OYA_86 | NAP | Oyacachi | -0.195 | -78.129 | 3640 | 0.04 | Q | 20 x 20 | P | 862 | 7.5 | 2 | 2009 2011 | 2 | 5 | 425 | 7.78 | 4 | -- | Homeier | Univ. Goettingen |
| EC | RHO_63 | NAP | Rio Hollin | -0.676 | -77.748 | 1190 | 0.04 | Q | 20 x 20 | P | 2988 | 20.2 | 1 | 2007 | -- | 5 | 1100 | 49.75 | 15 | -- | Homeier | Univ. Goettingen |
| EC | RHO_64 | NAP | Rio Hollin | -0.676 | -77.748 | 1195 | 0.04 | Q | 20 x 20 | P | 2988 | 20.2 | 3 | 2007 2008 2011 | 1, 3 | 5 | 1150 | 35.25 | 13 | -- | Homeier | Univ. Goettingen |
| EC | RHO_65 | NAP | Rio Hollin | -0.676 | -77.747 | 1210 | 0.04 | Q | 20 x 20 | P | 2988 | 20.2 | 3 | 2007 2008 2011 | 1, 3 | 5 | 950 | 76.5 | 24 | -- | Homeier | Univ. Goettingen |
| EC | RHO_66 | NAP | Rio Hollin | -0.68 | -77.747 | 1165 | 0.04 | Q | 20 x 20 | P | 2988 | 20.2 | 3 | 2007 2008 2011 | 1, 3 | 5 | 1150 | 45.5 | 19 | -- | Homeier | Univ. Goettingen |
| EC | RHO_67 | NAP | Rio Hollin | -0.68 | -77.748 | 1180 | 0.04 | Q | 20 x 20 | P | 2988 | 20.2 | 1 | 2007 | -- | 5 | 975 | 38.5 | 19 | -- | Homeier | Univ. Goettingen |
| EC | SUM_09 | NAP | Sumaco | -0.591 | -77.588 | 1920 | 0.04 | Q | 20 x 20 | P | 4173 | 16.1 | 3 | 2005 2008 2011 | 3, 3 | 5 | 350 | 31.25 | 6 | -- | Homeier | Univ. Goettingen |
| EC | SUM_10 | NAP | Sumaco | -0.591 | -77.588 | 1940 | 0.04 | Q | 20 x 20 | P | 4173 | 16.1 | 3 | 2005 2008 2011 | 3, 3 | 5 | 525 | 63.5 | 13 | -- | Homeier | Univ. Goettingen |
| EC | SUM_11 | NAP | Sumaco | -0.59 | -77.589 | 1950 | 0.04 | Q | 20 x 20 | P | 4173 | 16.1 | 3 | 2005 2008 2011 | 3, 3 | 5 | 475 | 50 | 13 | -- | Homeier | Univ. Goettingen |
| EC | SUM_12 | NAP | Sumaco | -0.584 | -77.591 | 2015 | 0.04 | Q | 20 x 20 | P | 4173 | 16.1 | 1 | 2005 | -- | 5 | 800 | 140.3 | 20 | -- | Homeier | Univ. Goettingen |
| EC | SUM_13 | NAP | Sumaco | -0.585 | -77.591 | 2000 | 0.04 | Q | 20 x 20 | P | 4173 | 16.1 | 1 | 2005 | -- | 5 | 625 | 70.25 | 18 | -- | Homeier | Univ. Goettingen |
| EC | SUM_14 | NAP | Sumaco | -0.619 | -77.596 | 1580 | 0.04 | Q | 20 x 20 | P | 3829 | 17.6 | 1 | 2005 | -- | 5 | 475 | 35.5 | 16 | -- | Homeier | Univ. Goettingen |
| EC | SUM_15 | NAP | Sumaco | -0.619 | -77.597 | 1590 | 0.04 | Q | 20 x 20 | P | 3829 | 17.6 | 3 | 2005 2008 2011 | 3, 3 | 5 | 550 | 55.75 | 16 | -- | Homeier | Univ. Goettingen |
| EC | SUM_16 | NAP | Sumaco | -0.628 | -77.587 | 1610 | 0.04 | Q | 20 x 20 | P | 3927 | 17.9 | 3 | 2005 2008 2011 | 3, 3 | 5 | 625 | 50.25 | 16 | -- | Homeier | Univ. Goettingen |
| EC | SUM_17 | NAP | Sumaco | -0.628 | -77.588 | 1610 | 0.04 | Q | 20 x 20 | P | 3927 | 17.9 | 1 | 2005 | -- | 5 | 625 | 64.75 | 15 | -- | Homeier | Univ. Goettingen |
| EC | SUM_18 | NAP | Sumaco | -0.635 | -77.587 | 1590 | 0.04 | Q | 20 x 20 | P | 3918 | 18.1 | 3 | 2005 2008 2011 | 3, 3 | 5 | 525 | 67.75 | 15 | -- | Homeier | Univ. Goettingen |
| EC | SUM_19 | NAP | Sumaco | -0.635 | -77.587 | 1590 | 0.04 | Q | 20 x 20 | P | 3918 | 18.1 | 1 | 2005 | -- | 5 | 600 | 57.25 | 20 | -- | Homeier | Univ. Goettingen |
| EC | SUM_56 | NAP | Sumaco | -0.592 | -77.588 | 1931 | 0.04 | Q | 20 x 20 | P | 4110 | 16.6 | 1 | 2006 | -- | 5 | 875 | 48.5 | 25 | -- | Homeier | Univ. Goettingen |
| EC | SUM_57 | NAP | Sumaco | -0.584 | -77.591 | 2000 | 0.04 | Q | 20 x 20 | P | 4173 | 16.1 | 1 | 2006 | -- | 5 | 725 | 43.5 | 11 | -- | Homeier | Univ. Goettingen |
| EC | SUM_58 | NAP | Sumaco | -0.619 | -77.596 | 1580 | 0.04 | Q | 20 x 20 | P | 3829 | 17.6 | 1 | 2006 | -- | 5 | 675 | 38.75 | 15 | -- | Homeier | Univ. Goettingen |
| EC | SUM_59 | NAP | Sumaco | -0.629 | -77.587 | 1600 | 0.04 | Q | 20 x 20 | P | 3927 | 17.9 | 1 | 2006 | -- | 5 | 725 | 71 | 21 | -- | Homeier | Univ. Goettingen |
| EC | YAY_01 | NAP | Yanayacu | -0.602 | -77.883 | 2085 | 0.04 | Q | 20 x 20 | P | 2387 | 15.8 | 3 | 2005 2008 2011 | 3, 3 | 5 | 575 | 35 | 12 | -- | Homeier | Univ. Goettingen |
| EC | YAY_02 | NAP | Yanayacu | -0.602 | -77.879 | 2080 | 0.04 | Q | 20 x 20 | P | 2387 | 15.8 | 1 | 2005 | -- | 5 | 725 | 44.5 | 14 | -- | Homeier | Univ. Goettingen |
| EC | YAY_03 | NAP | Yanayacu | -0.605 | -77.877 | 2055 | 0.04 | Q | 20 x 20 | P | 2387 | 15.8 | 3 | 2005 2008 2011 | 3, 3 | 5 | 800 | 57.5 | 16 | -- | Homeier | Univ. Goettingen |
| EC | YAY_04 | NAP | Yanayacu | -0.606 | -77.878 | 2070 | 0.04 | Q | 20 x 20 | P | 2387 | 15.8 | 1 | 2005 | -- | 5 | 625 | 38.5 | 13 | -- | Homeier | Univ. Goettingen |
| EC | YAY_05 | NAP | Yanayacu | -0.601 | -77.884 | 2080 | 0.04 | Q | 20 x 20 | P | 2677 | 15.6 | 1 | 2005 | -- | 5 | 800 | 63.75 | 19 | -- | Homeier | Univ. Goettingen |
| EC | YAY_87 | NAP | Yanayacu | -0.584 | -77.898 | 2420 | 0.04 | Q | 20 x 20 | P | 2752 | 14.9 | 2 | 2011 2012 | 1 | 5 | 675 | 35.82 | 15 | -- | Homeier | Univ. Goettingen |
| EC | YAY_88 | NAP | Yanayacu | -0.584 | -77.898 | 2410 | 0.04 | Q | 20 x 20 | P | 2752 | 14.9 | 2 | 2011 2012 | 1 | 5 | 925 | 26.71 | 34 | -- | Homeier | Univ. Goettingen |
| EC | YAY_89 | NAP | Yanayacu | -0.593 | -77.900 | 2410 | 0.04 | Q | 20 x 20 | P | 2856 | 14.8 | 2 | 2011 2012 | 1 | 5 | 600 | 38.41 | 13 | -- | Homeier | Univ. Goettingen |
| EC | BOM_LS1 | ZCH | Bombuscaro | -4.12 | -78.97 | 1020 | 0.04 | Q | 20 x 20 | P | 1046 | 20.9 | 3 | 2008 2009 2016 | 1, 7 | 5 | 650 | 38.60 | 19 | -- | Homeier | Univ. Goettingen |
| EC | BOM_LS2 | ZCH | Bombuscaro | -4.12 | -78.971 | 1026 | 0.04 | Q | 20 x 20 | P | 1046 | 20.9 | 3 | 2008 2009 2016 | 1, 7 | 5 | 950 | 57.96 | 25 | -- | Homeier | Univ. Goettingen |
| EC | BOM_LS3 | ZCH | Bombuscaro | -4.12 | -78.971 | 1046 | 0.04 | Q | 20 x 20 | P | 1046 | 20.9 | 3 | 2008 2009 2016 | 1, 7 | 5 | 875 | 54.46 | 20 | -- | Homeier | Univ. Goettingen |
| EC | BOM_LS4 | ZCH | Bombuscaro | -4.125 | -78.977 | 1054 | 0.04 | Q | 20 x 20 | P | 1111 | 19.9 | 3 | 2008 2009 2016 | 1, 7 | 5 | 625 | 32.22 | 19 | -- | Homeier | Univ. Goettingen |
| EC | BOM_LS5 | ZCH | Bombuscaro | -4.125 | -78.978 | 1056 | 0.04 | Q | 20 x 20 | P | 1111 | 19.9 | 3 | 2008 2009 2016 | 1, 7 | 5 | 575 | 39.95 | 13 | -- | Homeier | Univ. Goettingen |
| EC | BOM_LS6 | ZCH | Bombuscaro | -4.125 | -78.978 | 1044 | 0.04 | Q | 20 x 20 | P | 1111 | 19.9 | 3 | 2008 2009 2016 | 1, 7 | 5 | 625 | 58.21 | 12 | -- | Homeier | Univ. Goettingen |
| EC | BOM_MS1 | ZCH | Bombuscaro | -4.118 | -78.97 | 1132 | 0.04 | Q | 20 x 20 | P | 1046 | 20.9 | 3 | 2008 2009 2016 | 1, 7 | 5 | 900 | 31.16 | 19 | -- | Homeier | Univ. Goettingen |
| EC | BOM_MS2 | ZCH | Bombuscaro | -4.121 | -78.973 | 1069 | 0.04 | Q | 20 x 20 | P | 1046 | 20.9 | 3 | 2008 2009 2016 | 1, 7 | 5 | 675 | 32.61 | 20 | -- | Homeier | Univ. Goettingen |
| EC | BOM_MS3 | ZCH | Bombuscaro | -4.123 | -78.975 | 1049 | 0.04 | Q | 20 x 20 | P | 1046 | 20.9 | 3 | 2008 2009 2016 | 1, 7 | 5 | 875 | 56.57 | 18 | -- | Homeier | Univ. Goettingen |
| EC | BOM_MS4 | ZCH | Bombuscaro | -4.124 | -78.978 | 1140 | 0.04 | Q | 20 x 20 | P | 1111 | 19.9 | 3 | 2008 2009 2016 | 1, 7 | 5 | 550 | 131.10 | 17 | -- | Homeier | Univ. Goettingen |
| EC | BOM_MS5 | ZCH | Bombuscaro | -4.125 | -78.979 | 1170 | 0.04 | Q | 20 x 20 | P | 1111 | 19.9 | 3 | 2008 2009 2016 | 1, 7 | 5 | 725 | 50.12 | 20 | -- | Homeier | Univ. Goettingen |
| EC | BOM_MS6 | ZCH | Bombuscaro | -4.126 | -78.979 | 1129 | 0.04 | Q | 20 x 20 | P | 957 | 20.7 | 3 | 2008 2009 2016 | 1, 7 | 5 | 625 | 56.81 | 19 | -- | Homeier | Univ. Goettingen |
| EC | BOM_US1 | ZCH | Bombuscaro | -4.117 | -78.969 | 1075 | 0.04 | Q | 20 x 20 | P | 1046 | 20.9 | 3 | 2008 2009 2016 | 1, 7 | 5 | 1100 | 38.78 | 10 | -- | Homeier | Univ. Goettingen |
| EC | BOM_US2 | ZCH | Bombuscaro | -4.118 | -78.969 | 1066 | 0.04 | Q | 20 x 20 | P | 1046 | 20.9 | 3 | 2008 2009 2016 | 1, 7 | 5 | 875 | 24.68 | 10 | -- | Homeier | Univ. Goettingen |
| EC | BOM_US3 | ZCH | Bombuscaro | -4.117 | -78.969 | 1072 | 0.04 | Q | 20 x 20 | P | 1046 | 20.9 | 3 | 2008 2009 2016 | 1, 7 | 5 | 725 | 23.74 | 10 | -- | Homeier | Univ. Goettingen |
| EC | BOM_US4 | ZCH | Bombuscaro | -4.124 | -78.98 | 1268 | 0.04 | Q | 20 x 20 | P | 1111 | 19.9 | 3 | 2008 2009 2016 | 1, 7 | 5 | 650 | 30.45 | 16 | -- | Homeier | Univ. Goettingen |
| EC | BOM_US5 | ZCH | Bombuscaro | -4.124 | -78.98 | 1257 | 0.04 | Q | 20 x 20 | P | 1111 | 19.9 | 3 | 2008 2009 2016 | 1, 7 | 5 | 825 | 41.83 | 21 | -- | Homeier | Univ. Goettingen |
| EC | BOM_US6 | ZCH | Bombuscaro | -4.125 | -78.98 | 1266 | 0.04 | Q | 20 x 20 | P | 1111 | 19.9 | 3 | 2008 2009 2016 | 1, 7 | 5 | 700 | 29.19 | 18 | -- | Homeier | Univ. Goettingen |
| EC | ECSF_01 | ZCH | San Francisco | -3.977 | -79.079 | 1850 | 0.04 | Q | 20 x 20 | P | 1003 | 16.2 | 3 | 2000 2001 2002 | 1.1 | 5 | 850 | 25.75 | 17 | -- | Homeier | Univ. Goettingen |
| EC | ECSF_02 | ZCH | San Francisco | -3.979 | -79.077 | 2090 | 0.04 | Q | 20 x 20 | P | 1003 | 16.2 | 3 | 2000 2001 2002 | 1.1 | 5 | 1000 | 29.50 | 18 | -- | Homeier | Univ. Goettingen |
| EC | ECSF_03 | ZCH | San Francisco | -3.982 | -79.076 | 2180 | 0.04 | Q | 20 x 20 | P | 1003 | 16.2 | 3 | 2000 2001 2002 | 1.1 | 5 | 850 | 23.50 | 14 | -- | Homeier | Univ. Goettingen |
| EC | ECSF_04 | ZCH | San Francisco | -3.989 | -79.075 | 2370 | 0.04 | Q | 20 x 20 | P | 1153 | 14.6 | 3 | 2000 2001 2002 | 1.1 | 5 | 400 | 5.50 | 7 | -- | Homeier | Univ. Goettingen |
| EC | ECSF_05 | ZCH | San Francisco | -3.976 | -79.079 | 1870 | 0.04 | Q | 20 x 20 | P | 1003 | 16.2 | 3 | 2000 2001 2002 | 1.1 | 5 | 400 | 17.00 | 12 | -- | Homeier | Univ. Goettingen |
| EC | ECSF_06 | ZCH | San Francisco | -3.992 | -79.076 | 2450 | 0.04 | Q | 20 x 20 | P | 1110 | 14.6 | 3 | 2000 2001 2002 | 1.1 | 5 | 325 | 7.50 | 2 | -- | Homeier | Univ. Goettingen |
| EC | ECSF_07 | ZCH | San Francisco | -3.977 | -79.078 | 1980 | 0.04 | Q | 20 x 20 | P | 1003 | 16.2 | 3 | 2000 2001 2002 | 1.1 | 5 | 875 | 17.75 | 15 | -- | Homeier | Univ. Goettingen |
| EC | ECSF_08 | ZCH | San Francisco | -3.985 | -79.076 | 2250 | 0.04 | Q | 20 x 20 | P | 1030 | 15.3 | 3 | 2000 2001 2002 | 1.1 | 5 | 625 | 14.00 | 10 | -- | Homeier | Univ. Goettingen |
| EC | ECSF_09 | ZCH | San Francisco | -3.981 | -79.077 | 2150 | 0.04 | Q | 20 x 20 | P | 1003 | 16.2 | 1 | 2000 | -- | 5 | 1050 | 23.43 | 12 | -- | Homeier | Univ. Goettingen |
| EC | ECSF_11 | ZCH | San Francisco | -3.978 | -79.078 | 2000 | 0.04 | Q | 20 x 20 | P | 1003 | 16.2 | 3 | 2000 2001 2002 | 1.1 | 5 | 875 | 28.90 | 21 | -- | Homeier | Univ. Goettingen |
| EC | ECSF_12 | ZCH | San Francisco | -3.979 | -79.079 | 1960 | 0.04 | Q | 20 x 20 | P | 1003 | 16.2 | 3 | 2000 2001 2002 | 1.1 | 5 | 750 | 47.57 | 22 | -- | Homeier | Univ. Goettingen |
| EC | ECSF_14 | ZCH | San Francisco | -3.983 | -79.076 | 2210 | 0.04 | Q | 20 x 20 | P | 1003 | 16.2 | 1 | 2000 | 1 | 5 | 700 | 18.00 | 13 | -- | Homeier | Univ. Goettingen |
| EC | ECSF_15 | ZCH | San Francisco | -3.977 | -79.079 | 1910 | 0.04 | Q | 20 x 20 | P | 1003 | 16.2 | 1 | 2000 | 1 | 5 | 550 | 26.00 | 13 | -- | Homeier | Univ. Goettingen |
| EC | SFM_LS1 | ZCH | San Francisco | -3.98 | -79.078 | 2039 | 0.04 | Q | 20 x 20 | P | 1003 | 16.2 | 3 | 2008 2009 2016 | 1, 7 | 5 | 650 | 65.38 | 20 | -- | Homeier | Univ. Goettingen |
| EC | SFM_LS2 | ZCH | San Francisco | -3.979 | -79.078 | 1993 | 0.04 | Q | 20 x 20 | P | 1003 | 16.2 | 3 | 2008 2009 2016 | 1, 7 | 5 | 400 | 57.95 | 12 | -- | Homeier | Univ. Goettingen |
| EC | SFM_LS3 | ZCH | San Francisco | -3.979 | -79.078 | 2020 | 0.04 | Q | 20 x 20 | P | 1003 | 16.2 | 3 | 2008 2009 2016 | 1, 7 | 5 | 550 | 63.07 | 14 | -- | Homeier | Univ. Goettingen |
| EC | SFM_LS4 | ZCH | San Francisco | -3.977 | -79.073 | 1913 | 0.04 | Q | 20 x 20 | P | 1036 | 15.6 | 3 | 2008 2009 2016 | 1, 7 | 5 | 925 | 13.74 | 12 | -- | Homeier | Univ. Goettingen |
| EC | SFM_LS5 | ZCH | San Francisco | -3.978 | -79.073 | 1954 | 0.04 | Q | 20 x 20 | P | 1036 | 15.6 | 3 | 2008 2009 2016 | 1, 7 | 5 | 825 | 43.23 | 25 | -- | Homeier | Univ. Goettingen |
| EC | SFM_LS6 | ZCH | San Francisco | -3.978 | -79.073 | 1933 | 0.04 | Q | 20 x 20 | P | 1036 | 15.6 | 3 | 2008 2009 2016 | 1, 7 | 5 | 475 | 80.39 | 25 | -- | Homeier | Univ. Goettingen |
| EC | SFM_MS1 | ZCH | San Francisco | -3.98 | -79.078 | 2034 | 0.04 | Q | 20 x 20 | P | 1003 | 16.2 | 3 | 2008 2009 2016 | 1, 7 | 5 | 775 | 46.45 | 21 | -- | Homeier | Univ. Goettingen |
| EC | SFM_MS2 | ZCH | San Francisco | -3.977 | -79.075 | 1950 | 0.04 | Q | 20 x 20 | P | 1036 | 15.6 | 3 | 2008 2009 2016 | 1, 7 | 5 | 825 | 48.78 | 27 | -- | Homeier | Univ. Goettingen |
| EC | SFM_MS3 | ZCH | San Francisco | -3.978 | -79.075 | 2027 | 0.04 | Q | 20 x 20 | P | 1036 | 15.6 | 3 | 2008 2009 2016 | 1, 7 | 5 | 775 | 66.02 | 18 | -- | Homeier | Univ. Goettingen |
| EC | SFM_MS4 | ZCH | San Francisco | -3.978 | -79.074 | 1927 | 0.04 | Q | 20 x 20 | P | 1036 | 15.6 | 3 | 2008 2009 2016 | 1, 7 | 5 | 1100 | 47.25 | 26 | -- | Homeier | Univ. Goettingen |
| EC | SFM_MS5 | ZCH | San Francisco | -3.978 | -79.074 | 1971 | 0.04 | Q | 20 x 20 | P | 1036 | 15.6 | 3 | 2008 2009 2016 | 1, 7 | 5 | 925 | 44.68 | 20 | -- | Homeier | Univ. Goettingen |
| EC | SFM_MS6 | ZCH | San Francisco | -3.979 | -79.074 | 2020 | 0.04 | Q | 20 x 20 | P | 1036 | 15.6 | 3 | 2008 2009 2016 | 1, 7 | 5 | 700 | 44.25 | 24 | -- | Homeier | Univ. Goettingen |
| EC | SFM_US1 | ZCH | San Francisco | -3.978 | -79.077 | 2002 | 0.04 | Q | 20 x 20 | P | 1003 | 16.2 | 3 | 2008 2009 2016 | 1, 7 | 5 | 775 | 24.17 | 14 | -- | Homeier | Univ. Goettingen |
| EC | SFM_US2 | ZCH | San Francisco | -3.978 | -79.077 | 2026 | 0.04 | Q | 20 x 20 | P | 1003 | 16.2 | 3 | 2008 2009 2016 | 1, 7 | 5 | 1250 | 45.92 | 22 | -- | Homeier | Univ. Goettingen |
| EC | SFM_US3 | ZCH | San Francisco | -3.981 | -79.078 | 2089 | 0.04 | Q | 20 x 20 | P | 1003 | 16.2 | 3 | 2008 2009 2016 | 1, 7 | 5 | 1000 | 40.73 | 18 | -- | Homeier | Univ. Goettingen |
| EC | SFM_US4 | ZCH | San Francisco | -3.979 | -79.075 | 2063 | 0.04 | Q | 20 x 20 | P | 1036 | 15.6 | 3 | 2008 2009 2016 | 1, 7 | 5 | 1200 | 44.96 | 23 | -- | Homeier | Univ. Goettingen |
| EC | SFM_US5 | ZCH | San Francisco | -3.979 | -79.075 | 2054 | 0.04 | Q | 20 x 20 | P | 1036 | 15.6 | 3 | 2008 2009 2016 | 1, 7 | 5 | 850 | 32.11 | 24 | -- | Homeier | Univ. Goettingen |
| EC | SFM_US6 | ZCH | San Francisco | -3.978 | -79.075 | 2039 | 0.04 | Q | 20 x 20 | P | 1036 | 15.6 | 3 | 2008 2009 2016 | 1, 7 | 5 | 1025 | 47.82 | 24 | -- | Homeier | Univ. Goettingen |
| CO | Angelópolis | ANT | Cordillera Central | 6.15 | -75.70 | 2027 | 1 | Q | 100x100 | P | 2197 | 16.2 | 3 | 2006 2009 2014 | 4 | 1 | 868 | 21.79 | 94 | NA | Duque | Cs. Forestales. Medellin. UNAL |
| CO | Anorí | ANT | Cordillera Central | 6.99 | -75.14 | 1740 | 1 | Q | 100x100 | P | 4354 | 17.5 | 3 | 2006 2009 2014 | 4 | 1 | 918 | 25.79 | 87 | NA | Duque | Cs. Forestales. Medellin. UNAL |
| CO | Belmira | ANT | Cordillera Central | 6.61 | -75.65 | 2928 | 1 | Q | 100x100 | P | 2339 | 11.5 | 3 | 2006 2009 2014 | 4 | 1 | 541 | 21.35 | 31 | NA | Duque | Cs. Forestales. Medellin. UNAL |
| CO | Caicedo | ANT | Cordillera Central | 6.38 | -76.03 | 2646 | 1 | Q | 100x100 | P | 2124 | 13.2 | 2 | 2009 2014 | 5 | 1 | 1245 | 35.39 | 91 | NA | Duque | Cs. Forestales. Medellin. UNAL |
| CO | Carepa | ANT | Urabá | 7.78 | -76.67 | 59 | 1 | Q | 100x100 | P | 2518 | 26 | 3 | 2009 2013 2014 | 3 | 1 | 372 | 26.34 | 84 | NA | Duque | Cs. Forestales. Medellin. UNAL |
| CO | Caucasia | ANT | Bajo Cauca | 8.13 | -74.94 | 128 | 1 | Q | 100x100 | P | 2349 | 26.5 | 2 | 2009 2014 | 5 | 1 | 513 | 24.33 | 58 | NA | Duque | Cs. Forestales. Medellin. UNAL |
| CO | El Bagre | ANT | Bajo Cauca | 7.66 | -74.82 | 166 | 1 | Q | 100x100 | P | 3025 | 26.6 | 2 | 2009 2014 | 5 | 1 | 505 | 22.30 | 103 | NA | Duque | Cs. Forestales. Medellin. UNAL |
| CO | Jardín | ANT | Cordillera | 5.50 | -75.90 | 2527 | 1 | Q | 100x100 | P | 2434 | 15.2 | 2 | 2009 2013 | 4 | 1 | 937 | 40.57 | 55 | NA | Duque | Cs. Forestales. Medellin. UNAL |
| CO | Maceo | ANT | Magdalena Medio | 6.46 | -74.79 | 928 | 1 | Q | 100x100 | P | 3145 | 22.2 | 2 | 2009 2013 | 5 | 1 | 821 | 31.03 | 103 | NA | Duque | Cs. Forestales. Medellin. UNAL |
| CO | Necoclí | ANT | Urabá | 8.51 | -76.66 | 41 | 1 | Q | 100x100 | P | 2007 | 26.3 | 2 | 2009 2014 | 5 | 1 | 565 | 24.80 | 77 | NA | Duque | Cs. Forestales. Medellin. UNAL |
| CO | Porce | ANT | Cordillera Central | 6.78 | -75.08 | 977 | 1 | Q | 100x100 | P | 2108 | 21.9 | 2 | 2008 2013 | 5 | 1 | 834 | 25.34 | 57 | NA | Duque | Cs. Forestales. Medellin. UNAL |
| CO | Puerto Triunfo | ANT | Magdalena Medio | 6.01 | -74.61 | 167 | 1 | Q | 100x100 | P | 2783 | 27 | 2 | 2009 2014 | 5 | 1 | 394 | 22.61 | 56 | NA | Duque | Cs. Forestales. Medellin. UNAL |
| CO | Sapzurro | ANT | Sapzurro | 8.65 | -77.36 | 171 | 1 | Q | 100x100 | P | 2877 | 25.8 | 2 | 2010 2013 | 3 | 1 | 612 | 28.93 | 70 | NA | Duque | Cs. Forestales. Medellin. UNAL |
| CO | Segovia | ANT | Cordillera Central | 7.11 | -74.73 | 691 | 1 | Q | 100x100 | P | 3803 | 23.2 | 2 | 2008 2013 | 5 | 1 | 624 | 24.55 | 120 | NA | Duque | Cs. Forestales. Medellin. UNAL |
| CO | Támesis | ANT | Valle del rio Cauca | 5.78 | -75.67 | 586 | 1 | Q | 100x100 | P | 1854 | 23.5 | 2 | 2009 2014 | 5 | 1 | 445 | 18.03 | 39 | NA | Duque | Cs. Forestales. Medellin. UNAL |
| CO | Ventanas | ANT | Ventanas | 7.08 | -75.48 | 2056 | 1 | Q | 100x100 | P | 3868 | 16.1 | 2 | 2009 2013 | 4 | 1 | 939 | 23.77 | 82 | NA | Duque | Cs. Forestales. Medellin. UNAL |
| VE | Mu2300 | MER | La Mucuy | 8.63 | -71.04 | 2300 | 0.36 | Q | 60 x 60 | P | 1043 | 14 | 1 | 2017 | -- | 5 | 900 | 44.70 | 37 | -- | Llambi | ICAE-ULA |
| VE | Mu2500 | MER | La Mucuy | 8.63 | -71.04 | 2500 | 0.36 | Q | 60 x 60 | P | 1043 | 14 | 1 | 2017 | -- | 5 | 922 | 43.10 | 39 | -- | Llambi | ICAE-ULA |
| VE | Mu2700 | MER | La Mucuy | 8.63 | -71.03 | 2700 | 0.36 | Q | 60 x 60 | P | 1016 | 12.9 | 1 | 2017 | -- | 5 | 897 | 40.80 | 38 | -- | Llambi | ICAE-ULA |
