# Supplementary material for: Elevation and latitude drives structure and tree species composition in Andean forests: Results from a large-scale plot network
Source: PLoS One. 2020 Apr 20;15(4):e0231553. doi: 10.1371/journal.pone.0231553 (PMC7170706; doi:10.1371/journal.pone.0231553)
Supplement: S1 Appendix — Summary of Generalized Linear Models (GLM) for stem density, basal area and species richness considering elevation, latitude and plot size as explanatory variables. SE = Standard error. VE = Variance explained ((Null Deviance—Residual Deviance)/Null Deviance x 100). (*) Only 1-ha plots were included in the model considering Tropical and Subtropical Andes. (DOCX) [file pone.0231553.s002.docx]

**Appendix 1.** **Generalized Linear Models.** Summary of Generalized Linear Models (GLM) for stem density, basal area and species richness, considering elevation, latitude and plot size as explanatory variables. SE = Standard error. VE = Variance explained ((Null Deviance – Residual Deviance)/Null Deviance x 100). (*) Only 1-ha plots were included in the model considering Tropical and Subtropical Andes.

| **Response variable** | **Explanatory variable** | **Estimate** | **SE** | **z-value** | **P-value** | **Null Deviance** | **Residual Deviance** | **VE** |
| --- | --- | --- | --- | --- | --- | --- | --- | --- |
| Stem density | Intercept | 5.7 | 9.1e-02 | 62.9 | 0.0001 | 22852 | 12969 | 43.2 |
|  | Elevation | 5.6e-04 | 1.1e-04 | 5.3 | 0.0001 |  |  |  |
|  | Elevation^2^ | -1.1e-07 | 2.8e-08 | -3.9 | 0.0001 |  |  |  |
|  | Latitude | 7.1e-03 | 1.0e-02 | 6.9 | 0.0001 |  |  |  |
|  | Latitude^2^ | -2.8e-04 | 3.6e-04 | -7.8 | 0.0001 |  |  |  |
|  | Plot size | -7.5e-02 | 5.2e-02 | -1.4 | 0.15 |  |  |  |
| Basal area | Intercept | 3.1 | 1.2e-01 | 26.2 | 0.0001 | 31175 | 28225 | 9.5 |
|  | Elevation | 3.6e-04 | 1.4e-04 | 2.5 | 0.01 |  |  |  |
|  | Elevation^2^ | -8.3e-08 | 3.9e-08 | -2.1 | 0.03 |  |  |  |
|  | Latitude | 4.9e-03 | 3.6e-03 | 1.4 | 0.17 |  |  |  |
|  | Plot size | -2.3e-01 | 7.6e-02 | -2.9 | 0.003 |  |  |  |
| Species richness | Intercept | 3.1 | 8.2e-02 | 37.4 | 0.0001 | 2754 | 840 | 69.5 |
|  | Elevation | 4.5e-04 | 9.8e-05 | 4.6 | 0.0001 |  |  |  |
|  | Elevation^2^ | -2e-07 | 2.9e-08 | -7.0 | 0.0001 |  |  |  |
|  | Latitude | 5.9e-02 | 1.1e-02 | 5.3 | 0.0001 |  |  |  |
|  | Latitude^2^ | -3.9e-03 | 4e-04 | -9.9 | 0.0001 |  |  |  |
|  | Plot size | 9.3e-01 | 6.8e-02 | 13.7 | 0.0001 |  |  |  |
| Species richness (*) | Elevation | 3.1e-04 | 1.5e-04 | 2.0 | 0.05 | 1453 | 650 | 55.3 |
|  | Elevation^2^ | -1.6e-07 | 4.6e-08 | -3.6 | 0.001 |  |  |  |
|  | Tropical Andes | 4.2 | 1.2e-01 | 27.9 | 0.0001 |  |  |  |
|  | Subtropical Andes | 3.4 | 1.1e-01 | 38.8 | 0.0001 |  |  |  |
