# Supplementary material for: Elevation and latitude drives structure and tree species composition in Andean forests: Results from a large-scale plot network
Source: PLoS One. 2020 Apr 20;15(4):e0231553. doi: 10.1371/journal.pone.0231553 (PMC7170706; doi:10.1371/journal.pone.0231553)
Supplement: S2 Appendix — Abundance of tree species per country, considering individuals ≥10 cm DBH listed by botanical family. Species and family names were actualized with TROPICOS in September 2019 (http://www.tropicos.org).aCountry codes are AR, Argentina; BO, Bolivia; PE, Peru; EC, Ecuador; CO, Colombia; and VE, Venezuela. (DOCX) [file pone.0231553.s003.docx]

**Appendix 2.** **Abundance of tree species per country.** Abundance of tree species per country, considering individuals ≥10 cm DBH listed by botanical family. Species and family names were actualized with TROPICOS in September 2019 (<http://www.tropicos.org>). ^a^Country codes are AR, Argentina; BO, Bolivia; PE, Peru; EC, Ecuador; CO, Colombia; and VE, Venezuela.

| **Family name** | **Species name** | **VE** | **CO** | **EC** | **PE** | **BO** | **AR** |
| --- | --- | --- | --- | --- | --- | --- | --- |
| Acanthaceae | *Bravaisia integerrima* |  | 7 |  |  |  |  |
|  | *Trichanthera gigantea* |  | 38 |  |  |  |  |
| Acardiaceae | *Anacardium excelsum* |  | 2 |  |  |  |  |
| Achariaceae | *Lindackeria laurina* |  | 18 |  |  |  |  |
|  | *Mayna grandifolia* |  | 2 |  |  |  |  |
|  | *Mayna odorata* |  | 2 |  |  |  |  |
| Achatocarpaceae | *Achatocarpus praecox* |  |  |  |  | 155 | 133 |
| Actinidiaceae | *Saurauia adenodonta* |  |  | 2 |  |  |  |
|  | *Saurauia brachybotrys* |  |  | 18 |  |  |  |
|  | *Saurauia bullosa* |  |  | 12 |  |  |  |
|  | *Saurauia glabra* |  |  |  |  | 3 |  |
|  | *Saurauia harlingii* |  |  | 1 |  |  |  |
|  | *Saurauia herthae* |  |  | 10 |  |  |  |
|  | *Saurauia laevigata* |  | 21 |  |  |  |  |
|  | *Saurauia peruviana* |  |  | 2 |  |  |  |
|  | *Saurauia prainiana* |  |  | 23 |  |  |  |
|  | *Saurauia pseudostrigillosa* |  |  | 12 |  |  |  |
|  | *Saurauia spectabilis* |  |  |  | 1 |  |  |
|  | *Saurauia tomentosa* |  |  | 126 |  |  |  |
|  | *Saurauia ursina* |  | 1 |  |  |  |  |
| Adoxaceae | *Sambucus nigra* |  |  |  |  |  | 438 |
|  | *Viburnum ayavacense* |  |  |  | 18 |  |  |
|  | *Viburnum hallii* |  |  |  | 1 |  |  |
|  | *Viburnum pichinchense* |  |  | 32 |  |  |  |
|  | *Viburnum reticulatum* |  |  |  | 2 |  |  |
|  | *Viburnum seemenii* |  |  |  |  |  | 621 |
|  | *Viburnum stipitatum* |  |  | 3 |  |  |  |
|  | *Viburnum tinoides* |  | 2 |  |  |  |  |
|  | *Viburnum toronis* |  | 1 |  |  |  |  |
|  | *Viburnum triphyllum* |  |  | 6 | 54 |  |  |
|  | *Viburnum undulatum* |  | 13 |  |  |  |  |
| Alzateaceae | *Alzatea verticillata* |  |  | 27 | 363 |  |  |
| Amaryllidaceae | *Pancratium maximum* |  |  | 1 |  |  |  |
| Anacardiaceae | *Astronium graveolens* |  |  |  |  | 4 |  |
|  | *Astronium lecointei* |  | 35 |  |  | 1 |  |
|  | *Astronium urundeuva* |  |  |  |  |  | 299 |
|  | *Loxopterygium grisebachii* |  |  |  |  |  | 10 |
|  | *Mauria ferruginea* |  | 2 |  |  |  |  |
|  | *Mauria heterophylla* |  |  | 18 |  |  |  |
|  | *Myracrodruon urundeuva* |  |  |  |  | 118 |  |
|  | *Schinopsis brasiliensis* |  |  |  |  | 38 |  |
|  | *Schinopsis marginata* |  |  |  |  |  | 46 |
|  | *Schinus gracilipes* |  |  |  |  |  | 98 |
|  | *Schinus meyeri* |  |  |  |  |  | 12 |
|  | *Spondias mombin* |  | 14 | 1 |  |  |  |
|  | *Spondias radlkoferi* |  | 2 |  |  |  |  |
|  | *Spondias testudines* |  |  |  | 5 |  |  |
|  | *Tapirira guianensis* |  | 103 | 78 | 60 | 51 |  |
|  | *Tapirira obtusa* |  |  | 15 |  |  |  |
|  | *Toxicodendron striatum* |  | 5 |  |  |  |  |
| Annonaceae | *Anaxagorea brevipes* |  |  |  | 1 |  |  |
|  | *Anaxagorea dolichocarpa* |  |  | 1 |  |  |  |
|  | *Annona amazonica* |  | 2 |  |  |  |  |
|  | *Annona andicola* |  |  | 2 | 3 |  |  |
|  | *Annona boliviana* |  |  |  |  | 2 |  |
|  | *Annona cherimola* |  |  | 1 |  |  |  |
|  | *Annona dolichopetala* |  |  | 7 |  |  |  |
|  | *Annona edulis* |  |  | 3 |  |  |  |
|  | *Annona emarginata* |  |  |  |  | 36 | 4 |
|  | *Annona glabra* |  | 1 |  |  |  |  |
|  | *Annona hypoglauca* |  |  | 2 |  |  |  |
|  | *Annona mucosa* |  | 7 | 5 |  |  |  |
|  | *Annona neochrysocarpa* |  |  |  | 1 |  |  |
|  | *Annona neoulei* |  |  |  | 1 |  |  |
|  | *Annona papilionella* |  | 3 | 4 |  |  |  |
|  | *Annona spraguei* |  | 1 |  |  |  |  |
|  | *Cremastosperma macrophyllum* |  |  |  | 1 |  |  |
|  | *Cremastosperma megalophyllum* |  |  | 7 |  |  |  |
|  | *Cremastosperma napoense* |  |  | 2 |  |  |  |
|  | *Cremastosperma pedunculatum* |  |  | 1 |  |  |  |
|  | *Cymbopetalum longipes* |  |  |  | 1 |  |  |
|  | *Duguetia antioquensis* |  | 2 |  |  |  |  |
|  | *Duguetia macrophylla* |  |  | 1 |  |  |  |
|  | *Duguetia surinamensis* |  |  | 1 |  |  |  |
|  | *Ephedranthus colombianus* |  | 22 |  |  |  |  |
|  | *Guatteria aberrans* |  | 9 |  |  |  |  |
|  | *Guatteria alutacea* |  |  | 1 |  |  |  |
|  | *Guatteria asplundiana* |  |  | 5 |  |  |  |
|  | *Guatteria blepharophylla* |  |  | 3 |  |  |  |
|  | *Guatteria crassipes* |  |  | 1 |  |  |  |
|  | *Guatteria decurrens* |  |  | 12 |  |  |  |
|  | *Guatteria dolichopoda* |  | 2 |  |  |  |  |
|  | *Guatteria duodecima* |  |  | 2 |  |  |  |
|  | *Guatteria gentryi* |  |  | 1 |  |  |  |
|  | *Guatteria glauca* |  |  | 2 |  |  |  |
|  | *Guatteria goudotiana* |  | 87 |  |  |  |  |
|  | *Guatteria hirsuta* |  | 4 |  |  |  |  |
|  | *Guatteria longicuspis* |  |  | 1 |  |  |  |
|  | *Guatteria megalophylla* |  | 1 | 1 |  |  |  |
|  | *Guatteria modesta* |  |  | 5 |  |  |  |
|  | *Guatteria multivenia* |  |  | 1 |  |  |  |
|  | *Guatteria oblongifolia* |  |  | 1 | 4 | 22 |  |
|  | *Guatteria pastazae* |  |  | 1 |  |  |  |
|  | *Guatteria pittieri* |  | 1 |  |  |  |  |
|  | *Guatteria punctata* |  |  | 11 | 18 | 73 |  |
|  | *Guatteria terminalis* |  |  |  | 21 |  |  |
|  | *Guatteria tomentosa* |  |  |  |  | 2 |  |
|  | *Guatteria ucayalina* |  | 11 | 5 |  |  |  |
|  | *Klarobelia anomala* |  | 5 |  |  |  |  |
|  | *Klarobelia candida* |  |  |  | 4 |  |  |
|  | *Klarobelia napoensis* |  |  | 5 |  |  |  |
|  | *Malmea dimera* |  | 1 |  |  |  |  |
|  | *Mosannona hypoglauca* |  | 4 |  |  |  |  |
|  | *Oxandra espintana* |  |  |  |  | 640 |  |
|  | *Oxandra panamensis* |  | 17 |  |  |  |  |
|  | *Oxandra sphaerocarpa* |  |  |  | 1 |  |  |
|  | *Oxandra venezuelana* |  | 5 |  |  |  |  |
|  | *Porcelia mediocris* |  |  | 2 |  |  |  |
|  | *Porcelia nitida* |  |  |  | 2 |  |  |
|  | *Porcelia ponderosa* |  |  |  |  | 3 |  |
|  | *Pseudomalmea boyacana* |  | 2 |  |  |  |  |
|  | *Pseudoxandra sclerocarpa* |  | 6 |  |  |  |  |
|  | *Ruizodendron ovale* |  |  | 1 |  |  |  |
|  | *Trigynaea duckei* |  |  | 2 |  |  |  |
|  | *Unonopisis floribunda* |  |  |  | 6 |  |  |
|  | *Unonopsis aviceps* |  | 1 |  |  |  |  |
|  | *Unonopsis colombiana* |  | 1 |  |  |  |  |
|  | *Unonopsis floribunda* |  |  | 3 |  |  |  |
|  | *Unonopsis spectabilis* |  |  | 1 |  |  |  |
|  | *Xylopia amazonica* |  | 1 |  |  |  |  |
|  | *Xylopia calophylla* |  |  | 1 |  |  |  |
|  | *Xylopia cuspidata* |  |  | 9 |  |  |  |
|  | *Xylopia emarginata* |  | 3 |  |  |  |  |
|  | *Xylopia frutescens* |  | 13 |  |  |  |  |
|  | *Xylopia macrantha* |  | 2 |  |  |  |  |
|  | *Xylopia sericea* |  | 13 |  |  |  |  |
| Apocynaceae | *Aspidosperma cylindrocarpon* |  |  |  |  | 92 |  |
|  | *Aspidosperma darienense* |  |  | 2 |  |  |  |
|  | *Aspidosperma desmanthum* |  | 5 |  |  |  |  |
|  | *Aspidosperma excelsum* |  |  | 1 |  |  |  |
|  | *Aspidosperma macrocarpon* |  |  |  |  | 35 |  |
|  | *Aspidosperma megalocarpon* |  | 4 |  |  |  |  |
|  | *Aspidosperma quebracho-blanco* |  |  |  |  |  | 6 |
|  | *Aspidosperma rigidum* |  |  | 10 |  | 23 |  |
|  | *Aspidosperma spruceanum* |  | 1 | 1 |  |  |  |
|  | *Couma macrocarpa* |  | 22 |  |  |  |  |
|  | *Forsteronia affinis* |  | 1 |  |  |  |  |
|  | *Forsteronia australis* |  |  |  |  | 2 |  |
|  | *Forsteronia pubescens* |  |  |  |  | 1 |  |
|  | *Himatanthus articulatus* |  | 7 |  |  |  |  |
|  | *Himatanthus bracteatus* |  |  | 1 |  |  |  |
|  | *Lacmellea edulis* |  | 26 |  |  |  |  |
|  | *Lacmellea panamensis* |  | 1 |  |  |  |  |
|  | *Lacmellea speciosa* |  |  | 1 |  |  |  |
|  | *Malouetia guatemalensis* |  | 7 |  |  |  |  |
|  | *Odontadenia verrucosa* |  | 1 |  |  |  |  |
|  | *Rauvolfia leptophylla* |  |  | 1 |  | 4 |  |
|  | *Rauvolfia littoralis* |  | 1 |  |  |  |  |
|  | *Rauvolfia praecox* |  |  | 1 |  |  |  |
|  | *Tabernaemontana cymosa* |  |  |  |  | 9 |  |
|  | *Tabernaemontana grandiflora* |  | 1 |  |  |  |  |
|  | *Tabernaemontana heterophylla* |  |  | 1 |  |  |  |
|  | *Tabernaemontana longipes* |  | 1 |  |  |  |  |
|  | *Tabernaemontana marckgarviana* |  | 1 |  |  |  |  |
| Aquifoliaceae | *Ilex aggregata* |  |  |  | 3 | 1 |  |
|  | *Ilex amboroica* |  |  | 1 |  |  |  |
|  | *Ilex amplifolia* |  |  |  |  | 3 |  |
|  | *Ilex andicola* |  |  | 39 |  |  |  |
|  | *Ilex argentina* |  |  |  |  |  | 472 |
|  | *Ilex biserrulata* |  |  |  | 25 |  |  |
|  | *Ilex colombiana* |  |  | 55 |  |  |  |
|  | *Ilex crassifolioides* |  |  |  | 64 |  |  |
|  | *Ilex danielis* |  | 5 |  |  |  |  |
|  | *Ilex gabinetensis* |  |  | 1 |  |  |  |
|  | *Ilex goudotii* |  |  |  |  | 3 |  |
|  | *Ilex guayusa* |  |  |  | 3 |  |  |
|  | *Ilex hippocrateoides* |  |  | 1 |  | 48 |  |
|  | *Ilex karstenii* |  |  |  | 1 |  |  |
|  | *Ilex laurina* |  | 18 | 22 | 17 |  |  |
|  | *Ilex macarenensis* |  |  | 2 |  |  |  |
|  | *Ilex maxima* |  |  | 9 |  |  |  |
|  | *Ilex microdonta* |  |  |  | 12 |  |  |
|  | *Ilex microsticta* |  |  |  |  | 1 |  |
|  | *Ilex myricoides* |  |  | 10 |  |  |  |
|  | *Ilex nervosa* |  | 8 | 2 | 2 |  |  |
|  | *Ilex pernervata* |  | 5 |  |  |  |  |
|  | *Ilex petiolaris* |  |  |  |  | 8 |  |
|  | *Ilex pustulosa* |  | 5 |  |  |  |  |
|  | *Ilex rimbachii* |  |  | 3 |  |  |  |
|  | *Ilex rupicola* |  |  | 15 |  |  |  |
|  | *Ilex sessiliflora* |  |  |  | 70 |  |  |
|  | *Ilex teratopis* |  |  | 3 |  |  |  |
|  | *Ilex trichoclada* |  |  |  | 2 |  |  |
|  | *Ilex villosula* |  |  |  | 99 |  |  |
|  | *Ilex weberlingii* |  |  | 9 |  |  |  |
|  | *Ilex yurumanguinis* |  |  | 4 |  |  |  |
| Araliaceae | *Aralia soratensis* |  |  |  |  | 3 | 96 |
|  | *Dendropanax arboreus* |  | 29 | 9 |  | 3 |  |
|  | *Dendropanax caucanus* |  |  | 7 |  |  |  |
|  | *Dendropanax inaequalipedunculatus* | |  |  |  | 43 |  |
|  | *Dendropanax macrophyllus* |  | 8 | 9 |  |  |  |
|  | *Oreopanax andreanus* |  |  | 13 | 12 |  |  |
|  | *Oreopanax avicenniifolius* |  |  | 21 |  |  |  |
|  | *Oreopanax boliviensis* |  |  |  |  | 6 |  |
|  | *Oreopanax capitatus* |  |  |  | 11 |  |  |
|  | *Oreopanax ecuadorensis* |  |  | 172 |  |  |  |
|  | *Oreopanax eriocephalus* |  |  | 2 |  |  |  |
|  | *Oreopanax grandifolius* |  |  | 1 |  |  |  |
|  | *Oreopanax incisus* |  | 2 | 4 |  |  |  |
|  | *Oreopanax kuntzei* |  |  |  | 1 |  | 50 |
|  | *Oreopanax microflorous* |  |  |  | 19 |  |  |
|  | *Oreopanax obscurus* |  |  | 2 |  |  |  |
|  | *Oreopanax oroyanus* |  |  |  | 3 |  |  |
|  | *Oreopanax palamophyllus* |  |  | 19 |  |  |  |
|  | *Oreopanax rosei* |  |  |  | 22 |  |  |
|  | *Oreopanax ruizii* |  |  |  |  | 18 |  |
|  | *Oreopanax seemanianus* |  |  | 3 |  |  |  |
|  | *Oreopanax sessiliflorus* |  |  | 6 |  |  |  |
|  | *Oreopanax steinbachianus* |  |  |  |  | 10 |  |
|  | *Oreopanax trifolius* |  |  |  | 2 |  |  |
|  | *Oreopanax trollii* |  |  |  |  | 7 |  |
|  | *Schefflera acuminata* |  |  | 2 |  |  |  |
|  | *Schefflera allocotantha* |  |  |  | 37 |  |  |
|  | *Schefflera coriacea* |  |  | 4 |  |  |  |
|  | *Schefflera dielsii* |  |  | 6 |  |  |  |
|  | *Schefflera ferruginea* |  |  | 3 |  |  |  |
|  | *Schefflera herzogii* |  |  |  |  | 54 |  |
|  | *Schefflera humboldtiana* |  |  | 7 |  |  |  |
|  | *Schefflera inambarica* |  |  |  | 1 |  |  |
|  | *Schefflera jahnii* |  | 2 |  |  |  |  |
|  | *Schefflera lasiogyne* |  |  | 3 |  |  |  |
|  | *Schefflera mathewsii* |  |  | 3 |  |  |  |
|  | *Schefflera morototoni* |  | 42 | 14 | 1 |  |  |
|  | *Schefflera patula* |  |  |  | 35 |  |  |
|  | *Schefflera pentandra* |  |  | 4 |  |  |  |
|  | *Schefflera sodiroi* |  |  | 12 |  |  |  |
|  | *Schefflera sprucei* |  |  |  | 2 |  |  |
|  | *Schefflera trianae* |  | 1 |  |  |  |  |
| Arecaceae | *Aiphanes horrida* |  | 4 |  |  |  |  |
|  | *Aphandra natalia* |  |  | 3 |  |  |  |
|  | *Astrocaryum chambira* |  |  | 9 |  |  |  |
|  | *Astrocaryum murumuru* |  |  |  | 2 | 1 |  |
|  | *Astrocaryum standleyanum* |  | 75 |  |  |  |  |
|  | *Attalea butyracea* |  | 1 |  |  |  |  |
|  | *Bactris gasipaes* |  |  | 7 |  | 1 |  |
|  | *Bactris pilosa* |  | 5 |  |  |  |  |
|  | *Bactris setulosa* |  |  | 1 |  |  |  |
|  | *Ceroxylon echinulatum* |  |  | 19 |  |  |  |
|  | *Ceroxylon parvifrons* |  |  | 4 |  | 1 |  |
|  | *Dictyocaryum lamarckianum* |  |  | 29 | 4 | 42 |  |
|  | *Euterpe precatoria* |  | 11 | 1 | 1 | 5 |  |
|  | *Geonoma cuneata* |  |  | 11 |  |  |  |
|  | *Geonoma orbignyana* |  |  | 12 |  |  |  |
|  | *Geonoma stricta* |  |  | 1 |  |  |  |
|  | *Geonoma undata* |  | 2 | 3 |  |  |  |
|  | *Iriartea deltoidea* |  |  | 342 | 5 | 10 |  |
|  | *Oenocarpus bataua* |  | 60 | 27 |  | 1 |  |
|  | *Oenocarpus minor* |  | 181 |  |  |  |  |
|  | *Phytelephas aequatorialis* |  |  | 76 |  |  |  |
|  | *Prestoea acuminata* |  | 1 | 13 |  |  |  |
|  | *Prestoea decurrens* |  |  | 3 |  |  |  |
|  | *Prestoea ensiformis* |  |  | 1 |  |  |  |
|  | *Socratea exorrhiza* |  | 46 | 9 | 1 | 283 |  |
|  | *Socratea rostrata* |  |  | 2 |  |  |  |
|  | *Socratea salazarii* |  |  |  | 3 |  |  |
|  | *Syagrus sancona* |  |  |  |  | 3 |  |
|  | *Wettinia aequalis* |  |  | 28 |  |  |  |
|  | *Wettinia anomala* |  |  | 22 |  |  |  |
|  | *Wettinia fascicularis* |  | 21 | 1 |  |  |  |
|  | *Wettinia hirsuta* |  | 17 |  |  |  |  |
|  | *Wettinia kalbreyeri* |  | 316 | 24 |  |  |  |
|  | *Wettinia maynensis* |  |  | 110 |  |  |  |
|  | *Wettinia quinaria* |  |  | 53 |  |  |  |
| Asparagaceae | *Cordyline congesta* |  |  |  |  |  | 1 |
| Asteraceae | *Ageratina gloeoclada* |  |  |  |  | 2 |  |
|  | *Baccharis buchtienii* |  |  |  |  | 6 |  |
|  | *Baccharis latifolia* |  |  |  |  |  | 19 |
|  | *Baccharis oblongifolia* |  |  | 1 | 1 |  |  |
|  | *Baccharis padifolia* |  |  | 4 |  |  |  |
|  | *Barnadesia parviflora* |  |  | 4 |  |  |  |
|  | *Cnicothamnus lorentzii* |  |  |  |  |  | 2 |
|  | *Critonia arachnoidea* |  |  |  |  |  | 25 |
|  | *Critoniopsis boliviana* |  |  | 4 |  |  |  |
|  | *Critoniopsis choquetangensis* |  |  |  |  | 8 |  |
|  | *Critoniopsis floribunda* |  |  | 6 | 74 |  |  |
|  | *Critoniopsis occidentalis* |  |  | 66 |  |  |  |
|  | *Critoniopsis pycnantha* |  |  | 12 |  |  |  |
|  | *Critoniopsis sodiroi* |  |  | 10 |  |  |  |
|  | *Critoniopsis zamorensis* |  |  | 7 |  |  |  |
|  | *Dasyphyllum brasiliense* |  |  |  |  |  | 5 |
|  | *Dendrophorbium lloense* |  |  | 14 |  |  |  |
|  | *Dendrophorbium tipocochensis* |  |  | 4 |  |  |  |
|  | *Diplostephium floribundum* |  |  | 2 |  |  |  |
|  | *Ferreyranthus verbascifolius* |  |  | 11 |  |  |  |
|  | *Grosvenoria rimbachii* |  |  | 6 |  |  |  |
|  | *Gynoxis induta* |  |  | 1 |  |  |  |
|  | *Gynoxys acostae* |  |  | 24 |  |  |  |
|  | *Gynoxys azuayensis* |  |  | 46 |  |  |  |
|  | *Gynoxys hallii* |  |  | 10 |  |  |  |
|  | *Gynoxys laurifolia* |  |  | 9 |  |  |  |
|  | *Gynoxys mandonii* |  |  |  |  | 25 |  |
|  | *Gynoxys pulchella* |  |  | 1 |  |  |  |
|  | *Gynoxys rimbachii* |  |  | 1 |  |  |  |
|  | *Gynoxys validifolia* |  |  | 1 |  |  |  |
|  | *Kaunia lasiophthalma* |  |  |  |  |  | 28 |
|  | *Kaunia saltensis* |  |  |  |  |  | 27 |
|  | *Liabum solidagineum* |  |  |  |  | 1 |  |
|  | *Nordenstamia repanda* |  |  |  | 8 | 9 |  |
|  | *Pappobolus acuminatus* |  |  | 1 |  |  |  |
|  | *Pentacalia oronocensis* |  |  |  | 2 | 1 |  |
|  | *Piptocarpha poeppigiana* |  |  |  |  | 1 |  |
|  | *Piptocoma discolor* |  | 10 | 46 |  |  |  |
|  | *Tessaria integrifolia* |  |  |  |  |  | 2 |
|  | *Verbesina arborea* |  |  | 19 |  |  |  |
|  | *Verbesina brachypoda* |  |  | 6 |  |  |  |
|  | *Verbesina latisquama* |  |  | 33 |  |  |  |
|  | *Verbesina lloensis* |  |  | 7 |  |  |  |
|  | *Verbesina nudipes* |  | 1 | 11 |  |  |  |
|  | *Vernonanthura patens* |  |  | 1 |  |  |  |
| Berberidaceae | *Berberis jobii* |  |  |  |  |  | 26 |
|  | *Berberis rigida* |  |  | 3 |  |  |  |
| Betulaceae | *Alnus acuminata* |  |  | 47 | 23 |  | 495 |
| Bignoniaceae | *Amphitecna latifolia* |  | 1 |  |  |  |  |
|  | *Delostoma integrifolium* |  |  |  | 2 |  |  |
|  | *Fridericia florida* |  | 1 |  |  |  |  |
|  | *Fridericia pearcei* |  |  |  |  | 4 |  |
|  | *Handroanthus chrysanthus* |  |  | 45 |  | 57 |  |
|  | *Handroanthus impetiginosus* |  |  |  |  | 19 | 196 |
|  | *Handroanthus lapacho* |  |  |  |  |  | 246 |
|  | *Handroanthus ochraceus* |  | 1 |  |  |  | 68 |
|  | *Handroanthus serratifolius* |  |  | 10 |  | 6 |  |
|  | *Jacaranda caucana* |  | 25 |  |  |  |  |
|  | *Jacaranda copaia* |  | 103 | 13 |  |  |  |
|  | *Jacaranda hesperia* |  | 1 |  |  |  |  |
|  | *Jacaranda mimosifolia* |  |  |  |  |  | 49 |
|  | *Parmentiera stenocarpa* |  | 4 |  |  |  |  |
|  | *Tabebuia rosea* |  | 3 |  |  |  |  |
|  | *Tecoma stans* |  |  |  |  |  | 815 |
|  | *Tynanthus schumannianus* |  |  |  |  | 2 |  |
|  | *Xylophragma pratense* |  |  |  |  | 3 |  |
| Bixaceae | *Cochlospermum vitifolium* |  |  |  |  | 6 |  |
| Blechnaceae | *Blechnum loxense* |  |  | 19 |  |  |  |
| Boraginaceae | *Cordia hebeclada* |  |  | 1 |  |  |  |
|  | *Cordia mexiana* |  |  | 1 |  |  |  |
| Brunelliaceae | *Brunellia acostae* |  |  | 2 |  |  |  |
|  | *Brunellia boliviana* |  |  |  | 3 | 36 |  |
|  | *Brunellia brunnea* |  |  |  | 1 |  |  |
|  | *Brunellia comocladifolia* |  |  | 3 |  |  |  |
|  | *Brunellia cuzcoensis* |  |  |  | 1 |  |  |
|  | *Brunellia dulcis* |  |  |  | 5 |  |  |
|  | *Brunellia goudotii* |  | 5 |  |  |  |  |
|  | *Brunellia inermis* |  |  | 8 | 41 |  |  |
|  | *Brunellia littlei* |  |  |  | 4 |  |  |
|  | *Brunellia pallida* |  |  | 8 |  |  |  |
|  | *Brunellia pauciflora* |  |  | 6 |  |  |  |
|  | *Brunellia rhoides* |  |  |  |  | 7 |  |
|  | *Brunellia sibundoya* |  | 26 |  |  |  |  |
|  | *Brunellia tomentosa* |  |  | 65 |  |  |  |
|  | *Brunellia trianae* |  | 13 |  |  |  |  |
|  | *Brunellia weberbaueri* |  |  |  | 4 |  |  |
| Burseraceae | *Bursera inversa* |  | 1 |  |  |  |  |
|  | *Bursera simaruba* |  | 10 |  |  |  |  |
|  | *Crepidospermum rhoifolium* |  | 17 |  |  |  |  |
|  | *Dacryodes affcupularis* |  |  | 11 |  |  |  |
|  | *Dacryodes belemensis* |  | 27 |  |  |  |  |
|  | *Dacryodes cupularis* |  |  | 44 |  |  |  |
|  | *Dacryodes macrophylla* |  |  | 7 |  |  |  |
|  | *Dacryodes peruviana* |  | 1 | 89 |  |  |  |
|  | *Protium amazonicum* |  |  | 14 |  |  |  |
|  | *Protium amplum* |  |  | 1 |  |  |  |
|  | *Protium apiculatum* |  | 1 |  |  |  |  |
|  | *Protium aracouchini* |  | 1 | 1 |  |  |  |
|  | *Protium colombianum* |  | 7 |  |  |  |  |
|  | *Protium ecuadorense* |  |  | 13 |  |  |  |
|  | *Protium glabrescens* |  |  | 1 |  |  |  |
|  | *Protium guianense* |  | 1 |  |  |  |  |
|  | *Protium meridionale* |  |  |  |  | 13 |  |
|  | *Protium montanum* |  |  |  | 15 |  |  |
|  | *Protium nodulosum* |  |  | 42 |  |  |  |
|  | *Protium panamense* |  | 7 |  |  |  |  |
|  | *Protium sagotianum* |  | 17 | 7 |  |  |  |
|  | *Protium tovarense* |  | 29 |  |  |  |  |
|  | *Tetragastris altissima* |  |  |  | 4 |  |  |
|  | *Tetragastris panamensis* |  | 56 | 17 |  |  |  |
|  | *Trattinnickia aspera* |  | 2 |  |  |  |  |
|  | *Trattinnickia lawrancei* |  | 7 | 9 |  |  |  |
|  | *Trattinnickia rhoifolia* |  |  | 1 |  |  |  |
| Buxaceae | *Styloceras laurifolium* |  |  |  | 50 |  |  |
| Cactaceae | *Brasiliopuntia brasiliensis* |  |  |  |  | 49 |  |
|  | *Cereus forbesii* |  |  |  |  |  | 4 |
|  | *Cereus stenogonus* |  |  |  |  | 62 |  |
|  | *Cereus yungasensis* |  |  |  |  | 2 |  |
| Calophyllaceae | *Calophyllum brasiliense* |  | 25 | 5 |  | 1 |  |
|  | *Kielmeyera paniculata* |  |  |  |  | 16 |  |
|  | *Marila laxiflora* |  | 2 |  |  |  |  |
|  | *Marila magnifica* |  |  | 2 |  |  |  |
|  | *Marila pluricostata* |  |  | 1 |  |  |  |
|  | *Marila podantha* |  | 4 |  |  |  |  |
|  | *Marila tomentosa* |  |  | 5 |  |  |  |
| Campanulaceae | *Cyanea pinnatifida* |  |  | 1 |  |  |  |
| Cannabaceae | *Celtis ehrenbergiana* |  |  |  |  |  | 3 |
|  | *Celtis loxensis* |  |  |  |  | 31 |  |
|  | *Celtis schippii* |  | 2 | 8 | 4 | 16 |  |
|  | *Lozanella enantiophylla* |  |  | 1 | 12 |  |  |
|  | *Trema integerrima* |  |  | 1 |  |  |  |
|  | *Trema micrantha* |  | 18 | 7 |  | 1 |  |
| Capparaceae | *Capparidastrum coimbranum* |  |  |  |  | 134 |  |
|  | *Capparidastrum petiolare* |  |  |  |  |  | 76 |
|  | *Capparidastrum tafallanum* |  |  | 2 |  |  |  |
|  | *Cynophalla amplissima* |  |  |  |  | 8 |  |
|  | *Cynophalla polyantha* |  |  |  |  | 239 |  |
|  | *Cynophalla retusa* |  |  |  |  |  | 1 |
|  | *Preslianthus detonsus* |  | 1 | 4 |  |  |  |
| Cardiopteridaceae | *Citronella apogon* |  |  |  |  | 2 | 51 |
|  | *Citronella ilicifolia* |  |  | 1 | 4 |  |  |
|  | *Citronella incarum* |  |  | 5 |  |  |  |
|  | *Citronella meliodora* |  |  |  | 1 |  |  |
|  | *Dendrobangia boliviana* |  | 15 | 6 |  |  |  |
| Caricaceae | *Carica papaya* |  |  |  |  | 1 |  |
|  | *Jacaratia digitata* |  |  | 7 |  | 1 |  |
|  | *Jacaratia spinosa* |  |  | 5 |  |  |  |
|  | *Vasconcellea palandensis* |  |  | 1 |  |  |  |
|  | *Vasconcellea quercifolia* |  |  |  |  |  | 12 |
| Caryocaraceae | *Caryocar amygdaliferum* |  | 3 |  |  |  |  |
|  | *Caryocar dentatum* |  |  | 1 |  | 3 |  |
|  | *Caryocar glabrum* |  | 25 | 2 | 2 |  |  |
| Celastraceae | *Celastrus liebmanii* | 3 |  |  |  |  |  |
|  | *Cheiloclinium cognatum* |  |  |  |  | 33 |  |
|  | *Cheiloclinium peruvianum* |  |  |  | 1 |  |  |
|  | *Haydenoxylon gentryi* |  |  | 2 |  |  |  |
|  | *Haydenoxylon urbanianum* |  |  |  |  | 21 |  |
|  | *Hippocratea volubilis* |  |  |  |  | 1 |  |
|  | *Maytenus macrocarpa* |  |  | 1 |  |  |  |
|  | *Monteverdia apurimacensis* |  |  | 3 |  |  |  |
|  | *Monteverdia ebenifolia* |  |  | 3 |  |  |  |
|  | *Monteverdia floribunda* |  |  |  |  | 56 |  |
|  | *Monteverdia krukovii* |  |  | 3 |  |  |  |
|  | *Monteverdia macrocarpa* |  | 5 | 2 |  |  |  |
|  | *Peritassa peruviana* |  |  |  |  | 4 |  |
|  | *Prionostemma asperum* |  | 5 |  |  |  |  |
|  | *Pristimera celastroides* |  |  |  |  | 3 |  |
|  | *Salacia cordata* |  |  | 11 |  |  |  |
|  | *Schaefferia argentinensis* |  |  |  |  | 1 |  |
|  | *Tontelea emarginata* |  |  | 1 |  |  |  |
|  | *Zinowiewia australis* |  | 2 |  |  |  |  |
|  | *Zinowiewia madsenii* |  |  | 7 |  |  |  |
| Chloranthaceae | *Hedyosmum angustifolium* |  |  |  |  | 12 |  |
|  | *Hedyosmum anisodorum* |  |  | 43 | 8 |  |  |
|  | *Hedyosmum bonplandianum* |  | 5 |  |  |  |  |
|  | *Hedyosmum cuatrecazanum* |  | 40 | 35 | 13 | 16 |  |
|  | *Hedyosmum cumbalense* |  |  | 121 |  |  |  |
|  | *Hedyosmum dombeyanum* |  |  |  |  | 15 |  |
|  | *Hedyosmum gentryi* |  | 1 |  |  |  |  |
|  | *Hedyosmum goudotianum* |  | 1 | 150 | 64 |  |  |
|  | *Hedyosmum grande* |  |  | 1 |  |  |  |
|  | *Hedyosmum lechleri* |  |  |  | 1 | 109 |  |
|  | *Hedyosmum luteynii* |  |  | 125 |  |  |  |
|  | *Hedyosmum maximum* |  |  |  | 10 |  |  |
|  | *Hedyosmum peruvianum* |  |  |  | 5 |  |  |
|  | *Hedyosmum purpurascens* |  |  | 24 |  |  |  |
|  | *Hedyosmum racemosum* | 166 | 6 | 8 | 208 | 142 |  |
|  | *Hedyosmum scaberrimum* |  |  | 24 |  |  |  |
|  | *Hedyosmum scabrum* |  |  | 24 | 535 | 84 |  |
|  | *Hedyosmum sprucei* |  |  | 12 |  |  |  |
|  | *Hedyosmum strigosum* |  |  | 2 |  |  |  |
|  | *Hedyosmum translucidum* |  |  | 6 | 5 |  |  |
| Chrysobalanaceae | *Couepia platycalyx* |  | 9 | 2 |  |  |  |
|  | *Hirtella aequatoriensis* |  |  | 5 |  |  |  |
|  | *Hirtella americana* |  |  | 2 |  |  |  |
|  | *Hirtella bicornis* |  | 1 |  |  |  |  |
|  | *Hirtella lightioides* |  |  |  |  | 120 |  |
|  | *Hirtella mutisii* |  | 1 | 4 |  |  |  |
|  | *Hirtella racemosa* |  |  | 2 | 1 |  |  |
|  | *Hirtella recurva* |  |  | 1 |  |  |  |
|  | *Hirtella schultesii* |  |  | 1 |  |  |  |
|  | *Hirtella triandra* |  |  | 2 |  | 10 |  |
|  | *Hymenopus krukovii* |  |  |  |  | 1 |  |
|  | *Hymenopus parviflora* |  | 3 |  |  |  |  |
|  | *Licania apetala* |  | 2 | 2 |  |  |  |
|  | *Licania canescens* |  |  |  | 1 |  |  |
|  | *Licania celiae* |  |  | 2 |  |  |  |
|  | *Licania harlingii* |  |  | 1 |  |  |  |
|  | *Licania hypoleuca* |  | 4 |  |  |  |  |
|  | *Licania macrocarpa* |  |  | 2 |  |  |  |
|  | *Licania micrantha* |  |  | 4 |  |  |  |
|  | *Moquilea boliviensis* |  |  |  |  | 10 |  |
|  | *Moquilea brittoniana* |  |  | 2 |  |  |  |
|  | *Moquilea durifolia* |  |  | 13 |  |  |  |
|  | *Parinari klugii* |  |  | 1 |  |  |  |
|  | *Parinari occidentalis* |  |  |  |  | 4 |  |
| Clethraceae | *Clethra cardenasii* |  |  |  |  | 2 |  |
|  | *Clethra crispa* |  |  | 21 |  |  |  |
|  | *Clethra cuneata* |  |  |  | 65 | 212 |  |
|  | *Clethra elongata* |  |  |  |  | 36 |  |
|  | *Clethra fagifolia* | 29 | 103 | 10 |  |  |  |
|  | *Clethra ferruginea* |  |  |  | 36 | 110 |  |
|  | *Clethra fimbriata* |  |  | 20 | 2 |  |  |
|  | *Clethra lanata* |  | 1 |  |  |  |  |
|  | *Clethra obovata* |  |  |  | 4 |  |  |
|  | *Clethra ovalifolia* |  |  | 23 |  |  |  |
|  | *Clethra parallelinervia* |  |  | 1 |  |  |  |
|  | *Clethra pedicellaris* |  |  | 1 |  | 7 |  |
|  | *Clethra peruviana* |  |  |  |  | 72 |  |
|  | *Clethra revoluta* |  | 12 | 32 | 197 | 344 |  |
|  | *Clethra scabra* |  |  |  |  | 5 | 11 |
|  | *Purdiaea nutans* |  |  | 29 |  |  |  |
| Clusiaceae | *Chrysochlamys bracteolata* |  |  | 22 |  |  |  |
|  | *Chrysochlamys colombiana* |  | 32 | 27 |  |  |  |
|  | *Chrysochlamys dependens* |  | 3 | 27 |  |  |  |
|  | *Chrysochlamys eclipes* |  | 8 |  |  |  |  |
|  | *Chrysochlamys macrophylla* |  | 1 | 3 | 1 |  |  |
|  | *Chrysochlamys membranacea* |  |  | 29 |  |  |  |
|  | *Chrysochlamys nicaraguensis* |  |  | 4 |  |  |  |
|  | *Chrysochlamys weberbaueri* |  | 6 | 4 |  |  |  |
|  | *Clusia alata* |  | 25 | 86 | 424 |  |  |
|  | *Clusia cuneifolia* |  | 5 |  |  |  |  |
|  | *Clusia dixonii* |  | 1 | 2 |  |  |  |
|  | *Clusia ducu* |  | 6 | 12 |  | 53 |  |
|  | *Clusia ducuoides* |  | 18 | 30 | 29 |  |  |
|  | *Clusia elliptica* |  |  | 38 | 4 |  |  |
|  | *Clusia flavida* |  |  | 1 |  |  |  |
|  | *Clusia flaviflora* |  |  | 195 |  |  |  |
|  | *Clusia haughtii* |  |  |  |  | 14 |  |
|  | *Clusia hydrogera* |  | 7 |  |  |  |  |
|  | *Clusia inesiana* |  | 16 |  |  |  |  |
|  | *Clusia latipes* |  |  | 29 |  |  |  |
|  | *Clusia lechleri* |  |  |  |  | 114 |  |
|  | *Clusia lineata* |  | 1 |  |  |  |  |
|  | *Clusia loranthacea* |  |  | 1 |  |  |  |
|  | *Clusia magnifolia* |  |  | 1 |  |  |  |
|  | *Clusia multiflora* | 21 | 11 | 57 |  | 535 |  |
|  | *Clusia pachamamae* |  |  |  |  | 5 |  |
|  | *Clusia pavonii* |  |  |  | 2 |  |  |
|  | *Clusia sphaerocarpa* |  |  |  | 237 | 129 |  |
|  | *Clusia ternstroemioides* |  |  |  |  | 16 |  |
|  | *Clusia thurifera* |  |  |  | 141 |  |  |
|  | *Clusia trochiformis* |  | 1 | 1 | 84 | 10 |  |
|  | *Clusia venusta* |  |  | 1 |  |  |  |
|  | *Garcinia brasiliensis* |  |  | 1 |  |  |  |
|  | *Garcinia gardneriana* |  |  |  |  | 46 |  |
|  | *Garcinia intermedia* |  | 2 |  |  |  |  |
|  | *Garcinia macrophylla* |  |  | 28 |  | 119 |  |
|  | *Garcinia madruno* |  | 3 | 16 |  | 6 |  |
|  | *Garcinia magnifolia* |  | 16 |  |  |  |  |
|  | *Symphonia globulifera* |  |  | 2 | 2 | 3 |  |
|  | *Tovomita nicaraguensis* |  |  | 1 |  |  |  |
|  | *Tovomitopsis membranacea* |  |  | 8 |  |  |  |
| Combretaceae | *Terminalia amazonia* |  | 3 | 14 |  |  |  |
|  | *Terminalia oblonga* |  |  | 1 | 14 | 17 |  |
|  | *Terminalia pallidovirens* |  | 1 |  |  |  |  |
|  | *Terminalia pulcherrima* |  |  |  | 5 |  |  |
|  | *Terminalia tetraphylla* |  | 2 |  |  | 1 |  |
|  | *Terminalia triflora* |  |  |  |  | 69 | 631 |
| Cordiaceae | *Cordia alliodora* |  |  | 29 |  | 7 |  |
|  | *Cordia americana* |  |  |  |  |  | 419 |
|  | *Cordia bicolor* |  | 64 |  |  |  |  |
|  | *Cordia bogotensis* |  | 1 |  |  |  |  |
|  | *Cordia chamissoniana* |  |  | 1 |  |  |  |
|  | *Cordia collococca* |  |  | 2 |  |  |  |
|  | *Cordia colombiana* |  |  | 3 |  |  |  |
|  | *Cordia cymosa* |  |  | 1 |  |  |  |
|  | *Cordia eriostigma* |  |  | 1 |  |  |  |
|  | *Cordia lasiocalyx* |  | 1 |  |  |  |  |
|  | *Cordia nodosa* |  |  | 1 |  |  |  |
|  | *Cordia panamensis* |  | 40 | 6 |  |  |  |
|  | *Cordia protracta* |  | 2 |  |  |  |  |
|  | *Cordia saccellia* |  |  |  |  |  | 175 |
|  | *Cordia trichotoma* |  |  |  |  |  | 301 |
|  | *Cordia ucayaliensis* |  |  |  |  | 2 |  |
|  | *Varronia cylindrostachya* |  |  | 3 |  |  |  |
|  | *Varronia exarata* |  |  | 1 |  |  |  |
|  | *Varronia vasqueziana* |  |  | 1 |  |  |  |
| Cornaceae | *Cornus disciflora* |  |  | 2 |  |  |  |
|  | *Cornus peruviana* |  |  | 9 | 40 |  |  |
| Cunoniaceae | *Weinmannia auriculata* |  |  |  | 108 | 1 |  |
|  | *Weinmannia auriculifera* |  |  |  |  | 1 |  |
|  | *Weinmannia balbisiana* |  | 153 | 41 | 1 | 9 |  |
|  | *Weinmannia bangii* |  |  |  | 315 |  |  |
|  | *Weinmannia boliviensis* |  |  |  |  |  | 25 |
|  | *Weinmannia cochensis* |  |  | 6 | 179 |  |  |
|  | *Weinmannia crassifolia* |  |  |  | 384 | 288 |  |
|  | *Weinmannia davidsonii* |  |  |  |  | 87 |  |
|  | *Weinmannia elliptica* |  |  | 36 | 140 |  |  |
|  | *Weinmannia fagaroides* |  |  | 359 |  | 413 |  |
|  | *Weinmannia glabra* |  |  | 23 |  |  |  |
|  | *Weinmannia haenkeana* |  |  | 2 |  | 72 |  |
|  | *Weinmannia latifolia* |  |  | 1 |  |  |  |
|  | *Weinmannia lechleriana* | 24 |  |  | 10 | 49 |  |
|  | *Weinmannia lentiscifolia* |  |  | 7 |  | 1 |  |
|  | *Weinmannia loxensis* |  |  | 10 |  |  |  |
|  | *Weinmannia macrophylla* |  |  | 6 |  |  |  |
|  | *Weinmannia magnifolia* |  |  | 2 |  |  |  |
|  | *Weinmannia mariquitae* |  |  | 56 | 15 |  |  |
|  | *Weinmannia microphylla* |  |  | 6 |  |  |  |
|  | *Weinmannia multijuga* |  | 14 |  | 45 | 16 |  |
|  | *Weinmannia nebularum* |  |  |  |  | 3 |  |
|  | *Weinmannia ovata* |  |  | 61 | 50 | 213 |  |
|  | *Weinmannia parvifolia* |  |  | 1 | 1 |  |  |
|  | *Weinmannia pinnata* | 2 |  | 234 | 1 | 133 |  |
|  | *Weinmannia pubescens* |  | 4 | 41 | 105 |  |  |
|  | *Weinmannia reticulata* |  |  | 22 | 226 | 196 |  |
|  | *Weinmannia rollottii* |  |  | 84 |  |  |  |
|  | *Weinmannia sorbifolia* |  |  | 9 |  | 169 |  |
|  | *Weinmannia spruceana* |  |  | 4 |  |  |  |
| Cyatheaceae | *Alsophila cuspidata* |  |  | 36 | 1 |  |  |
|  | *Alsophila erinacea* |  | 7 | 4 | 2 | 1 |  |
|  | *Alsophila imrayana* |  |  | 36 |  |  |  |
|  | *Alsophila incana* |  | 1 |  |  |  |  |
|  | *Alsophila odonelliana* |  |  |  |  |  | 40 |
|  | *Alsophila engelii* | 16 |  |  |  |  |  |
|  | *Cyathea aterrima* |  |  | 9 |  |  |  |
|  | *Cyathea austropallescens* |  |  |  |  | 20 |  |
|  | *Cyathea caracasana* |  | 38 | 362 | 171 |  |  |
|  | *Cyathea carolihenrici* |  |  |  | 90 |  |  |
|  | *Cyathea catacampta* |  |  |  | 1 |  |  |
|  | *Cyathea corallifera* |  | 1 |  |  |  |  |
|  | *Cyathea cystolepis* |  |  |  | 1 |  |  |
|  | *Cyathea delgadii* |  | 1 |  | 697 | 2 |  |
|  | *Cyathea dintelmanii* |  |  |  |  | 1 |  |
|  | *Cyathea divergens* |  | 2 |  | 35 |  |  |
|  | *Cyathea frigida* |  |  | 24 |  |  |  |
|  | *Cyathea fulva* |  | 18 |  |  |  |  |
|  | *Cyathea guentheriana* |  |  | 3 |  |  |  |
|  | *Cyathea herzogii* |  |  |  | 6 | 455 |  |
|  | *Cyathea kalbreyeri* |  |  | 4 |  |  |  |
|  | *Cyathea lechleri* |  |  |  | 254 |  |  |
|  | *Cyathea moranii* |  |  | 43 |  |  |  |
|  | *Cyathea multisegmenta* |  |  |  | 9 |  |  |
|  | *Cyathea nigripes* |  |  | 6 |  |  |  |
|  | *Cyathea pallescens* |  |  | 2 | 2 |  |  |
|  | *Cyathea patens* |  |  | 4 |  |  |  |
|  | *Cyathea poeppigii* |  |  | 130 |  |  |  |
|  | *Cyathea ruiziana* |  |  |  | 32 |  |  |
|  | *Cyathea squamipes* |  |  | 15 | 18 | 330 |  |
|  | *Cyathea straminea* |  |  | 4 |  |  |  |
|  | *Cyathea suprastrigosa* |  | 9 |  |  |  |  |
|  | *Cyathea parvifolia* | 35 |  |  |  |  |  |
|  | *Sphaeropteris quindiuensis* |  |  | 5 |  |  |  |
| Dichapetalaceae | *Dichapetalum axillare* |  | 1 |  |  |  |  |
|  | *Dichapetalum bernalii* |  | 1 |  |  |  |  |
|  | *Stephanopodium aptotum* |  | 9 |  |  |  |  |
|  | *Stephanopodium peruvianum* |  |  | 1 |  |  |  |
|  | *Tapura amazonica* |  |  | 1 | 1 |  |  |
|  | *Tapura colombiana* |  | 5 |  |  |  |  |
|  | *Tapura peruviana* |  |  |  | 2 |  |  |
| Dicksoniaceae | *Dicksonia sellowiana* |  |  | 65 | 17 | 6 |  |
| Dilleniaceae | *Doliocarpus dentatus* |  | 5 |  |  |  |  |
|  | *Doliocarpus multiflorus* |  | 4 |  |  |  |  |
|  | *Pinzona coriacea* |  | 5 |  |  |  |  |
| Dipentodontaceae | *Perrottetia distichophylla* |  |  | 6 |  |  |  |
| Ebenaceae | *Diospyros artanthifolia* |  | 2 |  |  |  |  |
|  | *Diospyros inconstans* |  |  |  |  | 1 |  |
|  | *Diospyros nigra* |  | 1 |  |  |  |  |
|  | *Diospyros subrotata* |  |  | 1 |  |  |  |
|  | *Diospyros tessmannii* |  |  |  | 1 |  |  |
|  | *Diospyros vestita* |  | 27 |  |  |  |  |
| Elaeocarpaceae | *Crinodendron tucumanum* |  |  |  |  |  | 489 |
|  | *Sloanea brevispina* |  | 19 |  |  |  |  |
|  | *Sloanea calva* |  | 2 |  |  |  |  |
|  | *Sloanea durissima* |  |  | 1 |  |  |  |
|  | *Sloanea eichleri* |  |  | 6 |  |  |  |
|  | *Sloanea fendleriana* |  |  |  |  | 5 |  |
|  | *Sloanea floribunda* |  |  | 1 |  |  |  |
|  | *Sloanea fragrans* |  |  |  | 1 |  |  |
|  | *Sloanea grandiflora* |  |  | 2 |  |  |  |
|  | *Sloanea guianensis* |  | 1 |  |  |  |  |
|  | *Sloanea laxiflora* |  |  | 1 |  |  |  |
|  | *Sloanea medusula* |  |  | 2 |  |  |  |
|  | *Sloanea parvifructa* |  | 2 |  |  |  |  |
|  | *Sloanea ptariana* |  | 3 |  |  |  |  |
|  | *Sloanea pubescens* |  |  | 1 |  | 10 |  |
|  | *Sloanea rufa* |  |  | 5 |  | 1 |  |
|  | *Sloanea tuerckheimii* |  | 2 |  |  |  |  |
|  | *Vallea ecuadorensis* |  |  |  | 15 |  |  |
|  | *Vallea stipularis* |  |  | 27 | 23 |  | 24 |
| Ericaceae | *Bejaria aestuans* |  | 12 | 4 | 16 | 28 |  |
|  | *Bejaria resinosa* |  |  |  | 1 |  |  |
|  | *Cavendishia bracteata* |  |  | 3 | 3 | 85 |  |
|  | *Cavendishia pubescens* |  | 4 |  |  |  |  |
|  | *Demosthenesia mandonii* |  |  |  |  | 1 |  |
|  | *Diogenesia boliviana* |  |  |  |  | 1 |  |
|  | *Gaultheria reticulata* |  |  | 8 |  |  |  |
|  | *Psammisia roseiflora* |  |  | 2 |  |  |  |
| Erythroxylaceae | *Erythroxylum argentinum* |  |  |  |  |  | 28 |
|  | *Erythroxylum citrifolium* |  | 56 |  |  |  |  |
|  | *Erythroxylum deciduum* |  |  |  | 3 |  |  |
|  | *Erythroxylum macrophyllum* |  |  |  |  | 1 |  |
|  | *Erythroxylum raimondii* |  |  |  |  | 1 |  |
|  | *Erythroxylum subrotundum* |  |  |  |  | 47 |  |
| Escalloniaceae | *Escallonia millegrana* |  |  |  |  |  | 7 |
|  | *Escallonia myrtilloides* |  |  | 34 | 35 |  |  |
|  | *Escallonia paniculata* |  | 2 | 80 | 16 | 32 |  |
| Euphorbiaceae | *Acalypha diversifolia* |  |  | 2 |  |  |  |
|  | *Acalypha glandulosa* |  |  |  | 3 |  |  |
|  | *Acalypha macrostachya* |  |  | 1 | 1 |  |  |
|  | *Alchornea acutifolia* |  | 13 |  | 19 |  |  |
|  | *Alchornea anamariae* |  |  |  | 1 |  |  |
|  | *Alchornea brittonii* |  |  |  |  | 20 |  |
|  | *Alchornea coelophylla* |  | 61 |  |  |  |  |
|  | *Alchornea costaricensis* |  | 15 |  |  |  |  |
|  | *Alchornea glandulosa* |  | 14 | 118 |  | 92 |  |
|  | *Alchornea grandiflora* | 100 | 24 | 7 | 113 | 167 |  |
|  | *Alchornea grandis* |  |  | 6 | 20 |  |  |
|  | *Alchornea latifolia* |  |  | 20 | 8 | 8 |  |
|  | *Alchornea leptogyna* |  |  | 71 |  |  |  |
|  | *Alchornea lojaensis* |  |  | 36 |  |  |  |
|  | *Alchornea megalophylla* |  | 9 |  |  |  |  |
|  | *Alchornea pearcei* |  |  | 3 | 28 |  |  |
|  | *Alchornea triplinervia* |  | 10 | 14 | 2 | 86 |  |
|  | *Alchornea verticillata* |  | 64 |  |  |  |  |
|  | *Alchorneopsis floribunda* |  |  | 8 |  |  |  |
|  | *Aparisthmium cordatum* |  | 3 | 79 | 3 | 3 |  |
|  | *Caryodendron orinocense* |  |  | 60 |  |  |  |
|  | *Cnidoscolus vitifolius* |  |  |  |  |  | 28 |
|  | *Conceveiba parvifolia* |  | 8 |  |  |  |  |
|  | *Croton beetlei* |  |  |  |  | 1 |  |
|  | *Croton billbergianus* |  | 1 |  |  |  |  |
|  | *Croton floccosus* |  |  | 42 |  |  |  |
|  | *Croton killipianus* |  | 5 |  |  |  |  |
|  | *Croton lechleri* |  |  | 35 |  |  |  |
|  | *Croton lepidotus* |  |  | 1 |  |  |  |
|  | *Croton magdalenensis* |  | 36 | 5 |  |  |  |
|  | *Croton matourensis* |  |  | 1 |  |  |  |
|  | *Croton orbicularis* |  |  | 2 |  |  |  |
|  | *Croton piluliferus* |  |  |  |  |  | 315 |
|  | *Croton rimbachii* |  |  | 1 |  |  |  |
|  | *Croton rusbyi* |  |  |  |  | 7 |  |
|  | *Croton sampatik* |  |  |  |  | 5 |  |
|  | *Croton smithianus* |  | 153 |  |  |  |  |
|  | *Croton tessmannii* |  |  | 19 |  |  |  |
|  | *Hevea guianensis* |  |  | 1 | 8 |  |  |
|  | *Hura crepitans* |  | 1 |  | 1 | 3 |  |
|  | *Jatropha hieronymi* |  |  |  |  |  | 1 |
|  | *Mabea macbridei* |  |  | 4 |  | 20 |  |
|  | *Mabea nitida* |  |  | 1 |  |  |  |
|  | *Mabea speciosa* |  |  |  | 1 |  |  |
|  | *Maprounea guianensis* |  | 6 |  |  |  |  |
|  | *Nealchornea yapurensis* |  |  | 2 |  |  |  |
|  | *Omphalea diandra* |  | 4 |  |  | 2 |  |
|  | *Pachystroma longifolium* |  |  |  |  | 5 |  |
|  | *Pera arborea* |  | 51 |  |  |  |  |
|  | *Pera colombiana* |  | 6 |  |  |  |  |
|  | *Pseudosenefeldera inclinata* |  |  | 7 |  |  |  |
|  | *Sapium argutum* |  |  |  |  | 1 |  |
|  | *Sapium glandulosum* |  |  | 22 | 3 | 1 |  |
|  | *Sapium haematospermum* |  |  |  |  |  | 25 |
|  | *Sapium laurifolium* |  | 12 | 42 |  |  |  |
|  | *Sapium marmieri* |  |  | 79 | 4 |  |  |
|  | *Sapium stylare* | 4 | 20 | 38 |  |  |  |
|  | *Sebastiania brasiliensis* |  |  |  |  | 18 | 82 |
|  | *Senefeldera testiculata* |  | 1 |  |  |  |  |
|  | *Tetrorchidium andinum* |  | 2 | 14 |  | 1 |  |
|  | *Tetrorchidium euryphyllum* |  |  | 7 |  |  |  |
|  | *Tetrorchidium macrophyllum* |  |  | 16 |  |  |  |
|  | *Tetrorchidium rubrivenium* | 6 |  | 6 |  |  |  |
| Fabaceae | *Abarema barbouriana* |  | 8 |  |  |  |  |
|  | *Abarema callejasii* |  | 1 |  |  |  |  |
|  | *Abarema floribunda* |  | 2 |  |  |  |  |
|  | *Abarema jupunba* |  | 21 | 3 |  |  |  |
|  | *Abarema killipii* |  |  | 6 |  |  |  |
|  | *Abarema laeta* |  |  | 1 |  |  |  |
|  | *Abarema lehmannii* |  | 15 |  |  |  |  |
|  | *Acacia glomerosa* |  |  | 4 |  |  |  |
|  | *Albizia carbonaria* |  | 1 |  |  |  |  |
|  | *Albizia niopoides* |  |  |  |  | 1 |  |
|  | *Amburana cearensis* |  |  |  |  | 24 | 8 |
|  | *Anadenanthera colubrina* |  |  |  |  | 274 | 1158 |
|  | *Andira inermis* |  | 2 |  |  |  |  |
|  | *Andira macrothyrsa* |  |  | 4 |  |  |  |
|  | *Andira taurotesticulata* |  | 4 | 1 |  |  |  |
|  | *Apuleia leiocarpa* |  |  |  |  | 19 |  |
|  | *Bauhinia arborea* |  |  | 4 |  |  |  |
|  | *Bauhinia forficata* |  |  |  |  |  | 76 |
|  | *Bauhinia tuichiensis* |  |  |  |  | 9 |  |
|  | *Brownea coccinea* |  |  | 2 |  |  |  |
|  | *Brownea grandiceps* |  |  | 3 |  |  |  |
|  | *Brownea rosa-de-monte* |  | 10 |  |  |  |  |
|  | *Brownea ucayalina* |  |  | 6 |  |  |  |
|  | *Browneopsis macrofoliolata* |  |  | 9 |  |  |  |
|  | *Calliandra chulumania* |  |  |  |  | 2 |  |
|  | *Calliandra trinervia* |  |  | 4 |  |  |  |
|  | *Cassia moschata* |  | 2 |  |  |  |  |
|  | *Cedrelinga cateniformis* |  |  | 7 |  |  |  |
|  | *Clathrotropis brachypetala* |  | 4 |  |  |  |  |
|  | *Copaifera canime* |  | 5 |  |  |  |  |
|  | *Copaifera reticulata* |  |  |  |  | 63 |  |
|  | *Cynometra bauhiniifolia* |  | 22 |  |  |  |  |
|  | *Cynometra schottiana* |  | 11 |  |  |  |  |
|  | *Dahlstedtia hylobia* |  |  | 17 |  |  |  |
|  | *Dalbergia spruceana* |  |  |  |  | 1 |  |
|  | *Dialium guianense* |  | 19 |  |  |  |  |
|  | *Dimorphandra macrostachya* |  | 13 |  |  |  |  |
|  | *Diplotropis peruviana* |  |  |  |  | 3 |  |
|  | *Dipteryx oleifera* |  | 5 |  |  |  |  |
|  | *Dussia lehmannii* |  | 1 | 11 |  |  |  |
|  | *Dussia tessmannii* |  |  | 30 |  |  |  |
|  | *Dussia coriacea* | 1 |  |  |  |  |  |
|  | *Enterolobium contortisiliquum* |  | 4 |  |  | 3 | 44 |
|  | *Erythrina edulis* |  |  | 83 |  |  |  |
|  | *Erythrina falcata* |  |  |  |  |  | 33 |
|  | *Erythrina poeppigiana* |  |  | 1 |  |  |  |
|  | *Erythrina velutina* |  |  | 2 |  |  |  |
|  | *Gleditsia amorphoides* |  |  |  |  |  | 223 |
|  | *Holocalyx balansae* |  |  |  |  | 18 |  |
|  | *Hymenaea courbaril* |  |  |  |  | 33 |  |
|  | *Hymenaea oblongifolia* |  | 1 |  |  |  |  |
|  | *Inga acreana* |  |  | 56 |  | 2 |  |
|  | *Inga acrocephala* |  | 8 |  |  |  |  |
|  | *Inga acuminata* |  | 1 | 1 |  |  |  |
|  | *Inga alata* |  |  | 5 |  |  |  |
|  | *Inga alatocarpa* |  |  | 4 |  |  |  |
|  | *Inga alba* |  | 11 | 1 |  | 21 |  |
|  | *Inga angustifolia* |  |  | 2 |  |  |  |
|  | *Inga auristellae* |  |  | 1 |  |  |  |
|  | *Inga bourgonii* |  |  | 6 |  |  |  |
|  | *Inga capitata* |  |  | 4 |  |  |  |
|  | *Inga carinata* |  |  | 2 |  |  |  |
|  | *Inga chartacea* |  |  | 2 |  |  |  |
|  | *Inga cordatoalata* |  |  | 1 |  |  |  |
|  | *Inga coruscans* |  |  | 3 |  |  |  |
|  | *Inga cylindrica* |  |  |  |  | 93 |  |
|  | *Inga densiflora* |  |  | 3 |  |  |  |
|  | *Inga edulis* |  | 1 | 15 |  |  | 71 |
|  | *Inga extra-nodis* |  |  | 12 |  |  |  |
|  | *Inga fendleriana* |  |  | 1 |  | 21 |  |
|  | *Inga glomeriflora* |  |  | 13 |  |  |  |
|  | *Inga gracilior* |  |  | 3 |  |  |  |
|  | *Inga heterophylla* |  | 5 |  | 1 | 3 |  |
|  | *Inga ilta* |  |  | 9 |  |  |  |
|  | *Inga lallensis* |  | 3 |  |  |  |  |
|  | *Inga laurina* |  |  | 4 |  | 1 |  |
|  | *Inga leiocalycina* |  |  | 6 |  |  |  |
|  | *Inga macrophylla* |  | 1 |  |  |  |  |
|  | *Inga marginata* |  |  | 25 |  | 3 | 191 |
|  | *Inga multicaulis* |  |  | 12 |  |  |  |
|  | *Inga multijuga* |  | 19 |  |  |  |  |
|  | *Inga multinervis* |  |  | 41 |  | 17 |  |
|  | *Inga nobilis* |  | 14 | 1 |  | 2 |  |
|  | *Inga oerstediana* |  | 2 | 96 |  |  |  |
|  | *Inga pezizifera* |  | 12 |  |  |  |  |
|  | *Inga pruriens* |  |  | 1 |  |  |  |
|  | *Inga psittacorum* |  |  | 12 |  |  |  |
|  | *Inga punctata* |  |  | 20 |  | 2 |  |
|  | *Inga ruiziana* |  |  | 14 |  |  |  |
|  | *Inga saltensis* |  |  |  |  |  | 33 |
|  | *Inga sapindoides* |  | 2 | 4 |  | 1 |  |
|  | *Inga sertulifera* |  | 2 |  |  |  |  |
|  | *Inga sierrae* |  | 9 |  |  |  |  |
|  | *Inga silanchensis* |  |  | 1 |  |  |  |
|  | *Inga spectabilis* |  |  | 6 |  |  |  |
|  | *Inga stipulacea* |  |  | 1 |  |  |  |
|  | *Inga striata* |  |  |  |  | 22 |  |
|  | *Inga suaveolens* |  |  | 2 |  |  |  |
|  | *Inga thibaudiana* |  | 19 | 10 |  |  |  |
|  | *Inga umbellifera* |  | 2 | 2 |  |  |  |
|  | *Inga velutina* |  |  | 1 |  |  |  |
|  | *Inga venusta* |  | 13 |  |  |  |  |
|  | *Inga villosissima* |  | 6 |  |  |  |  |
|  | *Inga vismiifolia* |  |  | 6 |  |  |  |
|  | *Inga ynga* |  |  |  |  | 12 |  |
|  | *Lachesiodendron viridiflorum* |  |  |  |  | 28 | 1 |
|  | *Libidibia paraguariensis* |  |  |  |  |  | 11 |
|  | *Lonchocarpus lilloi* |  |  |  |  |  | 141 |
|  | *Lonchocarpus macrophyllus* |  | 8 |  |  |  |  |
|  | *Machaerium acutifolium* |  |  |  |  | 5 |  |
|  | *Machaerium arboreum* |  | 1 |  |  |  |  |
|  | *Machaerium biovulatum* |  | 5 |  |  |  |  |
|  | *Machaerium capote* |  | 1 |  |  |  |  |
|  | *Machaerium floribundum* |  |  | 1 |  | 1 |  |
|  | *Machaerium hirtum* |  |  |  |  | 23 |  |
|  | *Machaerium microphyllum* |  | 1 |  |  |  |  |
|  | *Machaerium nyctitans* |  |  |  |  | 397 |  |
|  | *Macrolobium archeri* |  |  | 4 |  |  |  |
|  | *Macrolobium colombianum* |  | 8 |  |  |  |  |
|  | *Macrolobium gracile* |  | 13 |  |  |  |  |
|  | *Macrolobium pittieri* |  | 1 |  |  |  |  |
|  | *Myrocarpus venezuelensis* |  |  |  |  | 18 |  |
|  | *Myroxylon peruiferum* |  |  |  |  | 55 | 158 |
|  | *Neustanthus phaseoloides* |  | 1 |  |  |  |  |
|  | *Ormosia antioquensis* |  | 2 |  |  |  |  |
|  | *Ormosia corcovada* |  | 5 |  |  |  |  |
|  | *Ormosia cuatrecasasii* |  | 29 |  |  |  |  |
|  | *Ormosia paraensis* |  | 4 |  |  |  |  |
|  | *Parapiptadenia excelsa* |  |  |  |  | 71 | 1022 |
|  | *Parkia balslevii* |  |  | 1 |  |  |  |
|  | *Parkia multijuga* |  |  | 4 |  |  |  |
|  | *Parkia nitida* |  |  | 2 |  |  |  |
|  | *Parkia velutina* |  |  | 3 |  |  |  |
|  | *Peltogyne paniculata* |  | 1 |  |  |  |  |
|  | *Pentaclethra macroloba* |  | 8 |  |  |  |  |
|  | *Piptadenia buchtienii* |  |  |  |  | 9 |  |
|  | *Piptadenia gonoacantha* |  |  |  |  | 9 |  |
|  | *Platymiscium pinnatum* |  | 3 |  |  | 15 |  |
|  | *Platymiscium pubescens* |  |  |  |  | 3 |  |
|  | *Poincianella pluviosa* |  |  |  |  |  | 10 |
|  | *Prioria copaifera* |  | 13 |  |  |  |  |
|  | *Pterocarpus officinalis* |  | 12 |  |  |  |  |
|  | *Pterocarpus rhorii* |  |  |  | 6 |  |  |
|  | *Pterogyne nitens* |  |  |  |  |  | 5 |
|  | *Samanea saman* |  | 2 |  |  |  |  |
|  | *Schizolobium parahyba* |  | 1 | 1 |  |  |  |
|  | *Schnella guianensis* |  | 3 |  |  |  |  |
|  | *Senegalia loretensis* |  |  |  |  | 57 |  |
|  | *Senegalia martiusiana* |  |  |  |  | 2 |  |
|  | *Senegalia parviceps* |  |  |  |  | 2 |  |
|  | *Senegalia polyphylla* |  |  |  |  | 70 |  |
|  | *Senegalia praecox* |  |  |  |  |  | 4 |
|  | *Senegalia rhytidocarpa* |  |  |  |  | 3 |  |
|  | *Senegalia tenuifolia* |  |  |  |  | 10 |  |
|  | *Senna spectabilis* |  |  |  |  |  | 26 |
|  | *Stryphnodendron microstachyum* |  | 5 |  |  |  |  |
|  | *Styphnolobium sporadicum* |  | 2 |  |  |  |  |
|  | *Swartzia amplifolia* |  | 2 |  |  |  |  |
|  | *Swartzia haughtii* |  | 1 |  |  |  |  |
|  | *Swartzia macrophylla* |  | 4 |  |  |  |  |
|  | *Swartzia macrosema* |  |  | 3 |  |  |  |
|  | *Swartzia myrtifolia* |  | 16 |  |  |  |  |
|  | *Swartzia oraria* |  | 14 |  |  |  |  |
|  | *Swartzia panamensis* |  | 2 |  |  |  |  |
|  | *Swartzia piarensis* |  | 28 |  |  |  |  |
|  | *Swartzia robiniifolia* |  | 1 |  |  |  |  |
|  | *Swartzia schultesii* |  | 2 |  |  |  |  |
|  | *Swartzia simplex* |  | 11 |  |  |  |  |
|  | *Sweetia fruticosa* |  |  |  |  | 57 |  |
|  | *Tachigali inconspicua* |  |  | 3 |  |  |  |
|  | *Tachigali paniculata* |  |  | 3 |  |  |  |
|  | *Tachigali paraensis* |  |  | 1 |  |  |  |
|  | *Tipuana tipu* |  |  |  |  |  | 136 |
|  | *Vachellia caven* |  |  |  |  |  | 6 |
|  | *Zygia coccinea* |  |  | 2 |  |  |  |
|  | *Zygia latifolia* |  |  | 1 |  |  |  |
|  | *Zygia picramnioides* |  | 18 |  |  |  |  |
| Fagaceae | *Quercus humboldtii* |  | 436 |  |  |  |  |
| Gentianaceae | *Macrocarpaea maguirei* |  |  |  | 1 |  |  |
|  | *Symbolanthus anomalus* |  |  | 1 |  |  |  |
|  | *Tachia parviflora* |  | 2 |  |  |  |  |
| Gesneriaceae | *Besleria solanoides* |  |  | 3 |  |  |  |
| Goupiaceae | *Goupia glabra* |  | 2 |  |  |  |  |
| Hamamelidaceae | *Matudaea colombiana* |  | 89 |  |  |  |  |
| Heliotropiaceae | *Tournefortia brevilobata* |  |  | 2 |  |  |  |
|  | *Tournefortia fuliginosa* |  |  | 17 |  |  |  |
|  | *Tournefortia scabrida* |  |  | 5 |  |  |  |
| Herndiaceae | *Hernandia didymantha* |  | 1 |  |  |  |  |
| Humiriaceae | *Humiriastrum colombianum* |  | 13 |  |  |  |  |
|  | *Humiriastrum diguense* |  |  | 2 |  |  |  |
| Hypericaceae | *Vismia baccifera* |  | 29 | 12 |  |  |  |
|  | *Vismia cavanillesiana* |  |  | 5 |  |  |  |
|  | *Vismia glabra* |  |  |  |  | 4 |  |
|  | *Vismia gracilis* |  |  | 5 |  |  |  |
|  | *Vismia laevis* |  | 58 |  |  |  |  |
|  | *Vismia lateriflora* |  |  | 4 |  |  |  |
|  | *Vismia lauriformis* |  | 2 | 6 |  |  |  |
|  | *Vismia macrophylla* |  | 15 |  |  |  |  |
|  | *Vismia mandurr* |  |  |  | 11 |  |  |
|  | *Vismia rusbyi* |  |  |  |  | 1 |  |
|  | *Vismia tomentosa* |  |  | 1 | 2 |  |  |
| Icacinaceae | *Calatola costaricensis* |  | 4 | 88 |  |  |  |
| Juglandaceae | *Alfaroa colombiana* |  | 6 |  |  |  |  |
|  | *Juglans australis* |  |  |  |  |  | 335 |
|  | *Juglans boliviana* |  |  |  |  | 13 |  |
|  | *Juglans neotropica* |  |  | 39 |  |  |  |
| Lacistemataceae | *Lacistema aggregatum* |  | 7 | 1 |  | 1 |  |
|  | *Lacistema nena* |  |  | 3 |  |  |  |
|  | *Lozania klugii* |  |  | 6 |  |  |  |
|  | *Lozania mutisiana* |  | 36 | 1 |  |  |  |
| Lamiaceae | *Aegiphila alba* |  | 4 | 34 |  |  |  |
|  | *Aegiphila bogotensis* |  |  | 14 |  |  |  |
|  | *Aegiphila cuatrecasasii* |  |  | 19 |  |  |  |
|  | *Aegiphila integrifolia* |  | 2 | 4 |  |  |  |
|  | *Aegiphila monticola* |  |  | 1 |  |  |  |
|  | *Aegiphila multiflora* |  |  |  | 9 | 43 |  |
|  | *Aegiphila saltensis* |  |  |  | 17 |  | 18 |
|  | *Hyptidendron arboreum* |  | 6 | 1 |  |  |  |
|  | *Lepechinia mollis* |  |  | 2 |  |  |  |
|  | *Vitex capitata* |  | 2 |  |  |  |  |
|  | *Vitex colombiensis* |  | 3 |  |  |  |  |
|  | *Vitex cymosa* |  |  | 1 |  | 1 |  |
| Lauraceae | *Aiouea dubia* | 1 | 8 | 7 |  |  |  |
|  | *Aiouea montana* |  | 13 | 15 |  | 1 |  |
|  | *Aiouea napoensis* |  |  | 2 |  |  |  |
|  | *Aniba coto* |  |  | 18 |  | 6 |  |
|  | *Aniba guianensis* |  |  | 10 |  | 24 |  |
|  | *Aniba hostmanniana* |  |  | 5 |  |  |  |
|  | *Aniba muca* |  | 10 | 28 |  | 10 |  |
|  | *Aniba panurensis* |  |  | 4 |  |  |  |
|  | *Aniba perutilis* |  | 44 |  |  |  |  |
|  | *Aniba puchury-minor* |  | 2 |  |  |  |  |
|  | *Aniba riparia* |  |  | 9 |  |  |  |
|  | *Aniba robusta* |  | 3 |  |  |  |  |
|  | *Aniba cinnamomiflora* | 1 |  |  |  |  |  |
|  | *Beilschmiedia alloiophylla* |  |  | 6 |  |  |  |
|  | *Beilschmiedia costaricensis* |  | 171 | 8 |  |  |  |
|  | *Beilschmiedia latifolia* | 4 | 3 |  | 26 | 16 |  |
|  | *Beilschmiedia pendula* |  | 12 | 5 |  |  |  |
|  | *Beilschmiedia tovarensis* | 82 | 2 | 20 |  | 59 |  |
|  | *Caryodaphnopsis tomentosa* |  |  | 1 |  |  |  |
|  | *Chlorocardium venenosum* |  | 1 |  |  |  |  |
|  | *Cinnamomum triplinerve* |  |  | 1 |  |  |  |
|  | *Cryptocarya aschersoniana* |  |  | 1 |  |  |  |
|  | *Cryptocarya guianensis* |  |  | 1 |  |  |  |
|  | *Endlicheria anomala* |  |  | 1 |  |  |  |
|  | *Endlicheria aurea* |  |  |  |  | 4 |  |
|  | *Endlicheria canescens* |  |  | 4 |  | 11 |  |
|  | *Endlicheria colombiana* |  | 1 |  |  |  |  |
|  | *Endlicheria formosa* |  |  | 7 |  |  |  |
|  | *Endlicheria gracilis* |  |  | 1 |  |  |  |
|  | *Endlicheria griseosericea* |  |  | 34 |  |  |  |
|  | *Endlicheria lorastemon* |  |  | 2 |  |  |  |
|  | *Endlicheria metallica* |  | 2 |  |  |  |  |
|  | *Endlicheria oreocola* |  |  | 6 |  |  |  |
|  | *Endlicheria pubescente* |  |  | 12 |  |  |  |
|  | *Endlicheria pyriformis* |  |  | 1 |  |  |  |
|  | *Endlicheria racemosa* |  |  | 1 |  |  |  |
|  | *Endlicheria ruforamula* |  |  | 8 |  |  |  |
|  | *Endlicheria sericea* |  |  | 26 |  |  |  |
|  | *Endlicheria tschudyana* |  | 1 |  |  |  |  |
|  | *Licaria applanata* |  | 13 | 9 |  |  |  |
|  | *Licaria cannella* |  |  | 10 |  |  |  |
|  | *Licaria guianensis* |  |  | 1 |  |  |  |
|  | *Licaria pucheri* |  |  |  |  | 19 |  |
|  | *Licaria subsessilis* |  |  | 14 |  |  |  |
|  | *Licaria terminalis* |  |  | 3 |  |  |  |
|  | *Licaria triandra* |  |  | 4 |  |  |  |
|  | *Nectandra acuminata* |  |  |  | 4 |  |  |
|  | *Nectandra acutifolia* |  | 1 | 32 |  | 13 |  |
|  | *Nectandra cissiflora* |  |  | 1 |  | 75 |  |
|  | *Nectandra coeloclada* |  |  | 5 |  |  |  |
|  | *Nectandra crassiloba* |  |  | 2 |  |  |  |
|  | *Nectandra cuspidata* |  |  |  | 31 | 50 | 155 |
|  | *Nectandra guadaripo* |  |  | 4 |  |  |  |
|  | *Nectandra hihua* |  |  |  |  | 1 |  |
|  | *Nectandra laurel* |  | 2 | 55 |  | 6 |  |
|  | *Nectandra lineata* |  |  | 17 |  |  |  |
|  | *Nectandra lineatifolia* |  |  | 7 |  |  |  |
|  | *Nectandra megapotamica* |  |  |  |  | 2 |  |
|  | *Nectandra membranacea* |  |  | 56 |  |  |  |
|  | *Nectandra obtusata* |  |  | 16 |  |  |  |
|  | *Nectandra olida* |  |  |  | 41 |  |  |
|  | *Nectandra oppositifolia* |  |  | 2 |  |  |  |
|  | *Nectandra paucinervia* |  |  | 1 |  |  |  |
|  | *Nectandra pearcei* |  |  | 2 |  |  |  |
|  | *Nectandra purpurea* |  |  | 5 |  |  |  |
|  | *Nectandra reticulata* |  | 7 | 23 | 1 |  |  |
|  | *Nectandra subbullata* |  |  | 86 |  |  |  |
|  | *Nectandra utilis* |  |  |  | 36 |  |  |
|  | *Nectandra viburnoides* |  |  | 1 | 1 |  |  |
|  | *Nectandra reticulata* | 2 |  |  |  |  |  |
|  | *Ocotea aciphylla* | 5 |  | 24 |  | 87 |  |
|  | *Ocotea acutifolia* |  |  |  |  | 1 |  |
|  | *Ocotea albida* |  |  |  |  | 139 |  |
|  | *Ocotea amazonica* |  |  | 1 |  |  |  |
|  | *Ocotea andina* |  |  | 8 |  |  |  |
|  | *Ocotea architectorum* |  |  | 2 |  |  |  |
|  | *Ocotea arnottiana* |  |  | 3 |  |  |  |
|  | *Ocotea atirrensis* |  |  | 3 |  |  |  |
|  | *Ocotea balanocarpa* |  | 79 |  |  |  |  |
|  | *Ocotea benthamiana* |  |  | 31 |  |  |  |
|  | *Ocotea bofo* |  |  | 5 |  | 55 |  |
|  | *Ocotea caesifolia* |  |  |  |  | 9 |  |
|  | *Ocotea caniflora* |  |  | 4 |  |  |  |
|  | *Ocotea cernua* |  |  | 23 |  | 3 |  |
|  | *Ocotea comata* |  |  |  |  | 193 |  |
|  | *Ocotea corymbosa* |  |  | 4 |  |  |  |
|  | *Ocotea cuneifolia* |  |  | 6 |  |  |  |
|  | *Ocotea cuprea* |  |  |  |  | 34 |  |
|  | *Ocotea duquei* |  | 1 |  |  |  |  |
|  | *Ocotea floribunda* |  | 33 | 39 |  | 2 |  |
|  | *Ocotea glabriflora* |  |  |  | 57 |  |  |
|  | *Ocotea guianensis* |  | 14 |  |  |  |  |
|  | *Ocotea heterochroma* |  |  | 1 |  |  |  |
|  | *Ocotea infrafoveolata* |  |  | 140 |  |  |  |
|  | *Ocotea insularis* |  | 2 | 65 |  |  |  |
|  | *Ocotea javitensis* |  |  | 17 |  |  |  |
|  | *Ocotea jelskii* |  | 6 |  |  | 2 |  |
|  | *Ocotea longifolia* |  |  | 5 |  | 2 |  |
|  | *Ocotea macrophylla* |  | 16 |  |  |  |  |
|  | *Ocotea micrantha* |  |  |  |  | 21 |  |
|  | *Ocotea oblonga* |  |  | 4 |  |  |  |
|  | *Ocotea oblongoobovata* |  |  | 1 |  |  |  |
|  | *Ocotea olivacea* |  |  |  |  | 3 |  |
|  | *Ocotea pacifica* |  |  | 1 |  |  |  |
|  | *Ocotea pichinchensis* |  |  | 1 |  |  |  |
|  | *Ocotea porphyria* |  |  |  |  |  | 1141 |
|  | *Ocotea puberula* | 1 |  |  |  |  | 695 |
|  | *Ocotea quixos* |  |  | 5 |  |  |  |
|  | *Ocotea rotundata* |  |  | 4 |  |  |  |
|  | *Ocotea rugosa* |  |  | 1 |  |  |  |
|  | *Ocotea sericea* |  | 9 | 2 |  |  |  |
|  | *Ocotea smithiana* |  | 2 | 1 |  |  |  |
|  | *Ocotea splendens* |  |  | 1 |  |  |  |
|  | *Ocotea subrutilans* |  |  | 5 |  |  |  |
|  | *Ocotea tabacifolia* |  |  |  | 1 |  |  |
|  | *Ocotea tessmannii* |  |  | 2 |  |  |  |
|  | *Ocotea tomentosa* |  |  | 1 |  |  |  |
|  | *Ocotea ucayalensis* |  |  | 2 |  |  |  |
|  | *Ocotea weberbaueri* |  |  |  |  | 8 |  |
|  | *Ocotea calophylla* | 1 |  |  |  |  |  |
|  | *Ocotea karsteniana* | 10 |  |  |  |  |  |
|  | *Ocotea macropoda* | 26 |  |  |  |  |  |
|  | *Persea americana* |  | 10 | 10 |  |  | 14 |
|  | *Persea areolatocostae* |  | 22 | 6 | 1 | 10 |  |
|  | *Persea brevipes* |  |  | 1 | 2 |  |  |
|  | *Persea caerulea* |  |  | 50 |  |  |  |
|  | *Persea corymbosa* |  |  |  | 10 |  |  |
|  | *Persea cuneata* |  | 9 |  |  |  |  |
|  | *Persea ferruginea* |  |  | 49 | 23 |  |  |
|  | *Persea hexanthera* |  |  | 17 |  |  |  |
|  | *Persea mutisii* |  | 4 | 10 | 60 |  |  |
|  | *Persea nudigemma* |  |  |  | 2 |  |  |
|  | *Persea palustris* |  |  | 1 |  |  |  |
|  | *Persea peruviana* | 5 |  |  |  | 4 |  |
|  | *Persea pseudofasciculata* |  |  | 16 |  |  |  |
|  | *Persea raimondii* |  |  | 2 |  |  |  |
|  | *Persea rigens* |  | 15 | 7 |  |  |  |
|  | *Persea sericea* |  |  | 2 |  |  |  |
|  | *Persea sphaerocarpa* |  |  |  |  | 6 |  |
|  | *Persea subcordata* |  | 1 | 7 |  | 4 |  |
|  | *Persea tosca* |  |  | 1 |  |  |  |
|  | *Petrea maynensis* |  |  | 1 |  |  |  |
|  | *Pleurothyrium cinereum* |  |  | 2 |  |  |  |
|  | *Pleurothyrium glabrifolium* |  |  | 1 |  |  |  |
|  | *Pleurothyrium glabritepalum* |  |  | 2 |  |  |  |
|  | *Pleurothyrium insigne* |  |  | 2 |  |  |  |
|  | *Pleurothyrium obovatum* |  |  | 1 |  |  |  |
|  | *Pleurothyrium trianae* |  |  | 6 |  | 37 |  |
|  | *Rhodostemonodaphne grandis* |  | 3 | 4 |  |  |  |
|  | *Rhodostemonodaphne kunthiana* |  | 22 | 22 |  | 36 |  |
|  | *Rhodostemonodaphne laxa* |  | 4 |  |  |  |  |
|  | *Williamodendron glaucophyllum* |  | 1 |  |  |  |  |
| Lecythidaceae | *Cariniana estrellensis* |  |  |  |  | 31 |  |
|  | *Cariniana ianeirensis* |  |  |  |  | 9 |  |
|  | *Cariniana pyriformis* |  | 2 |  |  |  |  |
|  | *Couroupita guianensis* |  | 4 |  |  |  |  |
|  | *Eschweilera albiflora* |  | 1 |  |  |  |  |
|  | *Eschweilera andina* |  |  | 2 |  |  |  |
|  | *Eschweilera antioquensis* |  | 78 |  |  |  |  |
|  | *Eschweilera baguensis* |  |  | 8 |  |  |  |
|  | *Eschweilera bracteosa* |  |  | 1 |  |  |  |
|  | *Eschweilera calyculata* |  | 6 |  |  |  |  |
|  | *Eschweilera caudiculata* |  |  | 29 |  |  |  |
|  | *Eschweilera coriacea* |  | 37 | 20 | 4 |  |  |
|  | *Eschweilera integrifolia* |  | 4 |  |  |  |  |
|  | *Eschweilera juruensis* |  |  | 3 |  |  |  |
|  | *Eschweilera microcalyx* |  | 7 |  |  |  |  |
|  | *Eschweilera rimbachii* |  |  | 6 |  |  |  |
|  | *Eschweilera rufifolia* |  |  | 4 |  |  |  |
|  | *Eschweilera sessilis* |  | 17 | 5 |  |  |  |
|  | *Grias neuberthii* |  |  | 60 |  |  |  |
|  | *Grias peruviana* |  |  | 220 | 1 |  |  |
|  | *Gustavia angustifolia* |  |  | 1 |  |  |  |
|  | *Gustavia dodsonii* |  |  | 8 |  |  |  |
|  | *Gustavia dubia* |  | 6 |  |  |  |  |
|  | *Gustavia gracillima* |  | 40 |  |  |  |  |
|  | *Gustavia longifolia* |  |  | 6 |  |  |  |
|  | *Gustavia macarenensis* |  |  | 12 |  |  |  |
|  | *Gustavia speciosa* |  | 8 |  |  |  |  |
|  | *Gustavia superba* |  | 24 |  |  |  |  |
|  | *Lecythis ampla* |  | 2 |  |  |  |  |
| Loganiaceae | *Strychnos bredemeyeri* |  |  | 1 |  |  |  |
|  | *Strychnos panamensis* |  | 2 |  |  |  |  |
|  | *Strychnos toxifera* |  | 1 |  |  |  |  |
| Loranthaceae | *Gaiadendron punctatum* |  |  | 55 | 7 | 1 |  |
| Magnoliaceae | *Magnolia amazonica* |  |  |  | 3 |  |  |
|  | *Magnolia espinalii* |  | 3 |  |  |  |  |
|  | *Magnolia gilbertoi* |  |  |  | 1 |  |  |
|  | *Magnolia madidiensis* |  |  |  |  | 5 |  |
|  | *Magnolia mashpi* |  |  | 5 |  |  |  |
|  | *Magnolia palandana* |  |  | 3 |  |  |  |
|  | *Magnolia rimachii* |  |  | 2 |  |  |  |
|  | *Magnolia silvioi* |  | 2 |  |  |  |  |
|  | *Magnolia urraoensis* |  | 13 |  |  |  |  |
|  | *Magnolia yarumalensis* |  | 5 |  | 1 |  |  |
| Malpighiaceae | *Alicia macrodisca* |  |  |  |  | 1 |  |
|  | *Bunchosia argentea* |  |  | 7 |  |  |  |
|  | *Byrsonima arthropoda* |  | 11 | 1 |  | 1 |  |
|  | *Byrsonima garcibarrigae* |  | 2 |  |  |  |  |
|  | *Byrsonima homeieri* |  |  | 2 |  |  |  |
|  | *Byrsonima putumayensis* |  |  | 1 |  |  |  |
|  | *Byrsonima spicata* |  | 10 |  |  | 11 |  |
|  | *Diplopterys pubipetala* |  |  |  |  | 1 |  |
|  | *Heteropterys laurifolia* |  |  |  |  | 1 |  |
|  | *Mascagnia divaricata* |  |  |  |  | 1 |  |
|  | *Pterandra colombiana* |  | 2 |  |  |  |  |
|  | *Ptilochaeta nudipes* |  |  |  |  |  | 2 |
|  | *Tetrapterys mortonii* |  |  | 1 |  |  |  |
| Malvaceae | *Apeiba glabra* |  | 11 | 8 |  |  |  |
|  | *Apeiba membranacea* |  |  | 21 | 10 |  |  |
|  | *Catostemma digitata* |  | 3 |  |  |  |  |
|  | *Ceiba boliviana* |  |  |  |  | 50 |  |
|  | *Ceiba chodatii* |  |  |  |  |  | 109 |
|  | *Ceiba pentandra* |  | 9 |  |  |  |  |
|  | *Ceiba samauma* |  |  |  |  | 3 |  |
|  | *Ceiba speciosa* |  |  |  |  | 27 |  |
|  | *Eriotheca globosa* |  | 3 |  |  |  |  |
|  | *Eriotheca macrophylla* |  |  |  |  | 10 |  |
|  | *Eriotheca roseorum* |  |  |  |  |  | 6 |
|  | *Guazuma ulmifolia* |  | 1 |  |  |  |  |
|  | *Hampea punctulata* |  | 10 |  |  |  |  |
|  | *Heliocarpus americanus* |  | 1 | 47 | 3 |  |  |
|  | *Heliocarpus popayanensis* |  |  |  |  | 3 | 163 |
|  | *Luehea cymulosa* |  |  | 1 |  |  |  |
|  | *Luehea grandiflora* |  |  |  |  |  | 24 |
|  | *Luehea seemannii* |  | 14 |  |  |  |  |
|  | *Luehea splendens* |  |  |  |  | 2 |  |
|  | *Matisia bicolor* |  |  |  | 36 |  |  |
|  | *Matisia bolivarii* |  |  | 4 |  |  |  |
|  | *Matisia bracteolosa* |  |  | 4 | 4 |  |  |
|  | *Matisia castano* |  |  | 11 |  |  |  |
|  | *Matisia coloradum* |  |  |  | 3 |  |  |
|  | *Matisia cordata* |  | 1 |  |  |  |  |
|  | *Matisia giacomettoi* |  |  | 4 |  |  |  |
|  | *Matisia grandifolia* |  |  | 1 | 19 |  |  |
|  | *Matisia idroboi* |  | 4 | 1 |  |  |  |
|  | *Matisia intricata* |  | 6 |  | 1 |  |  |
|  | *Matisia longipes* |  |  | 2 |  |  |  |
|  | *Matisia malacocalyx* |  |  | 2 |  |  |  |
|  | *Matisia obliquifolia* |  |  | 2 |  |  |  |
|  | *Matisia ochrocalyx* |  |  | 2 |  |  |  |
|  | *Matisia soegengii* |  |  | 8 |  |  |  |
|  | *Mollia gracilis* |  |  | 4 |  |  |  |
|  | *Ochroma pyramidale* |  |  | 8 |  |  |  |
|  | *Pachira punga-schunkei* |  |  | 7 |  |  |  |
|  | *Pentaplaris doroteae* |  | 7 |  |  |  |  |
|  | *Phragmotheca ecuadorensis* |  |  | 3 |  |  |  |
|  | *Pseudobombax argentinum* |  |  |  |  |  | 33 |
|  | *Pseudobombax septenatum* |  | 1 |  |  | 26 |  |
|  | *Pterygota amazonica* |  |  |  |  | 19 |  |
|  | *Quararibea asterolepis* |  | 12 |  |  |  |  |
|  | *Quararibea cogolloi* |  | 1 |  |  |  |  |
|  | *Quararibea wittii* |  |  | 3 |  |  |  |
|  | *Spirotheca rosea* |  | 3 | 4 |  |  |  |
|  | *Sterculia apeibophylla* |  |  | 1 |  |  |  |
|  | *Sterculia apetala* |  |  | 3 |  |  |  |
|  | *Sterculia colombiana* |  |  | 3 | 3 |  |  |
|  | *Sterculia excelsa* |  | 3 |  |  |  |  |
|  | *Sterculia frondosa* |  |  |  | 7 |  |  |
|  | *Sterculia tessmannii* |  |  | 5 |  |  |  |
|  | *Tartagalia roseorum* |  |  |  |  |  | 1 |
|  | *Theobroma bicolor* |  | 1 |  |  |  |  |
|  | *Theobroma cacao* |  | 1 | 5 | 4 |  |  |
|  | *Theobroma gileri* |  |  | 15 |  |  |  |
|  | *Theobroma glaucum* |  | 3 |  |  |  |  |
|  | *Theobroma subincanum* |  |  | 2 |  |  |  |
| Melastomataceae | *Axinaea affinis* |  |  | 2 |  |  |  |
|  | *Axinaea alata* |  |  |  |  | 19 |  |
|  | *Axinaea glandulosa* |  |  |  |  | 50 |  |
|  | *Axinaea lanceolata* |  |  |  |  | 4 |  |
|  | *Axinaea macrophylla* |  |  | 138 | 163 |  |  |
|  | *Axinaea oblongifolia* |  |  | 1 |  |  |  |
|  | *Axinaea pennellii* |  |  |  | 118 |  |  |
|  | *Axinaea quitensis* |  |  | 42 |  |  |  |
|  | *Axinaea scutigera* |  |  | 3 |  |  |  |
|  | *Axinaea tomentosa* |  |  |  | 19 |  |  |
|  | *Axinaea grandifolia* | 13 |  |  |  |  |  |
|  | *Bellucia gracilis* |  |  |  |  | 5 |  |
|  | *Bellucia grossularioides* |  | 44 |  |  |  |  |
|  | *Bellucia pentamera* |  | 9 |  |  |  |  |
|  | *Blakea albertiae* |  | 4 |  |  |  |  |
|  | *Blakea discolor* |  | 1 |  |  |  |  |
|  | *Blakea eriocalyx* |  |  | 1 |  |  |  |
|  | *Blakea holtonii* |  | 17 |  |  |  |  |
|  | *Blakea multiflora* |  |  |  |  | 76 |  |
|  | *Blakea quadrangularis* |  | 16 |  |  |  |  |
|  | *Blakea rotundifolia* |  |  | 7 |  |  |  |
|  | *Blakea subconnata* |  |  | 2 |  |  |  |
|  | *Centronia laurifolia* |  |  | 8 |  |  |  |
|  | *Clidemia dentata* |  |  | 1 |  |  |  |
|  | *Clidemia septuplinervia* |  |  | 14 |  |  |  |
|  | *Conostegia centronioides* |  |  | 1 |  |  |  |
|  | *Conostegia montana* |  | 5 | 3 |  |  |  |
|  | *Conostegia superba* |  |  | 3 |  |  |  |
|  | *Graffenrieda cucullata* |  |  | 22 | 33 |  |  |
|  | *Graffenrieda emarginata* |  | 2 | 75 |  | 4 |  |
|  | *Graffenrieda galeottii* |  | 10 | 2 |  |  |  |
|  | *Graffenrieda harlingii* |  |  | 49 |  |  |  |
|  | *Graffenrieda intermedia* |  |  | 2 |  |  |  |
|  | *Graffenrieda latifolia* |  | 2 |  |  |  |  |
|  | *Graffenrieda limbata* |  |  |  |  | 2 |  |
|  | *Graffenrieda micrantha* |  | 11 |  |  |  |  |
|  | *Henriettea trachyphylla* |  | 1 |  |  |  |  |
|  | *Henriettea verrucosa* |  |  | 1 |  |  |  |
|  | *Meriania acostae* |  |  | 1 |  |  |  |
|  | *Meriania antioquiensis* |  | 2 |  |  |  |  |
|  | *Meriania brachycera* | 1 |  |  |  |  |  |
|  | *Meriania brittoniana* |  |  |  |  | 8 |  |
|  | *Meriania colombiana* |  |  | 2 |  |  |  |
|  | *Meriania cuzcoana* |  |  |  | 5 |  |  |
|  | *Meriania drakei* |  |  | 3 |  |  |  |
|  | *Meriania franciscana* |  |  | 17 |  |  |  |
|  | *Meriania furvanthera* |  |  | 11 |  |  |  |
|  | *Meriania hexamera* |  |  | 16 |  |  |  |
|  | *Meriania maguirei* |  |  | 2 |  |  |  |
|  | *Meriania maxima* |  |  | 24 |  |  |  |
|  | *Meriania rigida* |  |  | 2 |  |  |  |
|  | *Meriania silverstonei* |  | 1 |  |  |  |  |
|  | *Meriania tomentosa* |  |  | 58 |  |  |  |
|  | *Miconia acanthocoryne* |  | 21 |  |  |  |  |
|  | *Miconia acuminata* |  |  | 3 |  |  |  |
|  | *Miconia affinis* |  | 2 |  |  | 4 |  |
|  | *Miconia alargada* |  |  | 1 |  |  |  |
|  | *Miconia amplexicaulis* |  |  | 4 |  |  |  |
|  | *Miconia aponeura* |  | 6 |  |  |  |  |
|  | *Miconia aristata* |  |  |  | 17 |  |  |
|  | *Miconia astroplocama* |  |  |  | 2 |  |  |
|  | *Miconia aulocalyx* |  |  |  | 1 |  |  |
|  | *Miconia aureoides* |  |  | 1 |  |  |  |
|  | *Miconia bangii* |  |  |  |  | 55 |  |
|  | *Miconia barbeyana* |  |  |  | 21 | 1 |  |
|  | *Miconia biacuta* |  |  |  |  | 43 |  |
|  | *Miconia biglandulosa* |  |  | 1 |  |  |  |
|  | *Miconia boliviensis* |  |  |  |  | 516 |  |
|  | *Miconia brachyanthera* |  |  |  | 14 |  |  |
|  | *Miconia brachycalyx* |  |  | 13 |  |  |  |
|  | *Miconia bracteata* |  |  | 1 |  |  |  |
|  | *Miconia bracteolata* |  |  | 59 | 71 |  |  |
|  | *Miconia brevistylis* |  |  |  | 39 |  |  |
|  | *Miconia brevitheca* |  |  | 14 |  |  |  |
|  | *Miconia brittonii* |  |  |  |  | 73 |  |
|  | *Miconia buxifolia* |  |  | 1 |  |  |  |
|  | *Miconia caelata* |  |  | 4 |  |  |  |
|  | *Miconia caerulea* |  |  |  | 3 |  |  |
|  | *Miconia cajanumana* |  |  | 6 |  |  |  |
|  | *Miconia calophylla* |  |  | 10 | 1 |  |  |
|  | *Miconia calvescens* |  |  | 12 |  |  |  |
|  | *Miconia caudata* |  | 2 |  |  |  |  |
|  | *Miconia centrodesma* |  |  |  |  | 12 |  |
|  | *Miconia cercophora* |  |  | 1 |  |  |  |
|  | *Miconia cladonia* |  |  | 3 |  |  |  |
|  | *Miconia clathrantha* |  |  | 190 |  |  |  |
|  | *Miconia cookii* |  |  |  | 17 |  |  |
|  | *Miconia cordata* |  |  |  |  | 3 |  |
|  | *Miconia corymbiformis* |  |  | 56 |  |  |  |
|  | *Miconia corymbosa* |  |  | 6 |  |  |  |
|  | *Miconia crassinervia* |  | 3 |  |  |  |  |
|  | *Miconia crassistigma* |  |  |  | 181 |  |  |
|  | *Miconia crebribullata* |  |  | 8 |  |  |  |
|  | *Miconia crocea* |  |  | 19 |  |  |  |
|  | *Miconia cyanocarpa* |  |  |  |  | 1 |  |
|  | *Miconia dapsiliflora* |  |  | 3 |  |  |  |
|  | *Miconia decurrens* |  |  | 4 |  |  |  |
|  | *Miconia dodecandra* | 12 |  | 1 |  |  |  |
|  | *Miconia dolichopoda* |  | 18 |  |  |  |  |
|  | *Miconia elata* |  | 92 | 7 |  |  |  |
|  | *Miconia elongata* |  |  |  | 45 | 50 |  |
|  | *Miconia floribunda* |  | 4 | 4 |  |  |  |
|  | *Miconia glandulistyla* |  |  | 2 |  |  |  |
|  | *Miconia glaucescens* |  |  | 3 |  |  |  |
|  | *Miconia goniostigma* |  |  | 2 |  |  |  |
|  | *Miconia grandifolia* |  |  | 11 |  |  |  |
|  | *Miconia hygrophila* |  |  |  | 1 |  |  |
|  | *Miconia incachacana* |  |  |  |  | 7 |  |
|  | *Miconia insularis* |  |  | 4 |  |  |  |
|  | *Miconia jahnii* |  | 1 | 33 |  |  |  |
|  | *Miconia klugii* |  |  | 1 |  |  |  |
|  | *Miconia lamprophylla* |  |  |  | 1 |  |  |
|  | *Miconia longifolia* |  | 3 |  |  |  |  |
|  | *Miconia loreyoides* |  |  | 8 |  |  |  |
|  | *Miconia loxensis* |  |  | 7 |  |  |  |
|  | *Miconia lutescens* |  |  | 2 |  |  |  |
|  | *Miconia madisonii* |  |  |  | 50 |  |  |
|  | *Miconia mandonii* |  |  |  |  | 62 |  |
|  | *Miconia media* |  |  | 1 |  |  |  |
|  | *Miconia micropetala* |  | 7 | 7 |  |  |  |
|  | *Miconia minutiflora* | 3 | 7 | 1 |  |  |  |
|  | *Miconia mirabilis* |  | 8 |  |  |  |  |
|  | *Miconia molybdea* |  |  |  |  |  | 660 |
|  | *Miconia multispicata* |  |  | 1 |  | 11 |  |
|  | *Miconia napoana* |  |  | 7 |  |  |  |
|  | *Miconia nodosa* |  |  | 2 |  |  |  |
|  | *Miconia nutans* |  |  | 3 |  |  |  |
|  | *Miconia ochracea* |  |  | 58 |  |  |  |
|  | *Miconia ostrigio* |  |  | 1 |  |  |  |
|  | *Miconia paleacea* |  |  | 1 |  |  |  |
|  | *Miconia pilgeriana* |  |  | 4 |  | 9 |  |
|  | *Miconia pilosa* |  |  | 2 |  |  |  |
|  | *Miconia poeppigii* |  | 37 |  |  |  |  |
|  | *Miconia poortmannii* |  |  | 49 |  |  |  |
|  | *Miconia prasina* |  | 1 | 1 |  |  |  |
|  | *Miconia punctata* |  |  | 83 |  | 1 |  |
|  | *Miconia quadrialata* |  |  |  |  | 3 |  |
|  | *Miconia quadripora* |  |  | 1 |  |  |  |
|  | *Miconia rivetii* |  |  | 3 |  |  |  |
|  | *Miconia rubescens* |  |  | 1 |  |  |  |
|  | *Miconia setulosa* |  |  |  | 71 | 3 |  |
|  | *Miconia spennerostachya* |  |  |  | 5 |  |  |
|  | *Miconia spicellata* |  | 4 |  |  |  |  |
|  | *Miconia splendens* |  |  |  |  | 1 |  |
|  | *Miconia stelligera* |  |  |  | 1 |  |  |
|  | *Miconia terborghii* |  |  |  | 1 |  |  |
|  | *Miconia theaezans* |  | 23 | 193 | 4 | 68 |  |
|  | *Miconia tinifolia* | 1 |  | 35 |  |  |  |
|  | *Miconia tonduzii* |  | 7 |  |  |  |  |
|  | *Miconia transversa* |  |  | 1 |  |  |  |
|  | *Miconia trinervia* |  | 25 | 4 |  |  |  |
|  | *Miconia triplinervis* |  |  | 1 |  |  |  |
|  | *Miconia villonacensis* |  |  | 7 |  |  |  |
|  | *Miconia wurdackii* |  | 1 |  |  |  |  |
|  | *Miconia zubenetana* |  |  | 1 |  |  |  |
|  | *Miconia meridensis* | 1 |  |  |  |  |  |
|  | *Miconia mesmeana* | 1 |  |  |  |  |  |
|  | *Miconia tabayensis* | 2 |  |  |  |  |  |
|  | *Mouriri colombiana* |  | 2 |  |  |  |  |
|  | *Mouriri completens* |  | 8 |  |  |  |  |
|  | *Mouriri laxiflora* |  |  | 1 |  |  |  |
|  | *Mouriri nigra* |  | 1 |  |  |  |  |
|  | *Ossaea laxivenula* |  |  | 1 |  |  |  |
|  | *Tessmannianthus heterostemon* |  |  | 12 |  |  |  |
|  | *Tibouchina bicolor* |  |  |  |  | 2 |  |
|  | *Tibouchina calycina* |  |  |  |  | 9 |  |
|  | *Tibouchina dimorphophylla* |  |  |  | 4 |  |  |
|  | *Tibouchina lepidota* |  | 38 | 2 |  |  |  |
|  | *Tibouchina ochypetala* |  |  | 2 |  |  |  |
| Meliaceae | *Cabralea canjerana* |  |  | 2 |  | 30 |  |
|  | *Carapa guianensis* |  |  | 2 |  |  |  |
|  | *Carapa nicaraguensis* |  |  | 2 |  |  |  |
|  | *Cedrela angustifolia* |  |  |  |  |  | 336 |
|  | *Cedrela balansae* |  |  |  |  |  | 192 |
|  | *Cedrela fissilis* |  |  |  |  | 19 |  |
|  | *Cedrela montana* |  |  | 15 | 2 |  |  |
|  | *Cedrela nebulosa* |  |  | 2 |  |  |  |
|  | *Cedrela odorata* |  | 1 | 9 |  |  |  |
|  | *Guarea cartaguenya* |  | 2 | 2 |  |  |  |
|  | *Guarea glabra* |  | 6 |  |  |  |  |
|  | *Guarea gomma* |  |  |  |  | 1 |  |
|  | *Guarea guidonia* |  | 4 | 1 |  | 1 |  |
|  | *Guarea kunthiana* |  | 7 | 167 | 13 |  |  |
|  | *Guarea macrophylla* |  | 18 | 14 |  |  |  |
|  | *Guarea persistens* |  |  | 1 |  |  |  |
|  | *Guarea pterorhachis* |  | 1 | 48 |  |  |  |
|  | *Guarea pubescens* |  |  | 2 |  |  |  |
|  | *Guarea purusana* |  |  | 3 |  |  |  |
|  | *Guarea silvatica* |  |  | 4 |  |  |  |
|  | *Guarea subandina* |  |  | 24 |  |  |  |
|  | *Ruagea glabra* |  | 8 | 13 | 3 | 112 |  |
|  | *Ruagea hirsuta* |  |  | 15 | 18 |  |  |
|  | *Ruagea microphylla* |  |  | 1 |  |  |  |
|  | *Ruagea ovalis* |  |  | 3 |  | 13 |  |
|  | *Ruagea pubescens* | 6 | 6 | 9 |  |  |  |
|  | *Ruagea subviridiflora* |  |  |  | 5 |  |  |
|  | *Ruagea tomentosa* |  |  | 6 |  |  |  |
|  | *Toona ciliata* |  |  |  |  |  | 8 |
|  | *Trichilia catigua* |  |  |  |  | 387 |  |
|  | *Trichilia cipo* |  |  | 5 |  |  |  |
|  | *Trichilia claussenii* |  |  |  |  | 24 | 434 |
|  | *Trichilia elegans* |  |  |  |  | 127 |  |
|  | *Trichilia elsae* |  |  | 1 |  |  |  |
|  | *Trichilia hirta* |  |  | 2 |  |  |  |
|  | *Trichilia hispida* |  |  | 1 |  |  |  |
|  | *Trichilia laxipaniculata* |  |  | 2 |  |  |  |
|  | *Trichilia martiana* |  | 1 | 3 |  |  |  |
|  | *Trichilia maynasiana* |  |  | 7 |  |  |  |
|  | *Trichilia micrantha* |  |  | 1 |  |  |  |
|  | *Trichilia pallida* |  | 3 | 18 |  | 5 |  |
|  | *Trichilia pleeana* |  | 7 | 1 |  | 103 |  |
|  | *Trichilia poeppigii* |  | 3 | 1 |  |  |  |
|  | *Trichilia primogenita* |  |  | 2 |  |  |  |
|  | *Trichilia quadrijuga* |  | 10 |  |  |  |  |
|  | *Trichilia rubra* |  |  | 2 |  |  |  |
|  | *Trichilia septentrionalis* |  | 11 | 16 |  |  |  |
| Menispermaceae | *Anomospermum chloranthum* |  |  |  |  | 1 |  |
|  | *Curarea cuatrecasasii* |  | 7 |  |  |  |  |
| Metteniusaceae | *Metteniusa tessmanniana* |  |  | 28 |  |  |  |
| Monimiaceae | *Mollinedia lanceolata* |  |  |  | 13 | 7 |  |
|  | *Mollinedia minutiflora* |  | 1 |  |  |  |  |
|  | *Mollinedia ovata* |  |  | 4 | 15 | 72 |  |
|  | *Mollinedia repanda* |  |  | 5 | 7 | 39 |  |
|  | *Mollinedia simulans* |  |  |  | 1 |  |  |
|  | *Mollinedia tomentosa* |  |  | 2 |  |  |  |
| Moraceae | *Batocarpus costaricensis* |  | 10 | 1 |  |  |  |
|  | *Batocarpus orinocensis* |  |  | 53 |  |  |  |
|  | *Brosimum alicastrum* |  | 14 |  |  |  |  |
|  | *Brosimum guianense* |  | 18 | 2 |  |  |  |
|  | *Brosimum lactescens* |  |  | 7 | 2 | 45 |  |
|  | *Brosimum rubescens* |  | 19 |  |  |  |  |
|  | *Brosimum utile* |  | 5 | 18 |  |  |  |
|  | *Castilla elastica* |  |  | 15 |  |  |  |
|  | *Castilla tunu* |  | 37 |  |  |  |  |
|  | *Castilla ulei* |  |  | 4 |  |  |  |
|  | *Chlorophora tinctoria* |  |  |  |  |  | 8 |
|  | *Clarisia biflora* |  | 115 | 33 | 2 | 57 |  |
|  | *Clarisia racemosa* |  | 7 | 54 |  | 114 |  |
|  | *Dorstenia brasiliensis* |  |  |  |  | 3 |  |
|  | *Ficus acuminata* |  |  | 9 |  |  |  |
|  | *Ficus americana* |  | 8 | 9 | 16 | 13 |  |
|  | *Ficus apollinaris* |  |  | 1 |  |  |  |
|  | *Ficus banosensis* |  |  | 1 |  |  |  |
|  | *Ficus brevibracteata* |  |  | 6 |  |  |  |
|  | *Ficus bullenei* |  | 1 |  |  |  |  |
|  | *Ficus caballina* |  |  |  |  | 1 |  |
|  | *Ficus caldasiana* |  |  | 1 |  |  |  |
|  | *Ficus catrecasana* |  |  |  | 1 |  |  |
|  | *Ficus cervantesiana* |  |  | 40 |  |  |  |
|  | *Ficus citrifolia* |  |  | 4 |  | 4 |  |
|  | *Ficus crassiuscula* |  | 1 | 1 | 1 |  |  |
|  | *Ficus cuatrecasasiana* |  |  | 32 | 1 | 7 |  |
|  | *Ficus donnell-smithii* |  | 1 |  |  |  |  |
|  | *Ficus dulciaria* |  |  | 12 |  |  |  |
|  | *Ficus ecuadorensis* |  |  | 2 |  |  |  |
|  | *Ficus eximia* |  |  |  | 5 |  |  |
|  | *Ficus insipida* |  | 5 | 25 |  |  |  |
|  | *Ficus loxensis* |  |  | 1 |  |  |  |
|  | *Ficus macbridei* |  |  | 3 | 7 |  |  |
|  | *Ficus macrophylla* |  |  | 1 |  |  |  |
|  | *Ficus maroma* |  |  |  |  | 10 | 47 |
|  | *Ficus maxima* |  | 1 | 14 | 8 |  |  |
|  | *Ficus mutisii* |  | 17 | 1 | 7 |  |  |
|  | *Ficus nymphaeifolia* |  |  | 1 |  |  |  |
|  | *Ficus obtusifolia* |  | 1 |  |  | 4 |  |
|  | *Ficus pertusa* |  |  | 7 |  |  |  |
|  | *Ficus piresiana* |  |  | 3 |  |  |  |
|  | *Ficus popenoei* |  | 1 |  |  |  |  |
|  | *Ficus quijosana* |  |  | 12 |  |  |  |
|  | *Ficus schippii* |  | 1 | 2 |  |  |  |
|  | *Ficus tonduzii* |  |  | 11 |  |  |  |
|  | *Ficus tovarensis* |  | 2 |  |  |  |  |
|  | *Ficus trianae* |  |  | 2 |  |  |  |
|  | *Ficus trigona* |  |  | 2 | 1 | 6 |  |
|  | *Ficus vittata* |  |  | 4 |  |  |  |
|  | *Helianthostylis sprucei* |  | 11 |  |  |  |  |
|  | *Helicostylis scabra* |  |  | 3 |  |  |  |
|  | *Helicostylis tomentosa* |  | 33 | 24 | 3 | 39 |  |
|  | *Helicostylis tovarensis* |  | 3 | 21 |  | 64 |  |
|  | *Maclura tinctoria* |  | 1 | 6 |  | 2 | 39 |
|  | *Maquira calophylla* |  |  | 4 |  |  |  |
|  | *Maquira guianensis* |  | 55 |  | 2 |  |  |
|  | *Morus alba* |  |  |  |  |  | 272 |
|  | *Morus celtidifolia* |  |  | 1 |  |  |  |
|  | *Morus insignis* |  | 9 | 49 | 38 |  | 4 |
|  | *Naucleopsis capirensis* |  |  | 4 |  |  |  |
|  | *Naucleopsis chiguila* |  |  | 6 |  |  |  |
|  | *Naucleopsis concinna* |  |  | 1 |  |  |  |
|  | *Naucleopsis francisci* |  |  | 8 |  |  |  |
|  | *Naucleopsis glabra* |  | 3 |  |  |  |  |
|  | *Naucleopsis naga* |  |  | 8 |  |  |  |
|  | *Naucleopsis ulei* |  | 1 | 7 |  |  |  |
|  | *Perebea angustifolia* |  |  | 3 |  |  |  |
|  | *Perebea guianensis* |  | 2 | 20 | 3 | 82 |  |
|  | *Perebea humilis* |  |  |  | 1 |  |  |
|  | *Perebea xanthochyma* |  |  | 27 |  |  |  |
|  | *Poulsenia armata* |  |  | 6 | 4 |  |  |
|  | *Pseudolmedia gentryi* |  |  | 2 |  |  |  |
|  | *Pseudolmedia laevigata* |  | 83 | 66 | 3 | 2 |  |
|  | *Pseudolmedia laevis* |  |  | 17 |  | 12 |  |
|  | *Pseudolmedia macrophylla* |  |  | 10 |  |  |  |
|  | *Pseudolmedia rigida* |  |  | 5 | 3 | 67 |  |
|  | *Sorocea guilleminiana* |  |  |  | 1 | 20 |  |
|  | *Sorocea muriculata* |  |  |  | 1 |  |  |
|  | *Sorocea pubivena* |  | 1 | 4 |  |  |  |
|  | *Sorocea steinbachii* |  |  | 35 |  |  |  |
|  | *Sorocea trophoides* |  | 2 | 106 |  |  |  |
|  | *Trophis caucana* |  |  | 5 |  |  |  |
|  | *Trophis mexicana* |  |  | 2 |  |  |  |
|  | *Trophis racemosa* |  | 6 |  |  |  |  |
| Muntingiaceae | *Muntingia calabura* |  |  |  |  |  | 5 |
| Myricaceae | *Morella parvifolia* |  |  | 23 |  |  |  |
|  | *Morella pubescens* |  |  | 12 | 29 | 58 |  |
| Myristicaceae | *Compsoneura capitellata* |  |  | 14 |  |  |  |
|  | *Compsoneura claroensis* |  | 5 |  |  |  |  |
|  | *Compsoneura mutisii* |  | 7 |  |  |  |  |
|  | *Iryanthera grandis* |  |  | 2 |  |  |  |
|  | *Iryanthera hostmannii* |  | 29 |  |  |  |  |
|  | *Iryanthera juruensis* |  |  | 6 |  |  |  |
|  | *Osteophloeum platyspermum* |  |  | 13 |  |  |  |
|  | *Otoba glycycarpa* |  |  | 62 |  |  |  |
|  | *Otoba gordoniifolia* |  |  | 35 |  |  |  |
|  | *Otoba novogranatensis* |  | 34 | 29 |  |  |  |
|  | *Otoba parvifolia* |  |  | 247 | 20 |  |  |
|  | *Virola calophylla* |  |  | 30 |  |  |  |
|  | *Virola dixonii* |  | 3 | 6 |  |  |  |
|  | *Virola duckei* |  |  | 15 |  |  |  |
|  | *Virola elongata* |  |  | 7 |  |  |  |
|  | *Virola flexuosa* |  | 44 | 16 |  |  |  |
|  | *Virola macrocarpa* |  | 55 | 1 |  |  |  |
|  | *Virola multinervia* |  |  | 5 |  |  |  |
|  | *Virola obovata* |  |  | 7 |  |  |  |
|  | *Virola pavonis* |  |  | 14 |  |  |  |
|  | *Virola peruviana* |  |  | 8 |  | 37 |  |
|  | *Virola reidii* |  |  | 1 |  |  |  |
|  | *Virola sebifera* |  | 148 | 16 |  | 5 |  |
| Myrtaceae | *Blepharocalyx salicifolius* |  |  |  |  | 1 | 934 |
|  | *Calyptranthes bipennis* |  |  | 2 |  |  |  |
|  | *Calyptranthes concinna* |  |  | 1 |  |  |  |
|  | *Calyptranthes densiflora* |  |  | 2 |  |  |  |
|  | *Calyptranthes longifolia* |  |  |  |  | 12 |  |
|  | *Calyptranthes nervata* |  |  | 2 |  |  |  |
|  | *Calyptranthes paniculata* |  |  | 4 |  |  |  |
|  | *Calyptranthes plicata* |  |  | 14 |  |  |  |
|  | *Calyptranthes pulchella* |  |  | 4 |  |  |  |
|  | *Calyptranthes simulata* |  |  |  |  | 1 |  |
|  | *Calyptranthes speciosa* |  | 1 |  |  |  |  |
|  | *Campomanesia lineatifolia* |  |  | 1 |  | 4 |  |
|  | *Eugenia biflora* |  | 5 |  |  |  |  |
|  | *Eugenia crassimarginata* |  |  | 9 |  |  |  |
|  | *Eugenia dittocrepis* |  | 7 |  |  |  |  |
|  | *Eugenia egensis* |  |  |  |  | 14 |  |
|  | *Eugenia excelsa* |  |  |  |  | 2 |  |
|  | *Eugenia feijoi* |  |  |  |  | 6 |  |
|  | *Eugenia flavescens* |  |  |  |  | 1 |  |
|  | *Eugenia florida* |  | 1 | 14 | 3 | 23 |  |
|  | *Eugenia hiemalis* |  |  |  |  |  | 6 |
|  | *Eugenia involucrata* |  |  |  |  | 1 |  |
|  | *Eugenia lambertiana* |  |  |  |  | 2 |  |
|  | *Eugenia ligustrina* |  |  |  |  | 1 |  |
|  | *Eugenia marlierioides* |  |  |  |  | 1 |  |
|  | *Eugenia marowynensis* |  |  | 3 |  |  |  |
|  | *Eugenia moraviana* |  |  |  |  | 2 | 5 |
|  | *Eugenia oerstediana* |  |  | 4 |  |  |  |
|  | *Eugenia patens* |  |  | 5 |  |  |  |
|  | *Eugenia pusilliflora* |  |  | 1 |  |  |  |
|  | *Eugenia repanda* |  |  |  |  |  | 1 |
|  | *Eugenia schunkei* |  |  | 6 |  |  |  |
|  | *Eugenia sericifolia* |  |  | 10 |  |  |  |
|  | *Eugenia speciosa* |  |  |  |  | 5 |  |
|  | *Eugenia tamaensis* | 51 |  |  |  |  |  |
|  | *Eugenia uniflora* |  |  |  |  | 26 | 522 |
|  | *Myrcia aliena* |  |  | 2 |  |  |  |
|  | *Myrcia atrorufa* |  |  |  | 4 |  |  |
|  | *Myrcia ayabambensis* |  |  | 4 |  |  |  |
|  | *Myrcia barituensis* |  |  |  |  |  | 122 |
|  | *Myrcia coumete* |  | 8 |  |  |  |  |
|  | *Myrcia fallax* |  | 28 | 18 | 60 | 41 |  |
|  | *Myrcia fenzliana* |  |  |  |  | 24 |  |
|  | *Myrcia florida* |  | 4 |  |  |  |  |
|  | *Myrcia mollis* |  |  |  | 1 | 3 |  |
|  | *Myrcia obumbrans* |  |  | 2 |  |  |  |
|  | *Myrcia paivae* |  | 4 |  |  |  |  |
|  | *Myrcia popayanensis* |  | 13 |  |  |  |  |
|  | *Myrcia rostrata* |  |  |  | 114 |  |  |
|  | *Myrcia sellowiana* |  | 4 |  |  |  |  |
|  | *Myrcia splendens* | 67 | 4 | 35 |  |  |  |
|  | *Myrcia sylvatica* |  |  |  |  | 2 |  |
|  | *Myrcianthes alaternifolia* |  |  | 1 |  |  |  |
|  | *Myrcianthes callicoma* |  |  |  |  |  | 18 |
|  | *Myrcianthes discolor* |  |  | 9 |  |  |  |
|  | *Myrcianthes fragrans* |  |  | 1 |  |  |  |
|  | *Myrcianthes hallii* |  |  | 11 |  |  |  |
|  | *Myrcianthes mato* |  |  |  |  | 87 | 314 |
|  | *Myrcianthes myrsinoides* |  |  | 1 |  |  |  |
|  | *Myrcianthes orthostemon* |  |  | 9 |  |  |  |
|  | *Myrcianthes pseudomato* |  |  |  |  |  | 268 |
|  | *Myrcianthes pungens* |  |  |  |  |  | 737 |
|  | *Myrcianthes rhopaloides* |  | 3 | 35 | 86 |  |  |
|  | *Myrciaria floribunda* |  | 2 |  |  | 7 | 48 |
|  | *Psidium guajava* |  |  |  |  |  | 3 |
|  | *Siphoneugena densiflora* |  |  | 7 | 2 |  | 282 |
|  | *Siphoneugena occidentalis* |  |  |  |  | 68 |  |
|  | *Syzygium aemulum* |  |  |  | 19 |  |  |
|  | *Syzygium brachycalyx* |  |  | 1 |  |  |  |
| Nyctaginaceae | *Bougainvillea modesta* |  |  |  |  | 7 |  |
|  | *Bougainvillea stipitata* |  |  |  |  | 3 | 55 |
|  | *Guapira costaricana* |  | 48 |  |  |  |  |
|  | *Guapira noxia* |  |  |  | 6 |  |  |
|  | *Guapira opposita* |  |  | 1 |  | 15 |  |
|  | *Neea amplifolia* |  | 16 |  |  |  |  |
|  | *Neea divaricata* |  | 1 | 8 |  |  |  |
|  | *Neea hermaphrodita* |  |  | 1 |  | 26 |  |
|  | *Neea macrophylla* |  |  | 2 |  |  |  |
|  | *Neea ovalifolia* |  |  | 16 |  | 3 |  |
|  | *Neea parviflora* |  | 1 |  |  |  |  |
|  | *Pisonia zapallo* |  |  |  |  | 5 | 512 |
| Ochnaceae | *Cespedesia spathulata* |  |  | 1 |  |  |  |
|  | *Godoya antioquiensis* |  | 11 |  |  |  |  |
|  | *Lacunaria crenata* |  |  | 1 |  |  |  |
|  | *Lacunaria jenmanii* |  | 1 |  |  |  |  |
|  | *Ouratea castaneifolia* |  | 1 |  |  |  |  |
|  | *Ouratea rubricyanea* |  | 2 |  |  |  |  |
|  | *Perissocarpa steyermarkii* |  | 9 |  |  |  |  |
|  | *Quiina florida* |  |  |  |  | 11 |  |
|  | *Quiina pteridophylla* |  | 6 |  |  |  |  |
|  | *Quiina zamorensis* |  |  | 1 |  |  |  |
| Olacaceae | *Aptandra tubicina* |  | 2 |  |  |  |  |
|  | *Cathedra acuminata* |  |  | 3 |  |  |  |
|  | *Heisteria acuminata* |  | 17 | 6 | 51 |  |  |
|  | *Heisteria asplundii* |  |  | 14 | 35 |  |  |
|  | *Heisteria latifolia* |  | 1 |  |  |  |  |
|  | *Heisteria nitida* |  |  | 4 |  |  |  |
|  | *Heisteria pacifica* |  |  | 1 |  |  |  |
|  | *Heisteria spruceana* |  |  | 2 |  |  |  |
|  | *Minquartia guianensis* |  | 1 | 7 |  |  |  |
| Oleaceae | *Ligustrum lucidum* |  |  |  |  |  | 47 |
| Opiliaceae | *Agonandra excelsa* |  |  |  |  | 37 | 130 |
|  | *Agonandra peruviana* |  |  |  |  | 15 |  |
| Passifloraceae | *Passiflora macrophylla* |  |  | 2 |  |  |  |
| Pentaphylacaceae | *Freziera angulosa* |  |  |  | 1 |  |  |
|  | *Freziera arbutifolia* |  | 2 |  |  |  |  |
|  | *Freziera caesariata* |  |  |  |  | 26 |  |
|  | *Freziera canescens* |  |  | 22 |  |  |  |
|  | *Freziera chrysophylla* |  | 1 |  |  |  |  |
|  | *Freziera dudleyi* |  |  |  | 3 | 50 |  |
|  | *Freziera karsteniana* |  |  | 1 | 29 |  |  |
|  | *Freziera lanata* |  |  |  | 8 | 3 |  |
|  | *Freziera punctata* |  | 3 |  |  |  |  |
|  | *Freziera reticulata* |  |  | 3 |  |  |  |
|  | *Freziera verrucosa* |  | 9 | 30 |  |  |  |
|  | *Ternstroemia asymmetrica* |  |  |  |  | 3 |  |
|  | *Ternstroemia brachypoda* |  |  |  | 11 |  |  |
|  | *Ternstroemia cleistogama* |  |  | 5 |  |  |  |
|  | *Ternstroemia jelskii* |  |  | 1 |  |  |  |
|  | *Ternstroemia killipeana* |  |  |  | 8 |  |  |
|  | *Ternstroemia lehmannii* |  |  | 1 |  |  |  |
|  | *Ternstroemia luquillensis* |  |  |  | 66 |  |  |
|  | *Ternstroemia macrocarpa* |  | 10 | 7 |  |  |  |
|  | *Ternstroemia subserrata* |  |  |  |  | 64 |  |
|  | *Ternstroemia acrodantha* | 8 |  |  |  |  |  |
| Peraceae | *Chaetocarpus myrsinites* |  |  |  |  | 4 |  |
| Petiveriaceae | *Gallesia integrifolia* |  |  |  |  | 127 |  |
| Phyllanthaceae | *Hieronyma alchorneoides* |  | 3 | 18 | 3 |  |  |
|  | *Hieronyma antioquensis* |  | 93 |  |  |  |  |
|  | *Hieronyma asperifolia* |  |  | 29 |  |  |  |
|  | *Hieronyma duquei* |  | 64 | 39 | 1 |  |  |
|  | *Hieronyma fendleri* |  |  | 39 | 5 | 175 |  |
|  | *Hieronyma macrocarpa* |  |  | 57 |  |  |  |
|  | *Hieronyma oblonga* | 19 | 16 | 47 | 8 | 1 |  |
|  | *Hieronyma scabrida* |  | 2 | 2 |  |  |  |
|  | *Margaritaria nobilis* |  |  | 2 |  | 1 |  |
|  | *Phyllanthus acuminatus* |  |  |  |  |  | 6 |
|  | *Phyllanthus attenuatus* |  | 1 |  |  |  |  |
|  | *Phyllanthus callejasii* |  | 1 |  |  |  |  |
|  | *Richeria grandis* |  | 20 | 4 |  | 31 |  |
| Phytolaccaceae | *Seguieria aculeata* |  |  |  |  | 3 |  |
| Picramniaceae | *Picramnia caracasana* |  |  | 2 |  |  |  |
|  | *Picramnia latifolia* |  | 1 | 2 |  |  |  |
|  | *Picramnia sellowii* |  |  | 3 |  |  |  |
| Picrodendraceae | *Parodiodendron marginivillosum* |  |  |  |  |  | 1 |
| Piperaceae | *Piper kelleyi* |  |  | 2 |  |  |  |
|  | *Piper aduncum* |  |  | 3 |  |  |  |
|  | *Piper andreanum* |  |  | 11 |  |  |  |
|  | *Piper angamarcanum* |  |  | 6 |  |  |  |
|  | *Piper arboreum* |  |  | 5 |  | 2 |  |
|  | *Piper barbatum* |  |  | 1 |  |  |  |
|  | *Piper begoniicolor* |  | 2 |  |  |  |  |
|  | *Piper bolivianum* |  |  |  |  | 3 |  |
|  | *Piper calophyllum* |  |  |  | 1 |  |  |
|  | *Piper carpunya* |  |  | 10 |  |  |  |
|  | *Piper coruscans* |  |  |  | 1 |  |  |
|  | *Piper crassinervium* |  |  |  | 1 |  |  |
|  | *Piper dasyoura* |  |  |  | 23 |  |  |
|  | *Piper ecuadorense* |  |  | 4 |  |  |  |
|  | *Piper imperiale* |  | 27 |  |  |  |  |
|  | *Piper lacunosum* |  |  | 1 |  |  |  |
|  | *Piper longifolium* |  |  | 1 |  |  |  |
|  | *Piper longispicum* | 5 |  |  |  |  |  |
|  | *Piper obliquum* |  | 21 | 5 |  |  |  |
|  | *Piper obtusilimbum* |  |  | 2 |  |  |  |
|  | *Piper pareolatum* |  |  |  | 6 |  |  |
|  | *Piper peltatum* |  |  | 1 |  |  |  |
|  | *Piper reticulatum* |  | 4 |  |  |  |  |
|  | *Piper tucumanum* |  |  |  |  | 60 | 732 |
|  | *Piper umbellatum* |  |  | 1 |  |  |  |
| Poaceae | *Pinga marginata* |  | 1 |  |  |  |  |
| Podocarpaceae | *Podocarpus ingensis* |  |  | 11 |  | 7 |  |
|  | *Podocarpus macrostachys* |  |  | 6 |  |  |  |
|  | *Podocarpus oleifolius* |  | 14 | 37 | 283 | 178 |  |
|  | *Podocarpus parlatorei* |  |  |  |  |  | 2797 |
|  | *Prumnopitys harmsiana* |  |  | 22 |  |  |  |
|  | *Prumnopitys montana* |  |  | 11 | 12 |  |  |
|  | *Retrophyllum rospigliosii* |  |  | 28 | 5 |  |  |
| Polygalaceae | *Monnina connectisepala* |  |  |  | 1 |  |  |
|  | *Monnina pseudopilosa* |  |  | 2 |  |  |  |
|  | *Monnina pulchra* |  |  | 1 |  |  |  |
|  | *Moutabea aculeata* |  |  | 1 |  |  |  |
|  | *Moutabea gentryi* |  | 2 |  |  |  |  |
| Polygonaceae | *Coccoloba acuminata* |  | 15 |  |  |  |  |
|  | *Coccoloba ascendens* |  | 2 |  |  |  |  |
|  | *Coccoloba cordata* |  |  |  |  |  | 7 |
|  | *Coccoloba coronata* |  | 2 |  |  |  |  |
|  | *Coccoloba densifrons* |  | 5 | 7 |  |  |  |
|  | *Coccoloba excelsa* |  | 5 |  |  |  |  |
|  | *Coccoloba mollis* |  | 2 |  |  | 12 |  |
|  | *Coccoloba obovata* |  |  | 4 |  |  |  |
|  | *Coccoloba padiformis* |  | 25 |  |  |  |  |
|  | *Coccoloba peruviana* |  |  |  |  | 14 |  |
|  | *Coccoloba ruiziana* |  |  | 5 |  |  |  |
|  | *Coccoloba tiliacea* |  |  |  |  |  | 79 |
|  | *Ruprechtia apetala* |  |  |  |  | 45 | 530 |
|  | *Ruprechtia laxiflora* |  |  |  |  | 1 | 194 |
|  | *Triplaris americana* |  | 15 | 1 | 2 | 7 |  |
|  | *Triplaris cumingiana* |  |  | 26 |  |  |  |
|  | *Triplaris efistulifera* |  |  |  |  | 27 |  |
|  | *Triplaris vestita* |  |  |  |  | 6 |  |
| Primulaceae | *Ardisia ambigua* |  |  | 7 |  |  |  |
|  | *Ardisia foetida* |  | 4 |  |  |  |  |
|  | *Ardisia guianensis* |  |  | 1 |  |  |  |
|  | *Clavija laplanadae* |  |  | 2 |  |  |  |
|  | *Clavija longifolia* |  |  |  | 1 |  |  |
|  | *Clavija nutans* |  |  |  |  | 5 |  |
|  | *Cybianthus cuatrecasasii* |  |  | 2 |  |  |  |
|  | *Cybianthus occigranatensis* |  |  | 4 |  |  |  |
|  | *Geissanthus ambigua* |  |  | 16 |  | 16 |  |
|  | *Geissanthus andinus* |  |  | 15 |  |  |  |
|  | *Geissanthus bangii* |  |  |  |  | 1 |  |
|  | *Geissanthus ecuadorensis* |  |  | 28 |  |  |  |
|  | *Geissanthus longistamineus* |  |  | 2 |  |  |  |
|  | *Geissanthus occidentalis* |  | 6 |  |  |  |  |
|  | *Geissanthus pichinchae* |  |  | 2 |  |  |  |
|  | *Geissanthus vanderwerffii* |  |  | 15 | 1 |  |  |
|  | *Geissanthus floribundus* | 7 |  |  |  |  |  |
|  | *Myrsine andina* |  |  | 49 | 319 |  |  |
|  | *Myrsine coriacea* | 17 | 38 | 28 | 149 | 384 | 2 |
|  | *Myrsine dependens* |  |  | 28 | 5 | 3 |  |
|  | *Myrsine guianensis* |  | 3 |  |  |  |  |
|  | *Myrsine laetevirens* |  |  |  |  |  | 254 |
|  | *Myrsine latifolia* |  |  |  |  | 3 |  |
|  | *Myrsine manglilla* |  |  | 3 |  |  |  |
|  | *Myrsine pearcei* |  |  |  |  | 36 |  |
|  | *Myrsine pellucida* |  |  |  | 9 | 69 |  |
|  | *Myrsine pellucidopunctata* |  | 26 |  |  |  |  |
|  | *Myrsine sodiroana* |  |  | 20 |  |  |  |
|  | *Myrsine youngii* |  |  |  | 2 |  |  |
|  | *Stylogyne longifolia* |  |  | 3 |  |  |  |
|  | *Stylogyne micrantha* |  |  | 8 |  |  |  |
| Proteaceae | *Euplassa duquei* |  | 1 |  |  |  |  |
|  | *Euplassa occidentalis* |  |  | 3 |  |  |  |
|  | *Lomatia hirsuta* |  |  | 2 |  |  |  |
|  | *Oreocallis grandiflora* |  |  | 31 | 45 |  |  |
|  | *Panopsis megistosperma* |  |  | 1 |  |  |  |
|  | *Panopsis pearcei* |  |  |  | 9 | 9 |  |
|  | *Panopsis polystachya* |  | 12 |  |  |  |  |
|  | *Panopsis yolombo* |  | 17 |  |  |  |  |
|  | *Roupala cordifolia* |  |  |  |  | 4 |  |
|  | *Roupala loxensis* |  |  | 1 |  |  |  |
|  | *Roupala monosperma* |  |  |  | 17 | 1 |  |
|  | *Roupala montana* |  | 6 | 15 |  |  | 133 |
|  | *Roupala pachypoda* |  |  |  | 15 |  |  |
| Putranjivaceae | *Drypetes amazonica* |  |  | 2 | 4 |  |  |
|  | *Drypetes variabilis* |  |  | 2 |  |  |  |
| Rhamnaceae | *Condalia buxifolia* |  |  |  |  |  | 2 |
|  | *Frangula acuminata* |  |  |  | 3 |  |  |
|  | *Frangula granulosa* |  | 13 | 1 |  |  |  |
|  | *Frangula sphaerosperma* |  | 2 |  |  | 1 | 9 |
|  | *Scutia buxifolia* |  |  |  |  |  | 42 |
| Rhizophoraceae | *Cassipourea guianensis* |  | 9 |  |  |  |  |
|  | *Sterigmapetalum colombianum* |  | 1 |  |  |  |  |
| Rosaceae | *Hesperomeles ferruginea* |  |  | 48 | 43 | 61 |  |
|  | *Hesperomeles obtusifolia* |  |  | 3 | 2 |  |  |
|  | *Polylepis pauta* |  |  | 170 |  |  |  |
|  | *Prunus amplifolia* |  |  | 1 |  | 1 |  |
|  | *Prunus brittoniana* |  |  |  |  | 51 |  |
|  | *Prunus debilis* |  |  | 4 |  |  |  |
|  | *Prunus guanaiensis* |  |  | 8 |  |  |  |
|  | *Prunus herthae* |  |  | 20 | 14 |  |  |
|  | *Prunus huantensis* |  |  | 40 | 77 |  |  |
|  | *Prunus integrifolia* |  | 3 |  | 121 | 259 |  |
|  | *Prunus littlei* |  |  | 1 |  |  |  |
|  | *Prunus muris* |  | 1 |  |  |  |  |
|  | *Prunus ocellata* |  | 1 |  |  |  |  |
|  | *Prunus opaca* |  | 2 | 16 | 34 |  |  |
|  | *Prunus pearcei* |  |  |  |  | 1 |  |
|  | *Prunus persica* |  |  |  |  |  | 16 |
|  | *Prunus pleiantha* |  |  |  | 8 |  |  |
|  | *Prunus rigida* |  |  |  | 28 |  |  |
|  | *Prunus stipulata* |  |  |  | 1 | 10 |  |
|  | *Prunus subcorymbosa* |  | 7 | 3 |  | 2 |  |
|  | *Prunus tucumanensis* |  |  |  |  |  | 720 |
|  | *Prunus moritziana* | 15 |  |  |  |  |  |
| Rubiaceae | *Agouticarpa hirsuta* |  |  | 1 |  |  |  |
|  | *Agouticarpa williamsii* |  | 3 |  |  |  |  |
|  | *Alibertia acuminata* |  |  | 3 |  | 1 |  |
|  | *Alibertia patinoi* |  |  | 2 |  |  |  |
|  | *Alseis blackiana* |  | 2 |  |  |  |  |
|  | *Amaioua corymbosa* |  | 15 |  |  |  |  |
|  | *Amaioua guianensis* |  | 1 |  |  |  |  |
|  | *Arachnothryx lojensis* |  |  | 2 |  |  |  |
|  | *Calycophyllum candidissimum* |  | 1 |  |  |  |  |
|  | *Calycophyllum multiflorum* |  |  |  |  |  | 364 |
|  | *Capirona decorticans* |  |  | 5 |  |  |  |
|  | *Chimarrhis hookeri* |  |  | 1 |  |  |  |
|  | *Chomelia paniculata* |  |  | 4 |  |  |  |
|  | *Chomelia tenuiflora* |  |  | 3 |  |  |  |
|  | *Ciliosemina pedunculata* |  |  | 8 |  |  |  |
|  | *Cinchona antioquiae* |  | 10 |  |  |  |  |
|  | *Cinchona calisaya* |  |  |  | 49 | 52 |  |
|  | *Cinchona krauseana* |  |  | 1 |  |  |  |
|  | *Cinchona macrocalyx* |  |  |  | 15 |  |  |
|  | *Cinchona officinalis* |  | 5 |  | 3 |  |  |
|  | *Cinchona pubescens* | 38 | 1 | 16 | 30 | 1 |  |
|  | *Condaminea corymbosa* |  |  | 9 |  |  |  |
|  | *Cordiera longicaudata* |  |  | 1 |  |  |  |
|  | *Cosmibuena grandiflora* |  | 2 |  |  |  |  |
|  | *Coussarea antioquiana* |  | 1 |  |  |  |  |
|  | *Coussarea boliviensis* |  |  |  |  | 3 |  |
|  | *Coussarea brevicaulis* |  |  | 4 |  |  |  |
|  | *Coussarea cephaeloides* |  |  | 4 |  |  |  |
|  | *Coussarea ecuadorensis* |  |  | 3 |  |  |  |
|  | *Coussarea grandifolia* |  | 47 |  |  |  |  |
|  | *Coussarea latifolia* |  |  | 14 |  |  |  |
|  | *Coussarea paniculata* |  | 5 | 1 |  |  |  |
|  | *Coussarea racemosa* |  |  | 6 |  |  |  |
|  | *Coutarea hexandra* |  | 1 |  |  | 6 | 36 |
|  | *Dioicodendron dioicum* |  | 4 | 2 | 4 |  |  |
|  | *Duroia hirsuta* |  |  |  | 3 |  |  |
|  | *Duroia laevis* |  |  | 2 |  |  |  |
|  | *Elaeagia arborea* |  |  | 5 |  |  |  |
|  | *Elaeagia coriacea* |  |  | 1 |  |  |  |
|  | *Elaeagia ecuadorensis* |  |  | 8 |  |  |  |
|  | *Elaeagia karstenii* |  | 44 | 20 |  |  |  |
|  | *Elaeagia mariae* |  |  | 7 | 33 | 195 |  |
|  | *Elaeagia mollis* |  |  | 5 |  | 8 |  |
|  | *Elaeagia myriantha* |  | 3 | 5 |  |  |  |
|  | *Elaeagia pastoensis* |  | 50 |  |  |  |  |
|  | *Elaeagia utilis* |  | 8 | 35 |  |  |  |
|  | *Faramea bangii* |  |  | 2 | 5 | 1 |  |
|  | *Faramea candelabrum* |  |  |  | 28 | 8 |  |
|  | *Faramea coerulescens* |  |  | 2 |  |  |  |
|  | *Faramea eurycarpa* |  |  | 18 |  |  |  |
|  | *Faramea exemplaris* |  |  | 1 |  |  |  |
|  | *Faramea flavicans* | 10 |  | 6 |  |  |  |
|  | *Faramea glandulosa* |  |  | 3 |  |  |  |
|  | *Faramea langlassei* |  |  | 5 |  |  |  |
|  | *Faramea miconioides* |  |  | 4 |  |  |  |
|  | *Faramea oblongifolia* |  |  | 15 |  |  |  |
|  | *Faramea occidentalis* |  | 2 | 13 |  |  |  |
|  | *Faramea parvibractea* |  | 3 | 6 |  |  |  |
|  | *Faramea phyllonomoides* |  |  | 3 |  |  |  |
|  | *Faramea quinqueflora* |  |  | 1 |  |  |  |
|  | *Faramea sessiliflora* |  | 2 |  |  |  |  |
|  | *Faramea torquata* |  |  | 3 |  |  |  |
|  | *Faramea uncinata* |  |  | 2 |  |  |  |
|  | *Ferdinandusa chlorantha* |  |  | 17 |  |  |  |
|  | *Genipa americana* |  | 17 |  |  |  |  |
|  | *Guettarda aromatica* |  |  | 1 |  |  |  |
|  | *Guettarda crispiflora* |  | 54 | 11 |  |  |  |
|  | *Guettarda hirsuta* |  |  | 7 |  |  |  |
|  | *Guettarda pohliana* |  |  |  |  | 2 |  |
|  | *Guettarda roupalifolia* |  | 12 |  |  |  |  |
|  | *Guettarda sanblasensis* |  |  | 3 |  |  |  |
|  | *Isertia laevis* |  |  | 1 |  |  |  |
|  | *Ixora brevifolia* |  |  |  |  | 31 |  |
|  | *Joosia aequatoria* |  |  | 20 |  |  |  |
|  | *Joosia pulcherrima* |  |  | 9 |  |  |  |
|  | *Joosia umbellifera* |  |  | 24 |  |  |  |
|  | *Kutchubaea semisericea* |  |  | 2 |  |  |  |
|  | *Ladenbergia acutifolia* |  |  | 9 |  |  |  |
|  | *Ladenbergia bullata* |  |  |  |  | 3 |  |
|  | *Ladenbergia crassifolia* |  |  |  |  | 2 |  |
|  | *Ladenbergia heterophylla* |  |  | 6 |  |  |  |
|  | *Ladenbergia macrocarpa* |  | 48 | 9 |  |  |  |
|  | *Ladenbergia oblongifolia* |  | 13 | 22 |  | 28 |  |
|  | *Ladenbergia pavonii* |  |  | 2 |  |  |  |
|  | *Ladenbergia riveroana* |  |  | 8 |  |  |  |
|  | *Palicourea acuminata* |  |  | 8 |  |  |  |
|  | *Palicourea amethystina* |  |  | 61 | 8 |  |  |
|  | *Palicourea andaluciana* |  | 30 |  |  |  |  |
|  | *Palicourea andrei* |  |  | 12 |  |  |  |
|  | *Palicourea angustifolia* | 1 |  | 2 |  |  |  |
|  | *Palicourea azurea* |  |  | 2 |  |  |  |
|  | *Palicourea brachiata* |  |  | 17 |  |  |  |
|  | *Palicourea canarina* |  |  | 2 |  |  |  |
|  | *Palicourea conephoroides* |  |  |  | 3 |  |  |
|  | *Palicourea corniculata* |  |  | 3 |  |  |  |
|  | *Palicourea cornigera* |  |  | 1 |  |  |  |
|  | *Palicourea cutucuana* |  |  | 6 |  |  |  |
|  | *Palicourea demissa* |  | 11 | 6 |  |  |  |
|  | *Palicourea garciae* |  | 32 |  |  |  |  |
|  | *Palicourea gomezii* |  |  | 8 |  |  |  |
|  | *Palicourea guianensis* |  | 1 | 1 |  |  |  |
|  | *Palicourea hospitalis* |  |  | 9 |  |  |  |
|  | *Palicourea lineata* |  |  |  | 1 |  |  |
|  | *Palicourea lyristipula* |  |  | 1 |  |  |  |
|  | *Palicourea mitis* |  |  |  | 1 |  |  |
|  | *Palicourea nigricans* |  |  | 1 |  |  |  |
|  | *Palicourea ottonis* |  |  |  |  | 9 |  |
|  | *Palicourea ovalis* |  |  | 5 |  |  |  |
|  | *Palicourea ovata* |  |  |  | 11 |  |  |
|  | *Palicourea palenquensis* |  |  | 1 |  |  |  |
|  | *Palicourea perquadrangularis* |  | 4 |  | 19 |  |  |
|  | *Palicourea stenosepala* |  |  | 13 |  |  |  |
|  | *Palicourea stipularis* |  |  | 2 | 33 | 1 |  |
|  | *Palicourea subtomentosa* |  |  | 6 |  |  |  |
|  | *Palicourea sulphurea* |  |  |  | 3 |  |  |
|  | *Palicourea thyrsiflora* |  | 1 | 12 |  |  |  |
|  | *Palicourea tinctoria* |  |  | 5 | 2 | 5 |  |
|  | *Palicourea ulloana* |  |  | 1 |  |  |  |
|  | *Palicourea weberbaueri* |  |  |  | 19 |  |  |
|  | *Palicourea leuconeura* | 2 |  |  |  |  |  |
|  | *Pentagonia macrophylla* |  |  | 6 |  |  |  |
|  | *Pentagonia spathicalyx* |  |  | 6 |  |  |  |
|  | *Pentagonia wurdackii* |  |  | 1 |  |  |  |
|  | *Pogonopus speciosus* |  | 1 |  |  |  |  |
|  | *Pogonopus tubulosus* |  |  |  |  | 17 | 228 |
|  | *Posoqueria coriacea* |  | 1 | 10 |  |  |  |
|  | *Posoqueria latifolia* |  | 2 | 29 |  |  |  |
|  | *Psychotria berteroana* |  |  | 3 |  |  |  |
|  | *Psychotria carthagenensis* |  |  |  | 2 | 1 |  |
|  | *Psychotria cauligera* |  |  | 2 |  |  |  |
|  | *Psychotria elmeriana* |  |  | 17 |  |  |  |
|  | *Psychotria gracilenta* |  |  | 1 |  |  |  |
|  | *Psychotria micrantha* |  | 1 |  |  |  |  |
|  | *Psychotria microbotrys* |  |  | 2 |  |  |  |
|  | *Psychotria monsalveae* |  | 32 |  |  |  |  |
|  | *Psychotria montivaga* |  |  | 2 |  |  |  |
|  | *Psychotria pichisensis* |  |  | 4 |  |  |  |
|  | *Psychotria trichotoma* |  | 3 |  |  |  |  |
|  | *Randia armata* |  | 7 | 2 | 2 | 3 | 51 |
|  | *Remijia chelomaphylla* |  |  | 20 |  |  |  |
|  | *Ronabea latifolia* |  | 3 |  |  |  |  |
|  | *Rudgea sclerocalyx* |  | 3 |  |  |  |  |
|  | *Rudgea tambillensis* |  |  | 2 |  |  |  |
|  | *Rustia occidentalis* |  | 8 |  |  |  |  |
|  | *Schizocalyx bracteosus* |  | 19 |  |  |  |  |
|  | *Schizocalyx obovatus* |  |  |  |  | 64 |  |
|  | *Simira cordifolia* |  | 3 |  |  |  |  |
|  | *Simira macrocrater* |  |  |  |  | 11 |  |
|  | *Simira rubescens* |  |  | 8 | 8 | 34 |  |
|  | *Stenostomum acreanum* |  | 12 |  |  |  |  |
|  | *Stilpnophyllum oellgaardii* |  |  | 2 |  |  |  |
|  | *Tocoyena williamsii* |  |  | 3 |  |  |  |
|  | *Warszewiczia coccinea* |  | 1 | 5 |  |  |  |
|  | *Warszewiczia uxpanapensis* |  | 21 |  |  |  |  |
| Rutaceae | *Zanthoxylum coco* |  |  |  |  |  | 25 |
|  | *Zanthoxylum comosum* |  |  |  | 1 |  |  |
|  | *Zanthoxylum ekmanii* |  |  |  |  | 1 |  |
|  | *Zanthoxylum fagara* |  |  |  |  | 68 | 22 |
|  | *Zanthoxylum formiciferum* |  |  | 5 |  |  |  |
|  | *Zanthoxylum grandifolium* |  |  | 1 |  |  |  |
|  | *Zanthoxylum lenticulare* |  | 9 |  |  |  |  |
|  | *Zanthoxylum petiolare* |  |  |  |  |  | 136 |
|  | *Zanthoxylum rhoifolium* |  |  |  |  |  | 18 |
|  | *Zanthoxylum riedelianum* |  | 3 | 3 |  |  |  |
|  | *Zanthoxylum sprucei* |  |  |  | 1 |  |  |
|  | *Zanthoxylum melanostictum* | 21 |  | 1 |  |  |  |
|  | *Citrus aurantium* |  |  |  |  |  | 12 |
|  | *Hortia brasiliana* |  | 4 |  |  |  |  |
|  | *Zanthoxylum verrucosum* |  |  | 1 |  |  |  |
| Sabiaceae | *Meliosma arenosa* |  |  | 24 |  |  |  |
|  | *Meliosma boliviensis* |  |  | 1 | 2 |  |  |
|  | *Meliosma echeverryana* |  |  | 1 |  |  |  |
|  | *Meliosma frondosa* |  |  | 4 | 32 | 4 |  |
|  | *Meliosma glabrata* |  |  | 1 |  |  |  |
|  | *Meliosma glossophylla* |  | 1 |  |  |  |  |
|  | *Meliosma herbertii* |  |  | 21 |  | 2 |  |
|  | *Meliosma lanceolata* |  |  |  | 2 |  |  |
|  | *Meliosma occidentalis* |  |  | 3 |  |  |  |
|  | *Meliosma petalodentata* |  |  |  |  | 16 |  |
|  | *Meliosma peytonii* |  |  |  |  | 10 |  |
|  | *Meliosma polyneura* |  |  | 3 |  |  |  |
|  | *Meliosma pumila* |  |  |  | 24 |  |  |
|  | *Meliosma solomonii* |  |  |  |  | 7 |  |
|  | *Meliosma sumacensis* |  |  | 2 |  |  |  |
|  | *Meliosma vasquezii* |  |  | 2 | 1 |  |  |
|  | *Meliosma violacea* |  | 1 | 1 |  |  |  |
|  | *Meliosma herbertii* | 1 |  |  |  |  |  |
|  | *Meliosma pittieriana* | 1 |  |  |  |  |  |
|  | *Ophiocaryon klugii* |  |  | 2 |  |  |  |
| Salicaceae | *Abatia parviflora* |  |  | 3 | 1 |  |  |
|  | *Azara salicifolia* |  |  |  |  |  | 5 |
|  | *Banara guianensis* |  |  | 1 |  |  |  |
|  | *Banara nitida* |  |  | 6 | 1 |  |  |
|  | *Banara regia* |  |  | 7 |  |  |  |
|  | *Banara tomentosa* |  |  |  |  | 72 |  |
|  | *Casearia aculeata* |  | 9 | 1 |  |  |  |
|  | *Casearia acuminata* |  |  | 1 |  |  |  |
|  | *Casearia arborea* |  | 22 | 3 | 4 | 7 |  |
|  | *Casearia arguta* |  | 3 |  |  |  |  |
|  | *Casearia cajambrensis* |  |  | 3 |  |  |  |
|  | *Casearia combaymensis* |  |  | 6 |  |  |  |
|  | *Casearia corymbosa* |  | 4 |  |  |  |  |
|  | *Casearia decandra* |  |  |  | 1 |  |  |
|  | *Casearia fasciculata* |  |  | 4 |  |  |  |
|  | *Casearia gossypiosperma* |  |  |  |  | 47 |  |
|  | *Casearia grandiflora* |  | 2 |  |  |  |  |
|  | *Casearia javitensis* |  | 2 |  |  |  |  |
|  | *Casearia mariquitensis* |  |  | 1 |  |  |  |
|  | *Casearia obovalis* |  |  | 14 |  |  |  |
|  | *Casearia ovata* |  |  |  | 2 |  |  |
|  | *Casearia pitumba* |  |  | 1 |  |  |  |
|  | *Casearia prunifolia* |  |  | 1 |  |  |  |
|  | *Casearia quinduensis* |  |  | 3 |  |  |  |
|  | *Casearia sylvestris* |  |  | 14 |  |  | 11 |
|  | *Casearia tachirensis* |  |  | 17 |  |  |  |
|  | *Casearia zahlbruckneri* |  | 1 |  | 2 |  |  |
|  | *Hasseltia floribunda* |  | 5 | 2 | 18 |  |  |
|  | *Hasseltia lateriflora* |  | 3 |  |  |  |  |
|  | *Laetia procera* |  | 10 | 2 |  |  |  |
|  | *Lunania parviflora* |  | 20 | 10 |  |  |  |
|  | *Macrohasseltia macroterantha* |  | 1 |  |  |  |  |
|  | *Neosprucea grandiflora* |  |  | 3 |  |  |  |
|  | *Pleuranthodendron lindenii* |  |  | 4 |  |  |  |
|  | *Ryania speciosa* |  |  | 2 |  |  |  |
|  | *Tetrathylacium johansenii* |  | 2 |  |  |  |  |
|  | *Tetrathylacium macrophyllum* |  |  | 8 |  |  |  |
|  | *Xylosma longipetiolata* |  |  |  |  |  | 46 |
|  | *Xylosma pubescens* |  |  |  |  |  | 15 |
|  | *Xylosma ruiziana* |  |  | 1 |  |  |  |
|  | *Xylosma velutina* |  |  |  |  | 8 |  |
| Santalaceae | *Acanthosyris annonagustata* |  |  | 4 |  |  |  |
|  | *Acanthosyris falcata* |  |  |  |  |  | 6 |
| Sapindaceae | *Allophylus amazonicus* |  |  | 2 |  |  |  |
|  | *Allophylus divaricatus* |  |  | 1 |  |  |  |
|  | *Allophylus edulis* |  |  |  |  | 4 | 961 |
|  | *Allophylus excelsus* |  |  | 3 |  |  |  |
|  | *Allophylus floribundus* |  |  | 22 |  |  |  |
|  | *Allophylus glabratus* |  |  |  | 2 |  |  |
|  | *Allophylus incanus* |  |  | 4 |  |  |  |
|  | *Allophylus mollis* |  |  | 1 |  | 3 |  |
|  | *Allophylus myrianthus* |  |  |  | 20 |  |  |
|  | *Allophylus petiolulatus* |  |  |  |  | 6 |  |
|  | *Allophylus pilosus* |  |  | 4 |  |  |  |
|  | *Allophylus psilospermus* |  | 2 |  |  |  |  |
|  | *Allophylus punctatus* |  |  | 1 |  | 1 |  |
|  | *Allophylus scrobiculatus* |  |  | 4 |  |  |  |
|  | *Athyana weinmanniifolia* |  |  |  |  |  | 41 |
|  | *Billia rosea* | 77 | 142 | 17 |  |  |  |
|  | *Cupania cinerea* |  |  | 9 |  |  |  |
|  | *Cupania latifolia* |  | 13 |  |  |  |  |
|  | *Cupania livida* |  |  | 2 |  |  |  |
|  | *Cupania rubiginosa* |  |  |  | 13 |  |  |
|  | *Cupania scrobiculata* |  | 1 | 1 |  |  |  |
|  | *Cupania vernalis* |  |  |  |  |  | 275 |
|  | *Diatenopteryx sorbifolia* |  |  |  |  |  | 529 |
|  | *Dilodendron bipinnatum* |  |  |  |  | 1 |  |
|  | *Dilodendron costaricense* |  | 1 |  |  |  |  |
|  | *Matayba adenanthera* |  | 3 |  |  |  |  |
|  | *Matayba arborescens* |  | 49 |  |  |  |  |
|  | *Matayba boliviana* |  |  |  |  | 1 |  |
|  | *Matayba elegans* |  | 6 |  |  |  |  |
|  | *Matayba guianensis* |  |  |  | 2 |  |  |
|  | *Matayba inelegans* |  |  | 12 |  |  |  |
|  | *Matayba macrostylis* |  | 12 |  |  |  |  |
|  | *Paullinia pachycarpa* |  |  | 2 |  |  |  |
|  | *Sapindus saponaria* |  |  |  |  | 14 |  |
|  | *Talisia cerasina* |  | 2 |  | 6 |  |  |
|  | *Talisia hexaphylla* |  | 7 |  |  | 1 |  |
|  | *Talisia microphylla* |  |  | 1 |  |  |  |
|  | *Thinouia compressa* |  |  |  |  | 8 |  |
| Sapotaceae | *Chrysophyllum amazonicum* |  |  | 2 |  |  |  |
|  | *Chrysophyllum argenteum* |  | 51 |  |  |  |  |
|  | *Chrysophyllum cainito* |  | 2 |  |  |  |  |
|  | *Chrysophyllum colombianum* |  | 5 |  |  |  |  |
|  | *Chrysophyllum cuneifolium* |  |  | 2 |  |  |  |
|  | *Chrysophyllum gonocarpum* |  |  |  |  | 24 | 510 |
|  | *Chrysophyllum lanatum* |  |  | 1 |  |  |  |
|  | *Chrysophyllum lucentifolium* |  | 7 |  |  |  |  |
|  | *Chrysophyllum marginatum* |  |  |  |  | 8 | 107 |
|  | *Chrysophyllum sanguinolentum* |  |  | 5 |  |  |  |
|  | *Chrysophyllum venezuelanense* |  | 1 | 9 |  | 95 |  |
|  | *Manilkara chicle* |  | 32 |  |  |  |  |
|  | *Micropholis egensis* |  | 6 | 3 |  |  |  |
|  | *Micropholis guyanensis* |  | 3 | 31 |  |  |  |
|  | *Micropholis melinoniana* |  |  | 6 |  |  |  |
|  | *Micropholis venulosa* |  |  | 5 |  |  |  |
|  | *Pouteria austin-smithii* |  |  | 1 |  |  |  |
|  | *Pouteria baehniana* |  |  | 8 |  | 5 |  |
|  | *Pouteria bangii* |  |  | 3 |  |  |  |
|  | *Pouteria bilocularis* |  |  | 9 |  | 113 |  |
|  | *Pouteria caimito* |  |  | 11 |  | 31 |  |
|  | *Pouteria calystophylla* |  |  | 1 |  |  |  |
|  | *Pouteria capacifolia* |  |  | 17 |  |  |  |
|  | *Pouteria coriacea* |  |  | 1 |  |  |  |
|  | *Pouteria cuspidata* |  | 16 | 9 |  | 7 |  |
|  | *Pouteria durlandii* |  | 16 | 1 |  |  |  |
|  | *Pouteria glomerata* |  | 5 | 2 |  |  |  |
|  | *Pouteria gracilis* |  |  | 1 |  |  |  |
|  | *Pouteria hispida* |  |  |  |  | 10 |  |
|  | *Pouteria krukovii* |  | 3 |  |  |  |  |
|  | *Pouteria lucuma* |  | 3 | 2 |  |  |  |
|  | *Pouteria multiflora* |  | 5 | 8 |  |  |  |
|  | *Pouteria oblanceolata* |  |  | 1 |  |  |  |
|  | *Pouteria platyphylla* |  |  | 1 |  |  |  |
|  | *Pouteria reticulata* |  |  | 1 |  |  |  |
|  | *Pouteria rostrata* |  |  | 2 |  |  |  |
|  | *Pouteria torta* |  | 27 | 49 |  |  |  |
|  | *Pouteria trilocularis* |  |  | 5 | 7 |  |  |
|  | *Pouteria vernicosa* |  |  | 2 |  |  |  |
|  | *Sarcaulus brasiliensis* |  |  | 2 |  | 129 |  |
|  | *Sarcaulus oblatus* |  |  | 4 |  |  |  |
|  | *Sideroxylon obtusifolium* |  |  |  |  | 51 |  |
| Simaroubaceae | *Picramnia juniniana* |  |  | 1 |  |  |  |
|  | *Picrolemma huberi* |  | 6 |  |  |  |  |
|  | *Simaba paraensis* |  |  | 1 |  |  |  |
|  | *Simarouba amara* |  | 6 | 5 |  | 2 |  |
| Siparunaceae | *Siparuna aspera* |  |  | 11 |  |  |  |
|  | *Siparuna cervicornis* |  |  | 1 |  |  |  |
|  | *Siparuna cuspidata* |  |  | 3 |  |  |  |
|  | *Siparuna echinata* |  |  | 10 |  |  |  |
|  | *Siparuna eggersii* |  |  |  | 11 |  |  |
|  | *Siparuna gentryana* |  | 1 |  |  |  |  |
|  | *Siparuna harlingii* |  |  | 2 |  |  |  |
|  | *Siparuna lepidota* |  |  | 1 |  |  |  |
|  | *Siparuna macrotepala* |  |  | 1 |  |  |  |
|  | *Siparuna muricata* |  |  | 2 |  |  |  |
|  | *Siparuna pilosolepidota* |  |  | 4 |  |  |  |
|  | *Siparuna schimpffii* |  |  | 1 |  |  |  |
|  | *Siparuna thecaphora* |  |  |  |  | 1 |  |
|  | *Siparuna tomentosa* |  |  | 1 |  |  |  |
| Solanaceae | *Cestrum conglomeratum* |  |  |  | 1 |  |  |
|  | *Cestrum humboldtii* |  |  | 5 |  |  |  |
|  | *Cestrum megalophyllum* |  |  | 1 |  |  |  |
|  | *Cestrum peruvianum* |  |  |  | 19 |  |  |
|  | *Cestrum schlechtendahlii* |  |  | 5 |  |  |  |
|  | *Cestrum tomentosum* |  |  | 4 |  |  |  |
|  | *Cestrum vestitum* |  |  | 1 |  |  |  |
|  | *Cestrum lindenii* | 1 |  |  |  |  |  |
|  | *Eriolarynx lorentzii* |  |  |  |  |  | 64 |
|  | *Iochroma calycinum* |  |  | 1 |  |  |  |
|  | *Iochroma cornifolium* |  |  | 1 |  |  |  |
|  | *Saracha punctata* |  |  |  | 5 | 23 |  |
|  | *Seesea crasivenosa* |  |  | 7 |  |  |  |
|  | *Solanum abitaguense* |  |  | 1 |  |  |  |
|  | *Solanum aligerum* |  |  |  |  |  | 149 |
|  | *Solanum altissimum* |  |  | 1 |  |  |  |
|  | *Solanum anisophyllum* |  |  | 2 |  |  |  |
|  | *Solanum aphyodendron* |  |  |  |  | 4 |  |
|  | *Solanum asperolanatum* |  | 1 | 5 |  |  |  |
|  | *Solanum aturense* |  |  | 3 |  |  |  |
|  | *Solanum barbulatum* |  |  | 1 | 13 |  |  |
|  | *Solanum clandestinum* |  |  |  |  | 17 |  |
|  | *Solanum cutervanum* |  |  |  | 9 | 1 |  |
|  | *Solanum grandiflorum* |  |  | 1 |  |  |  |
|  | *Solanum inelegans* |  |  |  |  | 1 |  |
|  | *Solanum lepidotum* |  | 2 |  |  |  |  |
|  | *Solanum maturecalvans* |  |  |  | 40 | 25 |  |
|  | *Solanum nutans* |  |  | 6 | 2 |  |  |
|  | *Solanum oblongifolium* |  |  | 8 |  |  |  |
|  | *Solanum occultum* |  |  | 1 |  |  |  |
|  | *Solanum ochrophyllum* |  |  |  |  | 138 |  |
|  | *Solanum riparium* |  |  |  |  |  | 275 |
|  | *Solanum sessile* |  |  | 4 |  |  |  |
|  | *Solanum stenophyllum* |  |  | 2 |  |  |  |
|  | *Solanum trichoneuron* |  |  |  |  |  | 46 |
|  | *Solanum umbellatum* |  |  | 1 |  |  |  |
|  | *Solanum venosum* |  |  | 1 |  |  |  |
|  | *Vassobia breviflora* |  |  |  |  |  | 94 |
| Staphyleaceae | *Turpinia occidentalis* | 1 | 36 | 37 | 10 |  |  |
| Stemonuraceae | *Discophora guianensis* |  | 1 |  |  |  |  |
| Styracaceae | *Styrax cordatus* |  |  | 2 | 47 |  |  |
|  | *Styrax davillifolius* |  | 7 |  |  |  |  |
|  | *Styrax foveolaria* |  |  | 3 | 10 |  |  |
|  | *Styrax guyanensis* |  |  | 1 |  |  |  |
|  | *Styrax nunezii* |  |  |  |  | 1 |  |
|  | *Styrax pentlandianus* |  | 1 |  | 1 | 2 |  |
|  | *Styrax subargenteus* |  |  |  |  |  | 78 |
|  | *Styrax tomentosus* |  | 2 | 7 |  |  |  |
| Symplocaceae | *Symplocos baehnii* |  |  |  | 1 |  |  |
|  | *Symplocos bogotensis* |  |  | 6 |  |  |  |
|  | *Symplocos canescens* |  |  | 7 | 7 |  |  |
|  | *Symplocos carmencitae* |  |  | 2 |  |  |  |
|  | *Symplocos colorata* |  |  |  |  | 6 |  |
|  | *Symplocos coriacea* |  |  | 7 |  |  |  |
|  | *Symplocos debilis* |  |  |  |  | 5 |  |
|  | *Symplocos denticulata* |  |  |  |  | 2 |  |
|  | *Symplocos fimbriata* |  |  |  |  | 28 |  |
|  | *Symplocos flosfragrans* |  | 4 |  |  |  |  |
|  | *Symplocos fuliginosa* |  |  | 9 |  |  |  |
|  | *Symplocos fuscata* |  |  | 10 |  |  |  |
|  | *Symplocos mapiriensis* |  |  |  |  | 43 |  |
|  | *Symplocos melanochroa* |  |  |  | 1 |  |  |
|  | *Symplocos mezii* |  |  |  | 5 |  |  |
|  | *Symplocos nuda* |  |  | 17 |  |  |  |
|  | *Symplocos phaeoneura* |  | 1 |  |  |  |  |
|  | *Symplocos pilosa* |  |  | 1 |  |  |  |
|  | *Symplocos pluribracteata* |  |  | 2 |  |  |  |
|  | *Symplocos psiloclada* |  |  |  | 57 |  |  |
|  | *Symplocos quindiuensis* |  | 5 |  |  |  |  |
|  | *Symplocos quitensis* |  |  | 71 | 87 | 52 |  |
|  | *Symplocos reflexa* |  |  | 1 | 28 |  |  |
|  | *Symplocos robusta* |  |  |  |  | 22 |  |
|  | *Symplocos spruceana* |  |  | 3 | 3 |  |  |
|  | *Symplocos amplifolia* | 2 |  |  |  |  |  |
| Tapisciaceae | *Huertea glandulosa* |  |  | 29 |  |  |  |
|  | *Huertea granadina* |  | 18 |  |  |  |  |
| Theaceae | *Gordonia fruticosa* | 16 | 13 | 47 | 55 | 303 |  |
|  | *Gordonia pubescens* |  | 11 |  |  |  |  |
| Thymelaceae | *Daphnopsis equatorialis* |  |  | 1 |  |  |  |
| Trigoniaceae | *Isidodendron tripterocarpum* |  | 22 |  |  |  |  |
| Ulmaceae | *Ampelocera edentula* |  |  | 3 |  |  |  |
|  | *Ampelocera longissima* |  | 19 | 16 |  |  |  |
|  | *Ampelocera macrocarpa* |  | 5 |  |  |  |  |
|  | *Ampelocera ruizii* |  |  |  |  | 26 |  |
|  | *Phyllostylon rhamnoides* |  |  |  |  | 338 | 653 |
| Urticaceae | *Boehmeria caudata* |  |  | 3 |  |  | 28 |
|  | *Boehmeria radiata* |  |  | 8 |  |  |  |
|  | *Cecropia andina* |  |  | 12 |  |  |  |
|  | *Cecropia angustifolia* |  |  | 46 | 17 | 10 |  |
|  | *Cecropia bullata* |  | 1 |  |  |  |  |
|  | *Cecropia engleriana* |  |  | 3 |  |  |  |
|  | *Cecropia ficifolia* |  |  | 7 |  |  |  |
|  | *Cecropia gabrielis* |  | 6 | 14 |  |  |  |
|  | *Cecropia garciae* |  |  | 2 |  |  |  |
|  | *Cecropia herthae* |  |  | 6 |  |  |  |
|  | *Cecropia marginalis* |  |  | 35 |  |  |  |
|  | *Cecropia maxima* |  |  | 27 |  |  |  |
|  | *Cecropia membranacea* |  | 11 | 6 |  | 11 |  |
|  | *Cecropia montana* |  |  | 50 |  |  |  |
|  | *Cecropia obtusifolia* |  | 24 |  |  |  |  |
|  | *Cecropia peltata* |  | 58 |  |  |  |  |
|  | *Cecropia polystachia* |  |  |  | 8 |  |  |
|  | *Cecropia putumayonis* |  |  | 3 |  |  |  |
|  | *Cecropia reticulata* |  | 2 | 3 |  |  |  |
|  | *Cecropia sciadophylla* |  |  | 45 |  |  |  |
|  | *Cecropia tacuna* |  |  |  |  | 14 |  |
|  | *Cecropia telenitida* |  | 5 | 40 |  |  |  |
|  | *Cecropia utcubambana* |  |  | 4 |  |  |  |
|  | *Coussapoa cinnamomifolia* |  |  |  |  | 1 |  |
|  | *Coussapoa contorta* |  |  | 1 |  |  |  |
|  | *Coussapoa crassivenosa* |  |  | 3 |  | 1 |  |
|  | *Coussapoa herthae* |  |  | 2 |  |  |  |
|  | *Coussapoa manuensis* |  |  |  |  | 4 |  |
|  | *Coussapoa napoensis* |  |  | 2 |  |  |  |
|  | *Coussapoa orthoneura* |  |  | 15 |  |  |  |
|  | *Coussapoa ovalifolia* |  |  | 2 | 1 | 6 |  |
|  | *Coussapoa villosa* |  |  | 18 |  |  |  |
|  | *Myriocarpa stipitata* |  | 3 | 9 |  |  | 190 |
|  | *Pourouma bicolor* |  | 143 | 50 |  | 53 |  |
|  | *Pourouma cecropiifolia* |  |  | 68 | 8 |  |  |
|  | *Pourouma cucura* |  |  | 1 |  |  |  |
|  | *Pourouma floccosa* |  |  | 2 |  |  |  |
|  | *Pourouma guianensis* |  |  | 32 |  | 15 |  |
|  | *Pourouma hirsutipetiolata* |  |  | 2 |  |  |  |
|  | *Pourouma minor* |  |  | 10 | 1 | 30 |  |
|  | *Pourouma mollis* |  |  | 7 |  | 3 |  |
|  | *Pourouma montana* |  |  | 6 |  |  |  |
|  | *Pourouma napoensis* |  |  | 1 |  |  |  |
|  | *Pourouma petiolulata* |  |  | 1 |  |  |  |
|  | *Pourouma tomentosa* |  |  | 5 |  |  |  |
|  | *Urera baccifera* |  | 3 |  |  | 122 | 395 |
|  | *Urera capitata* |  |  |  |  | 3 |  |
|  | *Urera caracasana* |  | 4 | 40 |  |  | 442 |
|  | *Urera laciniata* |  |  |  |  | 1 |  |
|  | *Urera verrucosa* |  |  |  |  | 11 |  |
|  | *Uribea tamarindoides* |  | 2 |  |  |  |  |
| Verbenaceae | *Citharexylum joergensenii* |  |  |  |  |  | 16 |
|  | *Citharexylum laurifolium* |  |  |  |  | 9 |  |
|  | *Citharexylum montanum* |  |  | 23 |  |  |  |
|  | *Duranta serratifolia* |  |  |  |  |  | 127 |
|  | *Duranta sprucei* |  |  |  |  | 2 |  |
|  | *Lippia hirsuta* | 1 |  |  |  |  |  |
| Violaceae | *Gloeospermum equatoriense* |  |  | 6 |  |  |  |
|  | *Gloeospermum grandifolium* |  |  | 2 |  |  |  |
|  | *Gloeospermum longifolium* |  | 3 | 9 |  |  |  |
|  | *Gloeospermum sphaerocarpum* |  | 2 |  |  |  |  |
|  | *Leonia crassa* |  |  | 24 |  |  |  |
|  | *Leonia glycycarpa* |  |  | 21 | 2 |  |  |
|  | *Leonia triandra* |  | 12 |  |  |  |  |
|  | *Pombalia communis* |  |  |  |  | 1 |  |
|  | *Rinorea apiculata* |  |  | 28 |  |  |  |
|  | *Rinorea guianensis* |  |  |  | 20 |  |  |
|  | *Rinorea hirsuta* |  | 3 |  |  |  |  |
|  | *Rinorea paniculata* |  | 34 |  |  |  |  |
|  | *Rinorea viridifolia* |  |  |  |  | 5 |  |
| Vochysiaceae | *Erisma uncinatum* |  |  | 3 |  |  |  |
|  | *Qualea lineata* |  | 3 |  |  |  |  |
|  | *Vochysia biloba* |  |  | 3 |  |  |  |
|  | *Vochysia braceliniae* |  |  | 5 |  |  |  |
|  | *Vochysia ferruginea* |  | 13 | 1 |  |  |  |
|  | *Vochysia grandis* |  |  | 4 |  |  |  |
|  | *Vochysia megalantha* |  | 1 |  |  |  |  |
|  | *Vochysia punctata* |  |  | 3 |  |  |  |
|  | *Vochysia aurantiaca* |  |  | 8 |  |  |  |
| Winteraceae | *Drimys granadensis* |  | 2 | 14 |  |  |  |
| Ximeniaceae | *Ximenia americana* |  |  |  |  | 2 |  |
| **TOTAL** |  | **946** | **9106** | **16884** | **10909** | **18541** | **30578** |
