# Supplementary material for: Elevation and latitude drives structure and tree species composition in Andean forests: Results from a large-scale plot network
Source: PLoS One. 2020 Apr 20;15(4):e0231553. doi: 10.1371/journal.pone.0231553 (PMC7170706; doi:10.1371/journal.pone.0231553)
Supplement: S3 Appendix — Summary of Linear Mixed-effects Model (LMM) for Bray Curtis distance between pairs of forest plots, varying intercept by latitudinal bands every 5°. Final model fitted by REML. SD = Standard deviation, VE = Variance explained (i.e. marginal and conditional R2). (DOCX) [file pone.0231553.s004.docx]

**Appendix 3.** **Linear mixed-effects model**. Summary of Linear mixed-effects model (LMM) for Bray Curtis distance between pairs of forest plots, varying intercept by latitudinal bands every 5°. Final model fitted by REML. SD = Standard deviation, VE = Variance explained (i.e. marginal and conditional R^2^).

| **Fixed effects** | **Estimate** | **SD** | **t-value** | **VE (%)** |
| --- | --- | --- | --- | --- |
| Intercept | 0.131 | 0.013 | 10.1 | 2.1 |
| Geographical distance | -0.011 | 3.9e-04 | -26.9 |  |
| Elevational distance | -3.8e-05 | 7.3e-07 | -51.6 |  |
| Random effects |  |  |  |  |
| Latitudinal bands |  | 0.034 |  | 30.7 |
| Residual |  | 0.075 |  | 67.2 |
