# Supplementary material for: Elevation and latitude drives structure and tree species composition in Andean forests: Results from a large-scale plot network
Source: PLoS One. 2020 Apr 20;15(4):e0231553. doi: 10.1371/journal.pone.0231553 (PMC7170706; doi:10.1371/journal.pone.0231553)
Supplement: Attachment — Submitted filename: PONE-D-19-30260_First_ReviewDec2019.pdf [file pone.0231553.s005.pdf]

Manuscript Review
PLOS ONE (PONE-D-19-30260)
Structural and compositional trends along elevation and latitude gradients of Andean forests
By Malizia et al…

General comments:

This study is based on data collected from an impressive number of research sites, between temporary and permanent forest plots, located in different parts of the Andes in South America, an important biological hotspot that has received less attention compared to lowland forests. Broadly, this paper takes advantage of the wide and complex environmental range covered by the dataset to address the questions of the combined effects of elevation and latitude on the structure and tree species composition of Andean forests. By means of generalized models, the authors found that forest density (i.e. number of trees or stems per unit of area) increased with elevation with an opposite effect from latitude. The differences in species richness found between tropical and subtropical Andes are not surprising and are aligned with previous studies. However, the degree of similarity (or dissimilarity) in terms of species diversity in these two broad regions within the Andes was an interesting finding. The authors very well highlight the immense value that this Andean plot-network (i.e. *Red de Bosques Andinos*) has for strengthening scientific research in the region and promote collaboration. These are not 'novel' questions but given the amount of data used, especially compared to an earlier study from the same group (Baez et al. 2015 PLoS ONE), I find the paper of high interest for many scientists and forest ecologists, and readers of PLoS ONE. **However, in its current form this manuscript remains largely descriptive and a better explanation of the causal mechanisms behind how elevation and/or latitude drives structure and composition is missing. The implications of the results to other aspects of forest structure (e.g. carbon) or dynamics (e.g. stem turnover) could be added too. I think the paper should be improved, especially in the discussion section, before being considered for publication. I am pointing out several comments, questions, and occasionally, a few suggestions, that could help moving this paper forward.**

Typesetting and formatting:

I don't normally pay too much attention to these aspects at this stage. However, I strongly recommend the paper by checked for type setting errors, and the written structure of some paragraphs is reviewed to help the reader. Details are offered below indicating page and line numbers. **Yet, as a direct note to the authors, in the pdf version I had access to there was no line number after page 13 of the document (after Table 1), and at line 275 (Table 1), the page number starts again with 1. For this section then I will be referring my comments using these numbers while trying to point out the paragraph number and a few words in the sentence to facilitate the review process.**

Title:

I suggest the title to be more directly linked with the results (e.g. *Elevation and latitude drives structure and tree species composition in Andean forests*). As I will mention later, the manuscript includes in the discussion a long portion about the value of the plot network that I am not sure if is relevant. However, if the authors decide to keep it, maybe a change in the title should also reflect this (e.g. *Elevation and latitude drives structure and tree species composition in Andean forests: results from a large-scale plot network*).

Abstract:

This section needs to be modified a bit. In particular, the reader will benefit from having more specific results being mentioned in the abstract. For example, there is an important aspect of the study related to plot size and its effects on the results that is not mentioned here.

Page 4, Lines 68 - 69: "…Here, we assessed patterns of Andean forest tree structure and diversity along ~ 4000 km of latitude and ~ 4000 m of elevation range…"

Consider changing to: "…**In this study, we assessed patterns of structure and tree species diversity across a wide altitudinal and latitudinal range in Andean forests**…"

Page 4, Line 75: "…Subtropical forests have distinct composition from tropical forests..."

Consider changing to: "…Subtropical forests have distinct **tree species composition compared to those in the tropical region**…"

Introduction:

Page 4, L83: "Indeed, the Tropical Andes…". The region is already mentioned in the first sentence. Just start the line with something like "…Indeed, this region is one of the most diverse terrestrial hotspots on earth [1]…"

Page 5, L86: "…that have not been described yet…" Change to: "…that have not **yet** been described…".

P5 L87-90: There are two different sentences, with different citations too, that mention "climate regulation". Unify or combined these statements avoiding repetition.

P5 L92-95: Again, two sentences discuss similar aspects, specifically about social aspects (i.e. "population growth" and "economic, and cultural factors"). Consider simplifying this paragraph. Also, is not clear what "contrasting patterns of human population growth" means? Please clarify.

P5 L96: "Changes to Andean forests…" Changes in what? Forest cover? Structure? I don't think the word "patterns" fits here. Consider something like: "…Changes in Andean forest cover includes both forest expansion, mostly frequent above 2000 masl) and deforestation that often dominates at lower elevations [12]…"

P5 L102: "…are already undergoing…" Change to: "…**experiencing an apparent shift** in species composition (i.e…)"

P5 L104: Are these predicted shifts for plant species, animal species? Please clarify.

P6 L109-114: I am not sure if this whole paragraph fits here. Are those "knowledge gaps" related to the study? I understand the importance of collaborative research networks but the next paragraph (L116) immediately start discussing the effects of elevation on tree density. Consider removing this paragraph or in any case a connecting sentence is needed before you jump to the density portion.

P6 L121 "…subtropical section of the Andes…" Just say" "subtropical Andes…"

P6 L128: "Species richness…" All species? Plants? Clarify.

P7 L134: "…also showed…" -- > "…also **shows** the…"

P7 L135: "…being maximum…" -- > "…**reaching a** maximum at…"

P7 L136: "…Argentina [38], decreasing…" -- > "…Argentina [38], **while** decreasing above…"

P7 L138-140: I agree that long-term monitoring is needed, but the study do not use or report temporal trends so how is this relevant here? Consider moving to the conclusions.

P7 L142: "…the main structural and diversity patterns…" -- > "…**the main patterns of structure and tree species diversity in Andean forest communities**…"

P7 L144: Why consider species richness as a structural component?

P7 L148-150: "…This study…" This paragraph would fit better perhaps at the beginning of this section in L142. Also, is not clear what do you mean about "…at regional scales in global context..."? Perhaps just say "…This study is one of the first attempts to describe and characterize regional patterns of forest structure and diversity in the Andes across (go to L143)…"

Two major points here: 1) I think is important to highlight how is this study different from the Baez et al study? I understand that in the 2015 paper there was no consideration about diversity and here you use a much higher number of plots. Thus, I see the need for making this point clear; 2) I think at the end of the introduction some general hypotheses would be useful. What were the patterns expected considered earlier evidences?

<u>Materials and Methods</u>:

P8 L155: "…Paleogene (). Subsequent…" Change to "…Paleogene (), **and** subsequent collisions…".

P8 L156: The reference of Hoorn et al, 2010 does not follow PLoS guidelines (i.e. []).

P8 L157: "…late middle…" -- > "…late **mid** Miocene…"

P8 L160: A citation is needed for this paragraph after "…Amazon ecosystems…".

P8 L164: "…biodiversity and distribution…" -- > "…biodiversity **and species** distributions…"

P8 L169: "…vary gradually…" how? What does the word gradually means? I think the paragraph that starts later in L180 of page 9 with "...The mountain forest ecosystem…" should be moved here in support of this initial statement. In relation to this, I liked Figure 1, but I also thinks it deserves a better explanation, either in the main text or at least as an expanded figure caption. Why these variations in forest architecture? Some lines on temperature and radiation effects would be useful.

P9 L194: Consider using "is" instead of "has been" when referring to the objective of the network.

P9 L196-197: Delete "the" before exchange, development, strengthening.

P10 L205-212. This whole section and the next (L215 to 212) about the protocols can be combined, simplified and shortened.

P11 L226: "…events and recorded and the DBH of marked trees are…" -- > "…events **are** recorded and the DBH of marked trees **is** remeasured…"

P11 L230-241: I understand the importance of this section and I hugely support collaboration and networking. I am trying to find sections that could be simplified to allow for more space for discussion and I think this is definitely one of those parts. This paragraph here seems out of place. It could be shortened and added to the background section, used in a new appendix to fully describe the work of the Andean network or simply removed. As mentioned earlier when reviewing the title of the paper, if the goal is to highlight the value of the network a different approach would be useful, where a more in-depth discussion about research collaboration and networking is part of the manuscript.

P12 L247: Are these values in precipitation 1901 ± 903 refers to the mean and SD? Clarify.

P12 L248: "…mature forest but the Network also includes a few older…" -- > "…mature forest**s** but the **n**etwork also includes **several** older…

P12 L250-252: This statement about the data being available is out of place. This should be part of the data availability statement that is one of the specific sections asked by PLoS. Also, with regards to this there are three different statements:

In Data availability the authors express: *Yes - all data are fully available without restriction* Later, when answering the questions on where this data could be found the authors expressed "*All relevant data are within the manuscript and its Supporting Information files…*". Delete the portion in the main text and unify these statements clearly.

P12 L254: Again, the values in plot size refers to mean and SD? I don't understand 0.32 ± 0.47 ha?. How is this possible?. The same observation applies to the subsequent parts where plot size is mentioned. Also, I don't think is necessary to put ± 0 whenever the plot size remains constant in some regions/countries.

P12 L261: "…being the longest subset…" -- > "…being the longest **known** subset…"

P13 L270 or caption in Figure 2:. Please clarify what do you mean when you say "…One (1) census refers to the establishment of a permanent plot"? I understand a one census plot as that with only one measurement, whether temporal or permanent.

L275 Table 1 (No page number!): It is not clear to me that if the range shown for plot area also correspond to the range in basal area. That is for example: Row 1 in the table (AR). A 0.16 ha plot accounts for 78 stems and 5 m^2 in BA, while the 6-ha plot is linked to 1834 stems and 189.4 m^2? Why not just simply express everything in 1 ha scale? I understand the issues of extrapolation, especially from really small plots, but yet you still perform the analysis later. Please clarify.

***** Starting here there is no line number, and page number is 2 for the section where "Data analyses" subtitle is included. I'm using these numbers and will add the "p" for paragraph.**

P2 paragraph 1: "…resolution that represents the…" -- > "…resolution **covering** the average…"

P2 p1: "…from 1 C for Ecuador…" -- > "from 1 C **in** Ecuador…" Same for the rest of the sentences where a similar text is used (i.e. change "for" to "in").

<u>Data Analyses</u>

P2 p2: Delete "In order to…" Just simply say "To analyze…"
"…(n = 236) and stems…" -- > "…(n = 236) where stems ≥ 10 cm DBH were measured. Due to high variability and dispersion when extrapolating basal area and stem density…we discarded plots of 0.01 to 0.08 ha in size (total n = 255)...".

Related to this part of the analytical approach, I am wondering how different are the extrapolations when you compare a 0.08 ha plot and a 0.1 one? You have discarded quite a lot of plots using this cut-off (which I am ok with) but I asked myself if maybe just 0.05 ha would have been enough?

Citation [55] should be placed after the (GLM) portion as this work refers to this modeling approach.

"Considering the nature of the response variables…" what is that nature? Statistical distribution? Did you perform a distribution test ahead of the GLMs? Please clarify.

P3 p2: Delete "In order to…" -- > "**To analyze patterns of tree species composition and…, we used all data available from all plots, and from all species fully identified (2341) with stems ≥ 10 cm DBH. To describe species composition, we applied a Non-Metric Multidimensional Scaling approach…**"

[…[61] using latitudinal bands as random factor…" -- > "…as **a** random factor…"

P4: Include citations for all R packages used.

Results:

P4 p2: "… Mean annual air temperature and rainfall were positively correlated ($r = 0.46$, $p < 0.001$) (Fig 3B)…" I trust the numbers but the correlation in the figure seems to be driven by a few really wet points. Can you clarify this? Adding a trend line would be useful too. Using green and red dots here makes difficult to tease apart the countries. I suggest using an alternate color palette and perhaps different symbols for each country.

P5 p1: Delete "Considering plots…" since is already mentioned in the methods. Just start with "we found that stem density…"

With regards to this section and Figure 4, the small-sized plots seem to be creating a lot of noise in the trends. Why not testing stem density and basal area for only 1-ha plots as shown for species richness in Fig 4G? Also discussed later, are the plots with higher stem density also showing high basal area? There is a brief mention in the discussion about this relationship, but a simple bivariate plot would help.

P6 p2: "…we registered a total of 97,054 tree stems'…" The abstract mentions 86,964 individuals (?). Also, are these all tree species? When authors say stems it might imply other life forms as palms or tree-ferns. Please clarify.

P6 p2: "…was shared among all data sets…" -- > "…was shared among **all plots**…"

P6 p3: "The study plots tended to segregate along…" Use a different word for segregate: cluster, group.

P6 p3: "…Elevation and latitude correlated with both…" -- > "…correlated **well** with…". Also, in Figure 5 could you explain what does each axis in the NMDS represent?

<u>Discussion and conclusions</u>: See **in bold** edits and suggestions.

An overall recommendation here is that for every sentence/statement where the authors are highlighting a specific result, having a direct reference to the particular figure or table where the reader can refer to again would be quite useful. Also, I think authors can separate the conclusions here.

P9 p1: "…it was less expected for rainfall as [5]…" Change the structure of the sentence and citation format to **"…it was less expected for rainfall as Urrutia et al [5] reported for the Andes where rainfall did not covaried linearly with elevation…".** Also, this statement relates to an earlier observation about the need for some hypothesis statements. If you say "it was less expected", what were your initial expectations?

P9 p2: "Both stem density and basal area were related with the gradients addressed…" -- > "**Both stem density and basal area were related to the gradients covered by our dataset…**"

"…patterns of stem density were more consistent with elevation and latitude than **for** basal area…"

Here, authors repeat some sentences from the results section. "Stem density peaked at 10-15 latitude".

"The observed increase **in the mean values of the structural variables** with elevation **has** not…"

P10 p1: "…or maybe some local climate and topographic conditions…" This sounds vague. What other climate or topographic conditions might have influenced these results? Explain. Land-use history is briefly mentioned as a potential driver of stem density. Yet, the authors have some (limited) information on the time since disturbance for some of the plots. Why not discuss this better? Can you filter some of the results based on different disturbance periods? Very dense plots might be a reflection of recent (or not that recent) events. At least in the form of an Appendix this would add some support when contrasting such a wide range of sites and conditions.

P10 p1: Change "border effect" for "edge effect". Also, briefly explain why these effects are more pronounced for small plots?. Why plots ~0.5 ha or larger might be preferred? This section discussing potential effects of plot size also needs some citations. See for example: Wagner et al. 2010 Biotropica, Volume 42 (6): 664-671 and some references therein.

P10 p2: I think only mentioning the main hypotheses (e.g. Janzen & Connel) is not enough here. How these hypotheses are related to the results found?

P11 p2: "…**plots associated with low diversity of tree species**…"

P11 p2: Change the reference format for Kattan et al. 2004 accordingly.

P11 p3: Delete "As well…"

P12 p1: "…are unknown…" change to -- > " are **not clear** [36]"

There is quite an abrupt change from this last paragraph to the next that discuss the importance of the Andean forest plot network. What are some of the potential implications of the findings? What does it mean that some forests in the Andes have higher density than others? More carbon? Less carbon? What about diversity? Are these forests well protected? I am not asking

for a detailed analysis but just a brief consideration to potential links of the results to management or conservation aspects.

P12 p2: The enumeration used in this long paragraph is confusing. There are two sections discussing different aspects, yet, some are redundant (e.g. #4 and #8 about models). Consider rewriting.

P12 p2: "…improving the number of hectares per country…" **-- >** **"…increasing the area covered per country…**"
